# Supplementary material for: Computational Modeling of Low-Abundance Proteins in Venom Gland Transcriptomes: Bothrops asper and Bothrops jararaca
Source: Toxins (Basel). 2025 May 22;17(6):262. doi: 10.3390/toxins17060262 (PMC12197698; doi:10.3390/toxins17060262)
Supplement: Supplementary file 1 [file toxins-17-00262-s001.zip › Supplementary Material 1/Supplementary Materials 1.pdf]

Computational Modeling of Low-Abundance Proteins in Venom Gland Transcriptomes: *Bothrops asper* and *Bothrops jararaca*

Joseph Espín-Angulo and Doris Vela*

Supplementary Material 1: Multiple Alignment

1. CRISP

1.1. Multiple alignment analysis

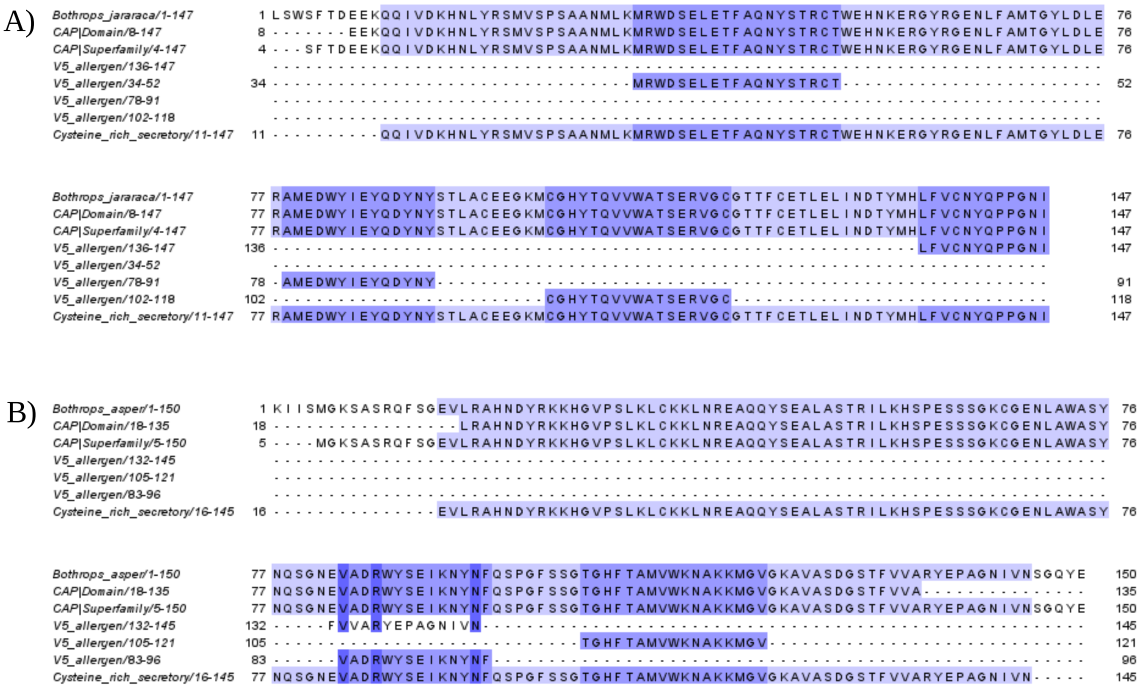

FS1. Multiple alignment of PFAM results for allergen 5. A) *Bothrops asper* and B) *Bothrops jararaca*. Identification of the CAP and CRISP domain, and three conserved sites of allergen 5.

2. von Willebrand factor type D

2.1. Multiple alignment analysis

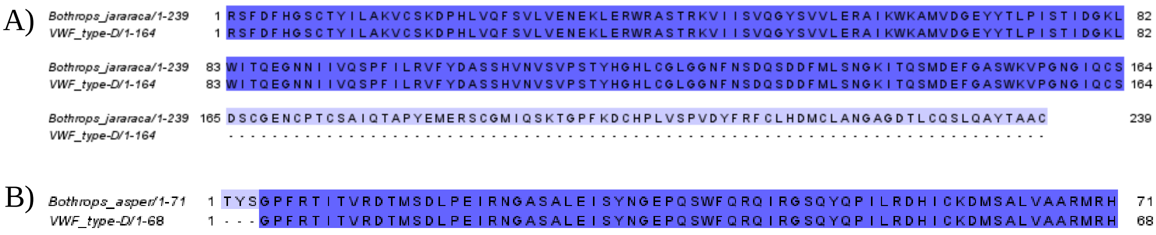

FS2. Multiple alignment of PFAM results for von Willebrand factor type D. A) *Bothrops asper* and B) *Bothrops jararaca*. Identification of the von Willebrand factor type D domain.

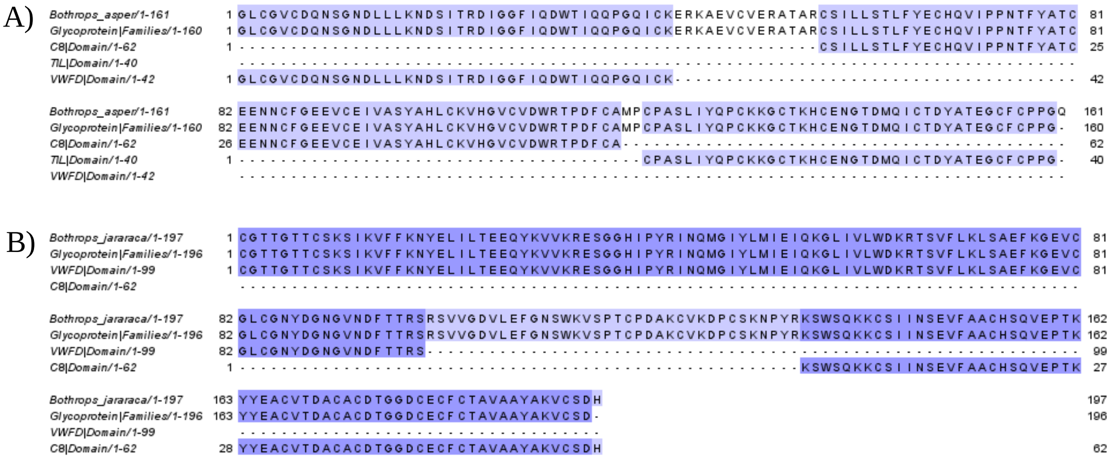

FS3. Multiple alignment analysis of PFAM results for von Willebrand factor type D. A) Annotated sequences for *Bothrops asper*. B) Annotated sequences for *Bothrops jararaca*. Identification of the von Willebrand factor D-type domain, C8 domain, TIL domain, and the glycoprotein family. This figure summarizes the presence and location of conserved domains based on analysis with the PFAM database.

2.2. Structural analysis of disulfide bridges for Bothrops asper

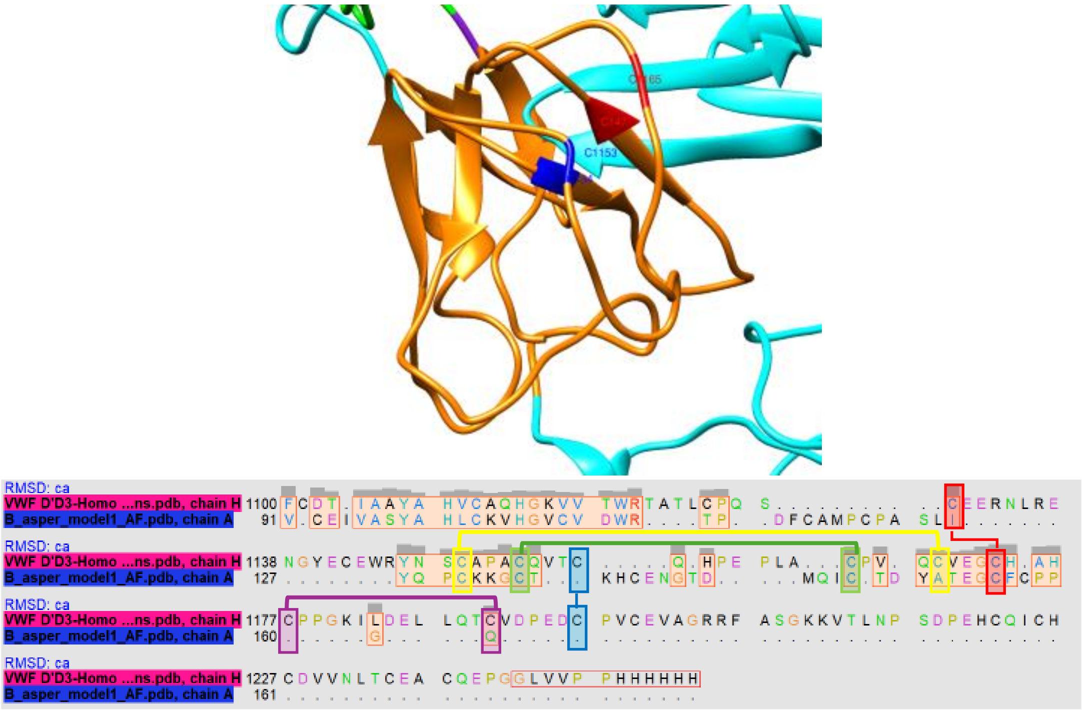

FS4. Structure of the apolipophorin TIL domain in *Bothrops asper*. Structural alignment performed in CHIMERA between the human vWFD domain model [PDB 2MHP] and the predicted sequence in *B. asper*. The identified disulfide bridges are highlighted: Cys1130–Cys1173 (red), Cys1149–Cys1169 (yellow), Cys1153–Cys1165 (green), Cys1157–Cys1196 (blue), and Cys1177–Cys1190 (purple). Only the Cys1153–Cys1165 pair is conserved in humans, equivalent to Cys134–Cys147 in *B. asper*.

2.3. Structural analysis of disulfide bridges for Bothrops jararaca

A)

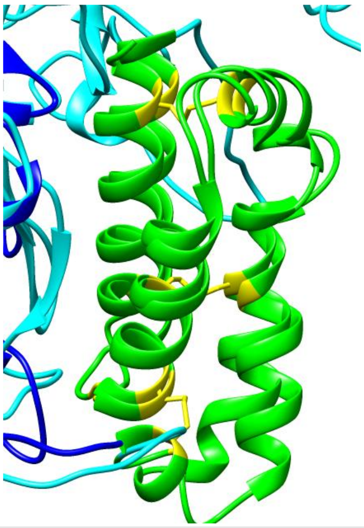

B)

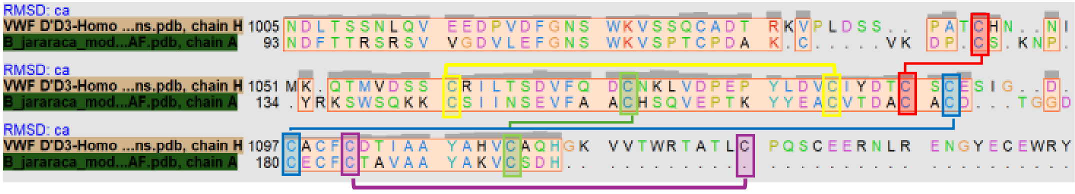

FS5. Structure of the C8 domain of apolipophorin. A) Comparison of the C8 domain of the *Homo sapiens* model and the *Bothrops jararaca* model. B) CHIMERA alignment of the *Homo sapiens* vWFD model [2MHP] with the *Bothrops jararaca* model. The disulfide bridges are represented: Cys1046–Cys1089 (red), Cys1060–Cys1084 (yellow), Cys1071–Cys1111 (green), Cys1091–Cys1097 (blue), and Cys1177–Cys1190 (purple).

3. Arylsulfatase

3.1. Structural analysis of the sulfatase motif

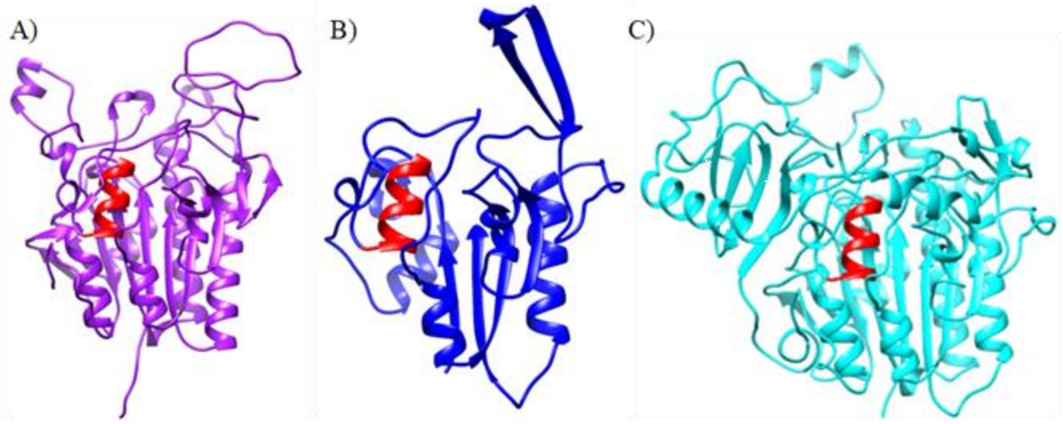

D)

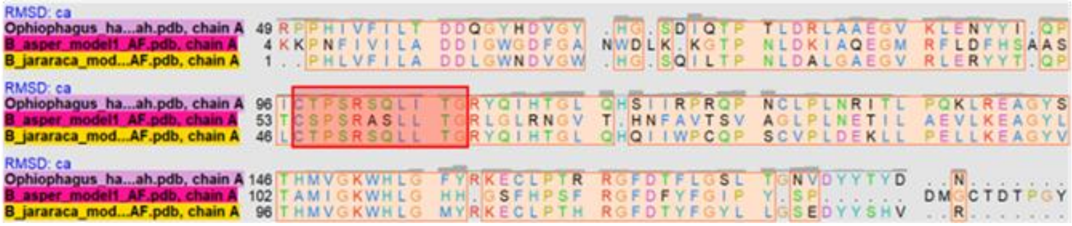

FS6. Sulfatase motif of Arylsulfatase. Alignment of the arylsulfatase patterns of A) *Bothrops asper*, B) *Bothrops jararaca* and C) *Ophiophagus hannah*, made with CHIMERA. D) The region marked in red highlights the conserved motif C/S-X-P-X-R-X4-TG.

3.2. Structural analysis of calcium-binding arylsulfatase residues in Bothrops asper

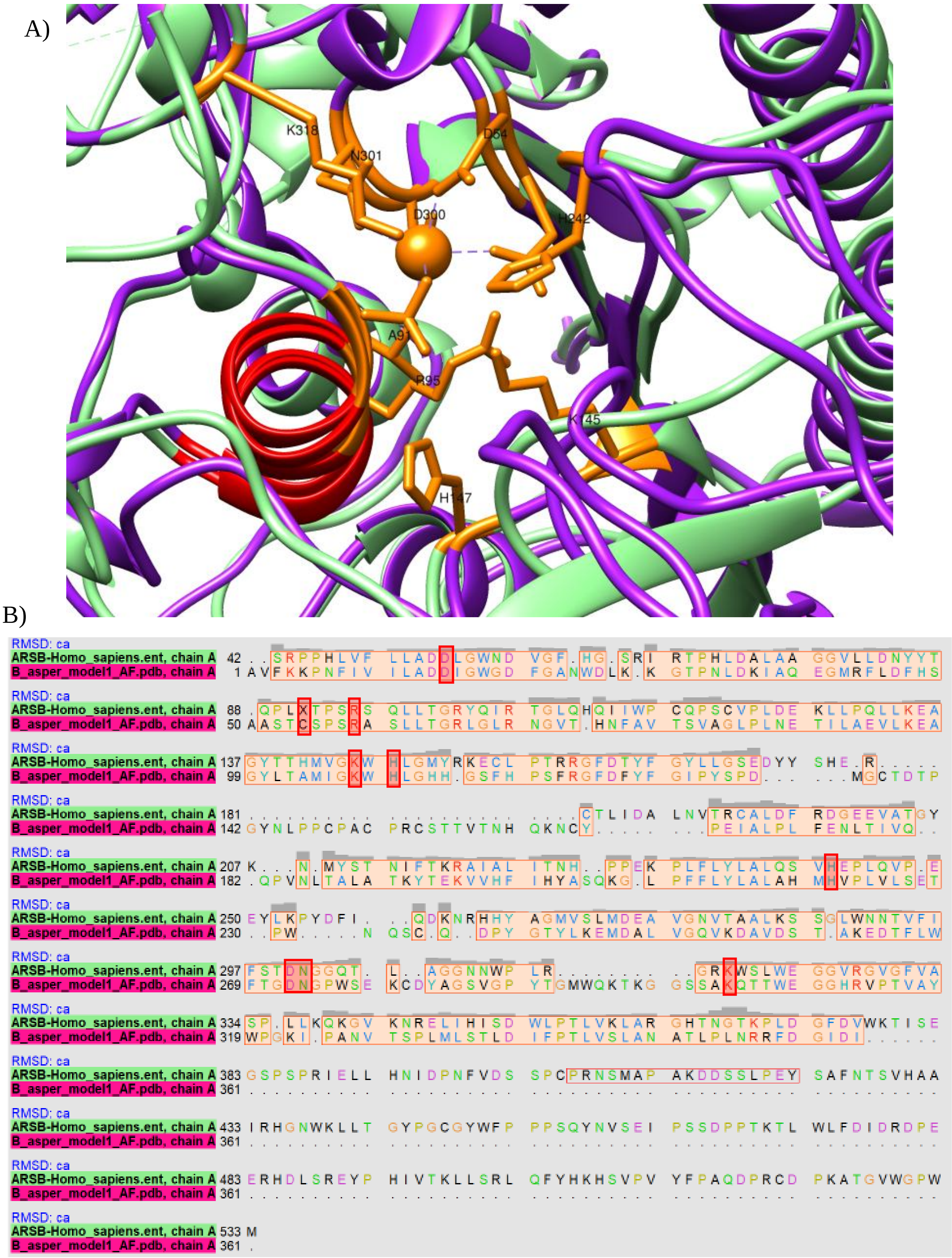

FS7. Calcium-binding arylsulfatase residues. A) Comparison of calcium-binding sites between *Bothrops asper* (purple) and *Homo sapiens* (green). B) Alignment of the arylsulfatase models from *Bothrops asper* and *Homo sapiens* [PDB ID: 1FSU] generated with CHIMERA. Residues Asp54, Cys91, Arg95, Lys145, His147, His242, Asp300, Asn301, and Lys318, marked in red, interact with calcium ions.

4. Botrocetin

4.1. Structural analysis

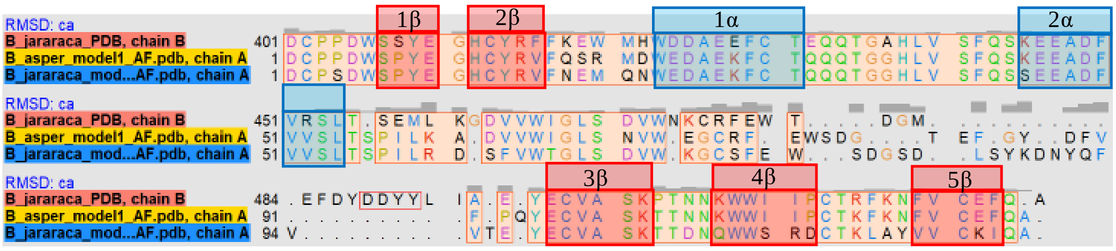

FS8. Conservation of residues involved in the formation of alpha helices (red) and beta folds (blue) for *Bothrops asper* and *Bothrops jararaca*, compared to botrocetin of PDB (B_jararaca_PDB). Alignment generated with CHIMERA.

5. Dihydroorotate dehydrogenase

5.1. Multiple alignment analysis for Bothrops asper and Bothrops jararaca

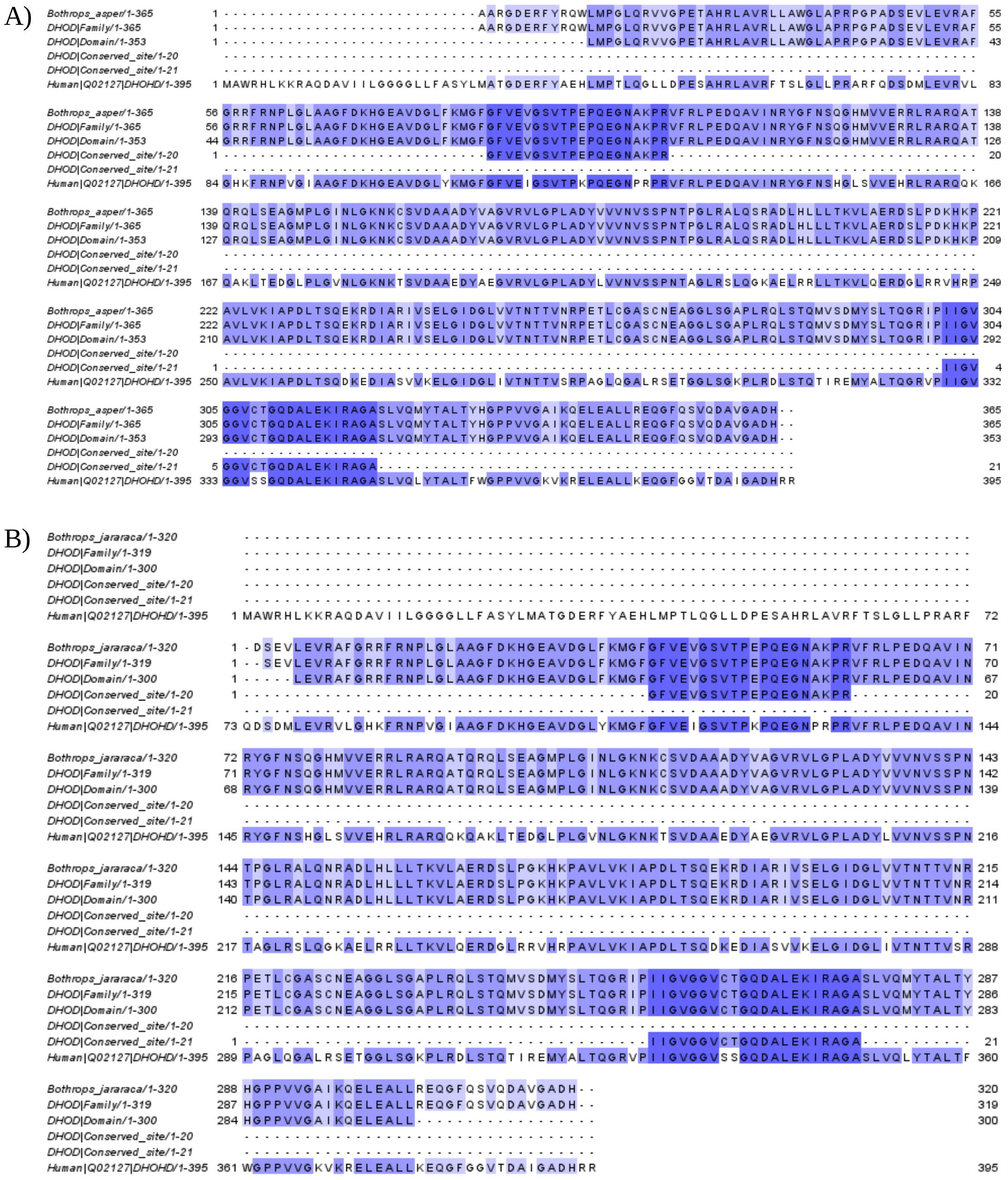

FS9. Multiple alignment of PFAM results for dihydroorotate dehydrogenase. A) Annotated sequences for *Bothrops asper*. B) Annotated sequences for *Bothrops jararaca*. Identification of the domain, family, and conserved sites of dihydroorotate dehydrogenase (DHODH). High similarity to human DHODH [PDB 3U2O].

5.2. Structural analysis for conserved residues

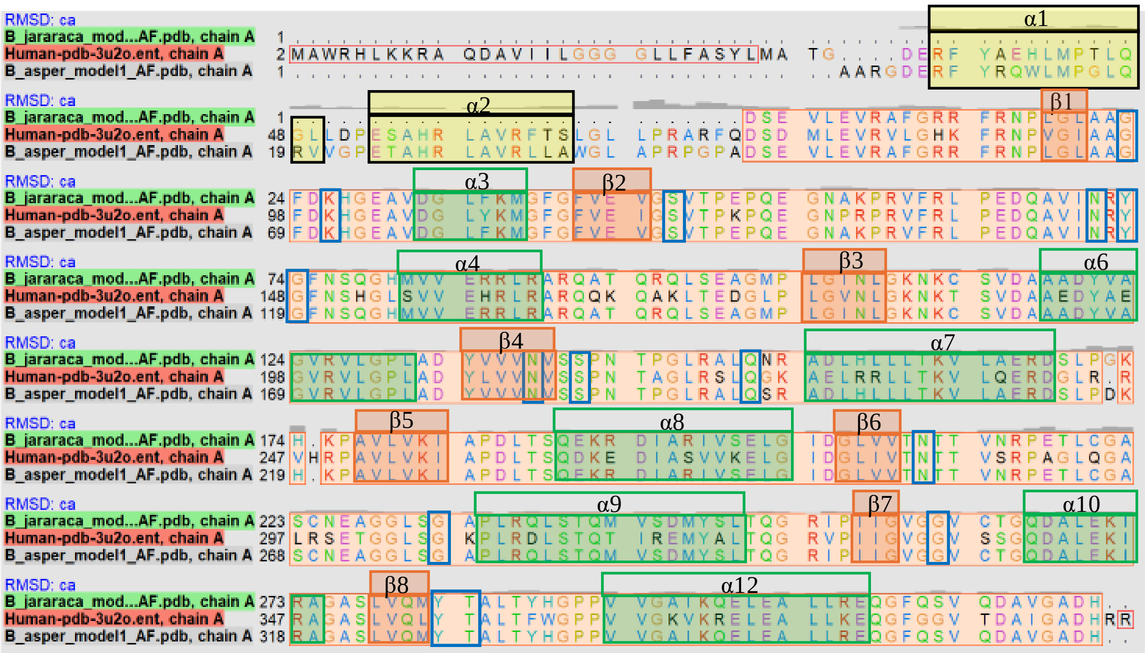

FS10. Conserved residues in dihydroorotate dehydrogenase (DHODH) between *Bothrops asper*, *Bothrops jararaca*, and *Homo sapiens* [PDB 3U2O]. Blue for residues involved in DHODH function, yellow for residues in the small domain (α1 and α2), green for residues in the large domain (α3, α4, α6–α10, and α12), and orange for residues in the large domain (β1–β8). Alignment generated with CHIMERA.

6. Basparin

6.1. Multiple alignment analysis of Bothrops asper

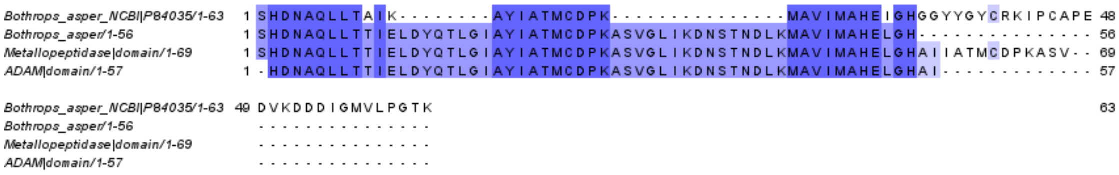

FS11. Alignment of PFAM results for basparin. Identification of the reprolysin-like metalloproteinase (metallopeptidase) domain and the ADAM domain. Comparison between the sequence obtained (Ba_1) and basparin from *Bothrops asper* [P84035].

6.2. Structural analysis of residues involved in zinc binding

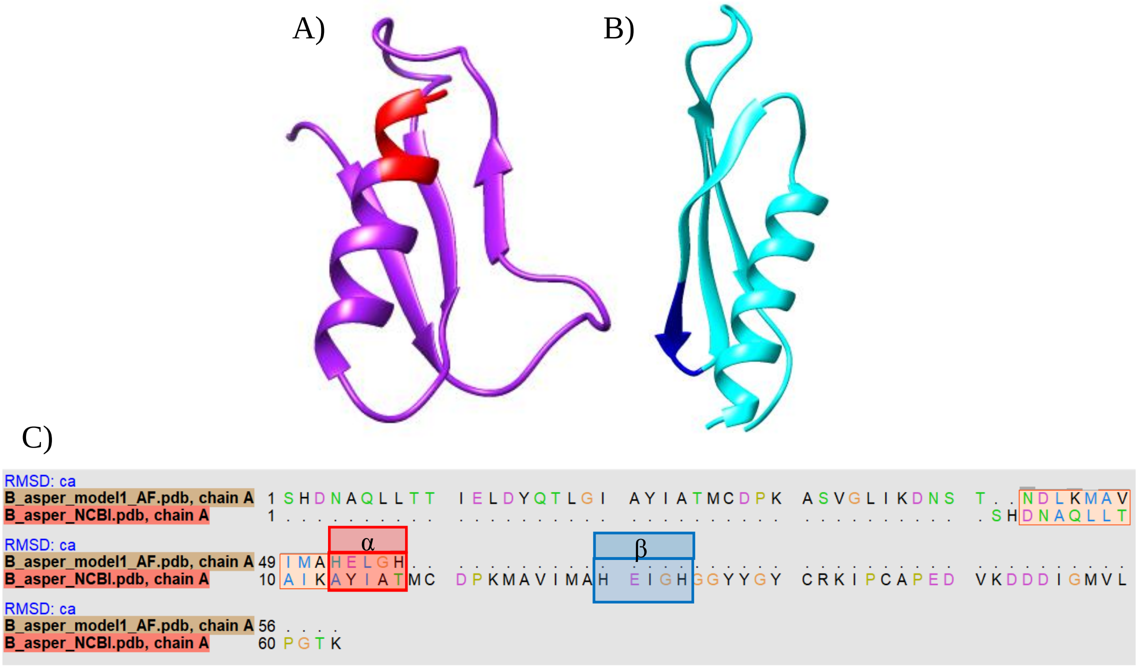

FS12. Conserved residues involved in zinc binding. Basparin models for A) basparin from the UniProt database [P84035] and B) the model generated in this study for *Bothrops asper*, designated Ba_1. C) Structural alignment of the CHIMERA models: the beta fold (blue) for basparin and the alpha helix (red) for Ba_1. The sequences and models indicate the zinc-binding site (HEXXH).

7. Bothropasin

7.1. Multiple alignment analysis of Bothrops asper

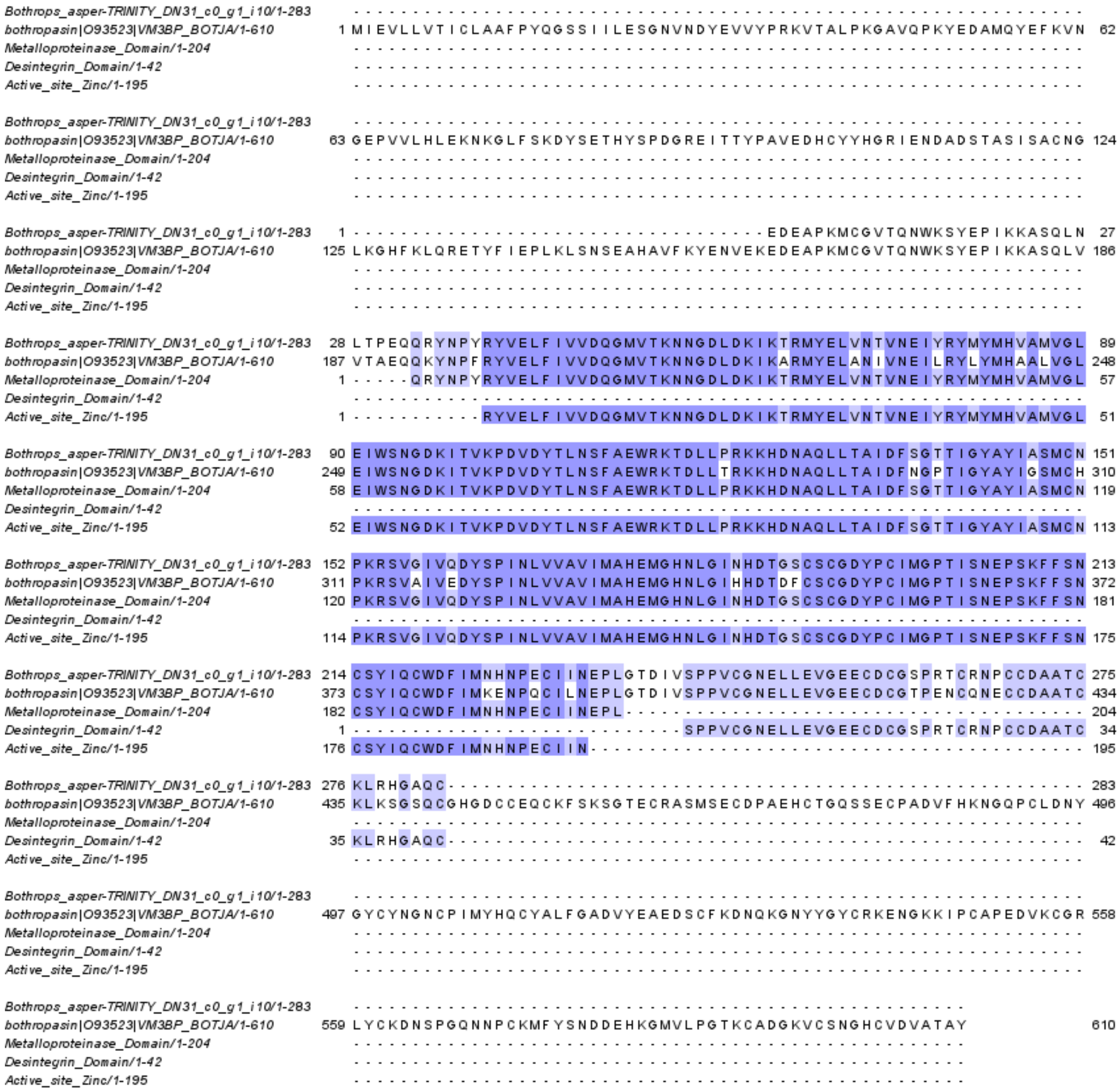

FS13. Alignment of PFAM results for bothropasin. Identification of the metalloproteinase domain, ADAM domain, and the active site for zinc binding.

7.2. Structural analysis of conserved domains

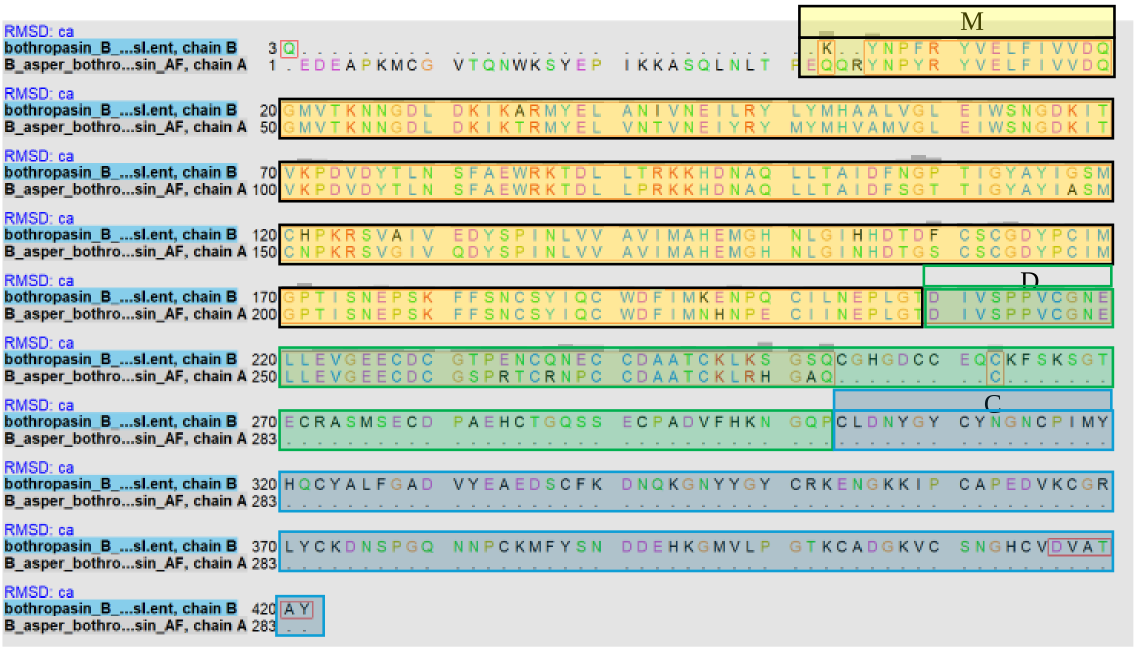

FS14. Identification of SVMP-PII domains between the model obtained from B_asper_bothropasin_AF and the bothropasin from *Bothrops jararaca* [O93523]. The metallorpoteinase domain (M) is marked in yellow, the disintegrin domain (D) in green, and the cysteine-rich domain (C) in blue. Structural alignment of the models generated with CHIMERA.

7.3. Structural analysis of zinc-binding residues

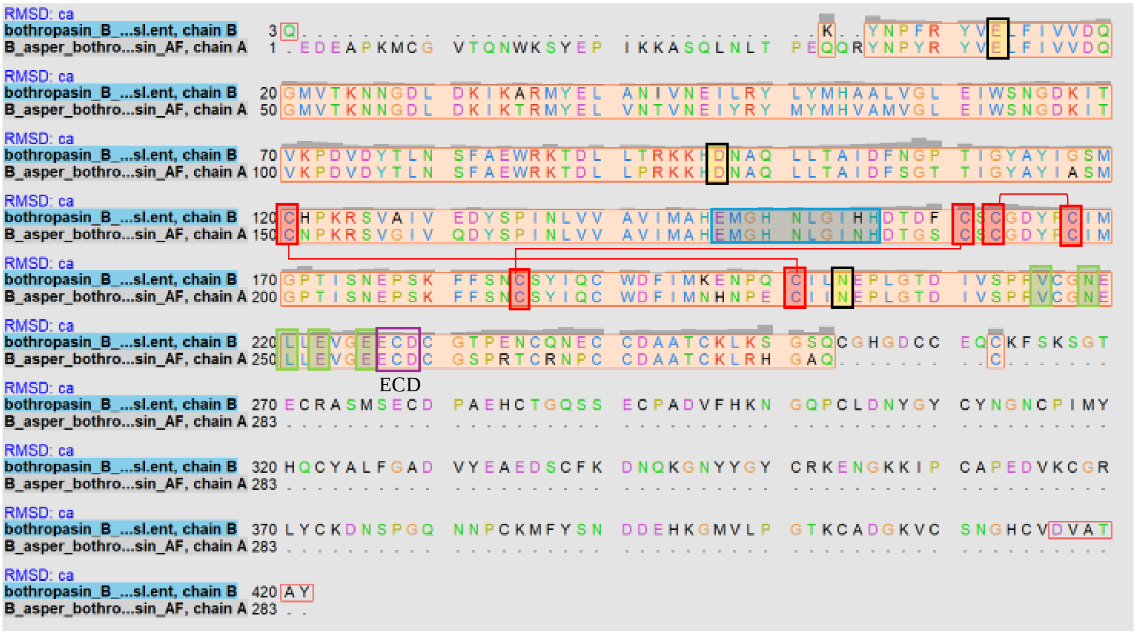

FS15. Conservation of key residues, with zinc binding in blue (HEXXHXXGXXH) and cysteine residues forming three disulfide bonds in red. Calcium-binding residues are colored yellow for the M domain and green for the D domain. Identification of the disintegrin loop (ECD). Structural alignment between B_asper_bothropasin_AF and *Bothrops jararaca* bothropasin, generated with CHIMERA.

A)

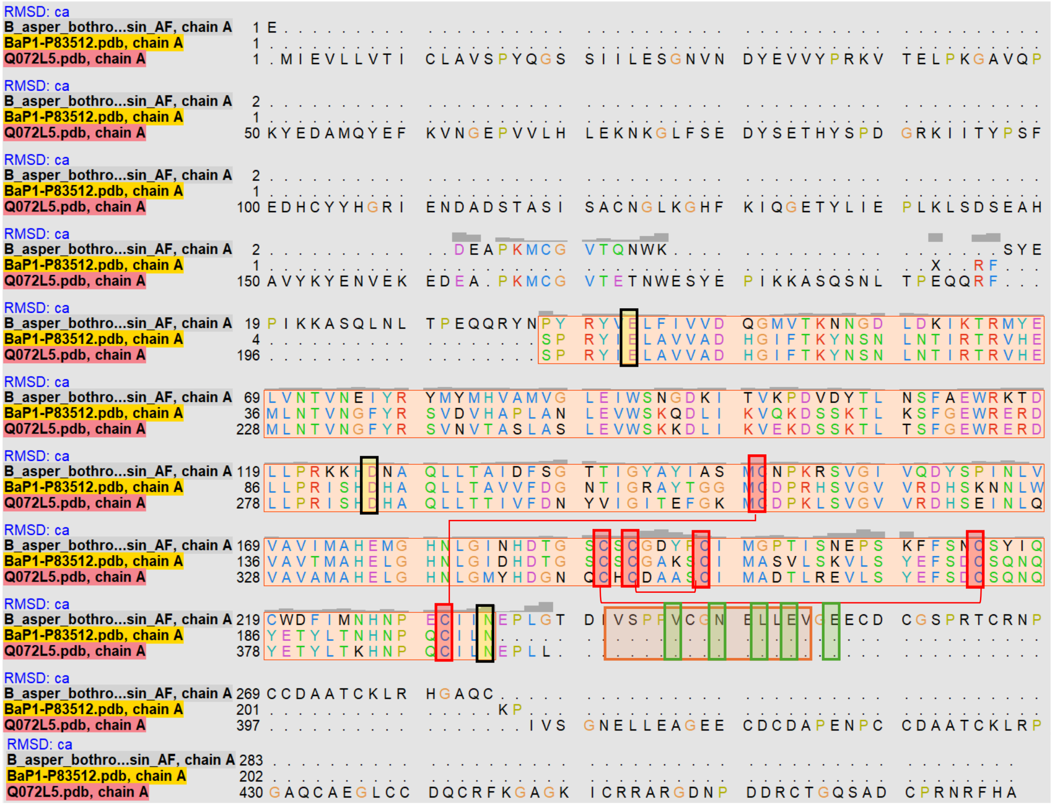

B)

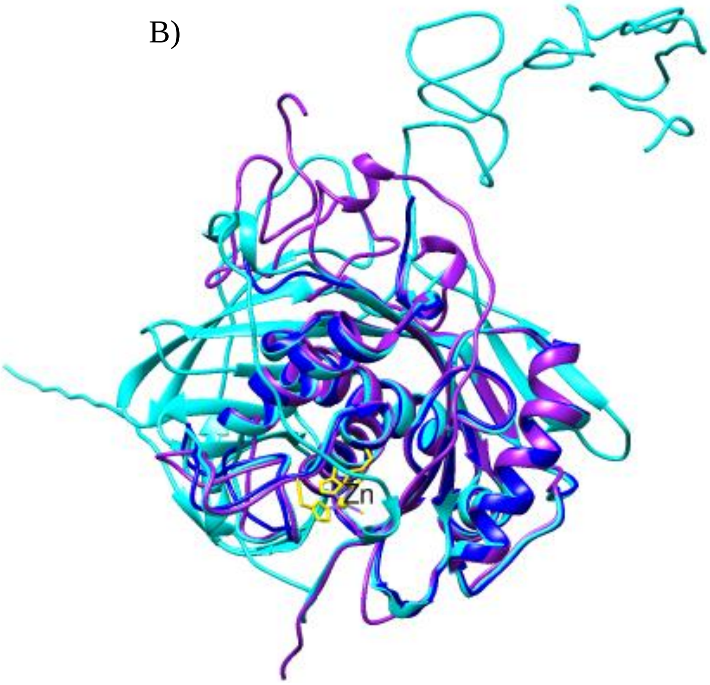

FS16. A) Structural alignment of models generated with CHIMERA. Cysteine residues that form disulfide bonds in red, calcium-binding residues that are part of the M domain are shown in yellow, and the D domain in green. Disintegrin-binding region is shown in orange. B) Comparison of the models of the disintegrins bothrasperin (Q072L5) and BaP1 (P83512) from *Bothrops asper* with the obtained B_asper_bothropasin_AF model. Model in purple for B_asper_bothropasin_AF, in blue P83512, and in light blue Q072L5.

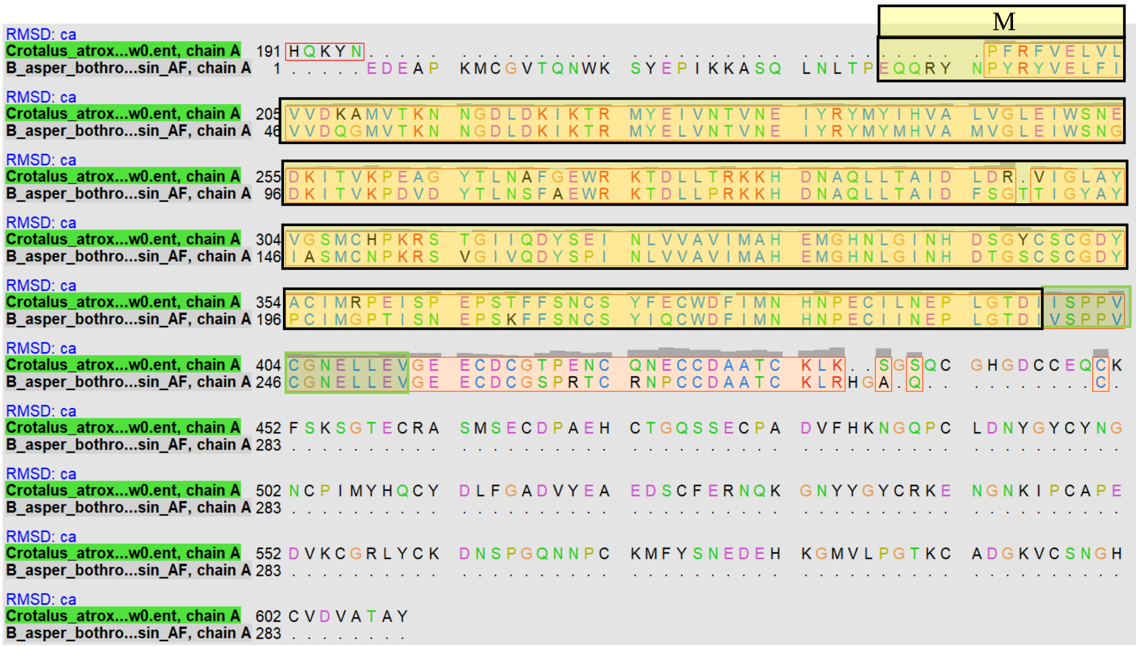

FS17. Structural comparison of VAP2B from *Crotalus atrox* [Q90282] with the derived model B_asper_bothropasin_AF. The metalloproteinase domain (yellow) and disintegrin-binding region (green) are preserved. Structural alignment generated with CHIMERA.
