# Supplementary material for: Computational Modeling of Low-Abundance Proteins in Venom Gland Transcriptomes: Bothrops asper and Bothrops jararaca
Source: Toxins (Basel). 2025 May 22;17(6):262. doi: 10.3390/toxins17060262 (PMC12197698; doi:10.3390/toxins17060262)
Supplement: Supplementary file 1 [file toxins-17-00262-s001.zip › Supplementary Material 2/Supplementary Materials 2.pdf]

Computational Modeling of Low-Abundance Proteins in Venom Gland Transcriptomes: *Bothrops asper* and *Bothrops jararaca*

Joseph Espín-Angulo and Doris Vela*

Supplementary Material 2: Validation Parameters

1. CRISP

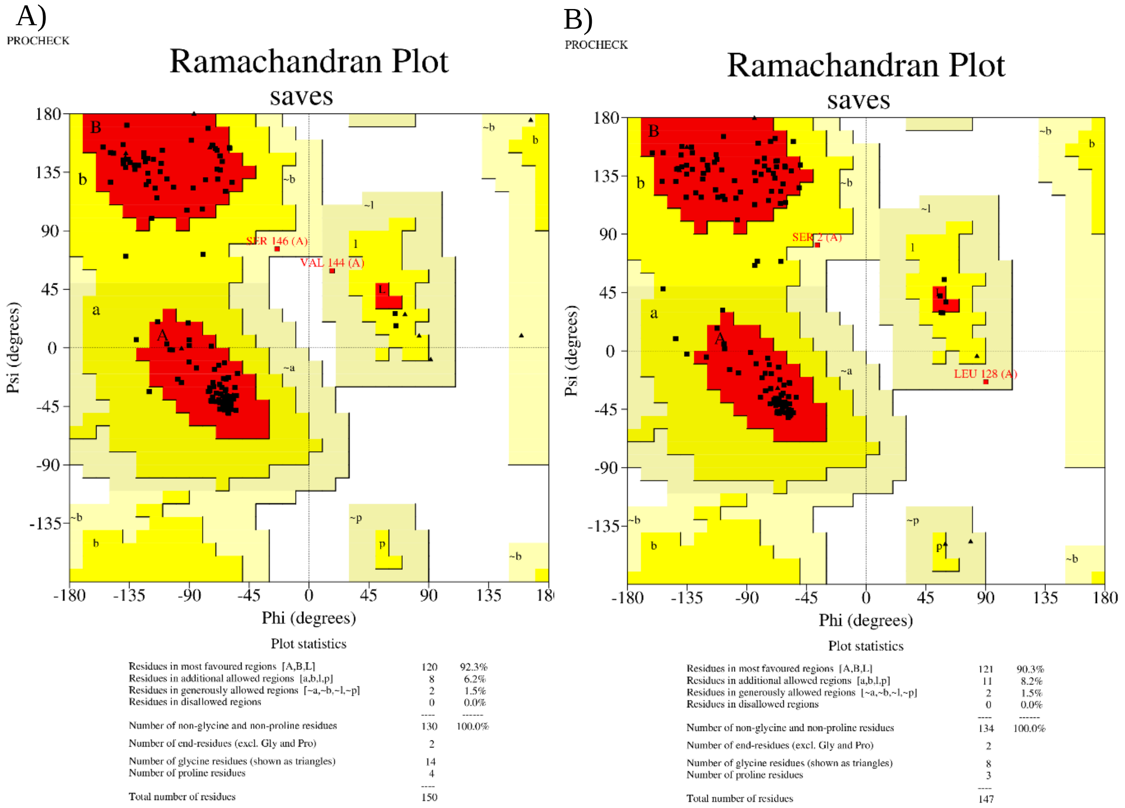

FS1.1. Ramachandran plots. Structural validation of CRISP models of (A) *Bothrops asper* and (B) *Bothrops jararaca*.

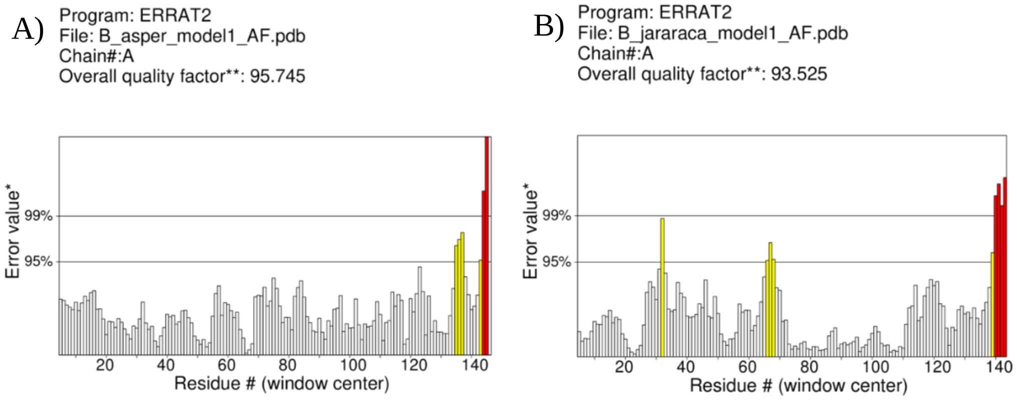

FS1.2. ERRAT. Structural validation of CRISP models of (A) *Bothrops asper* and (B) *Bothrops jararaca*.

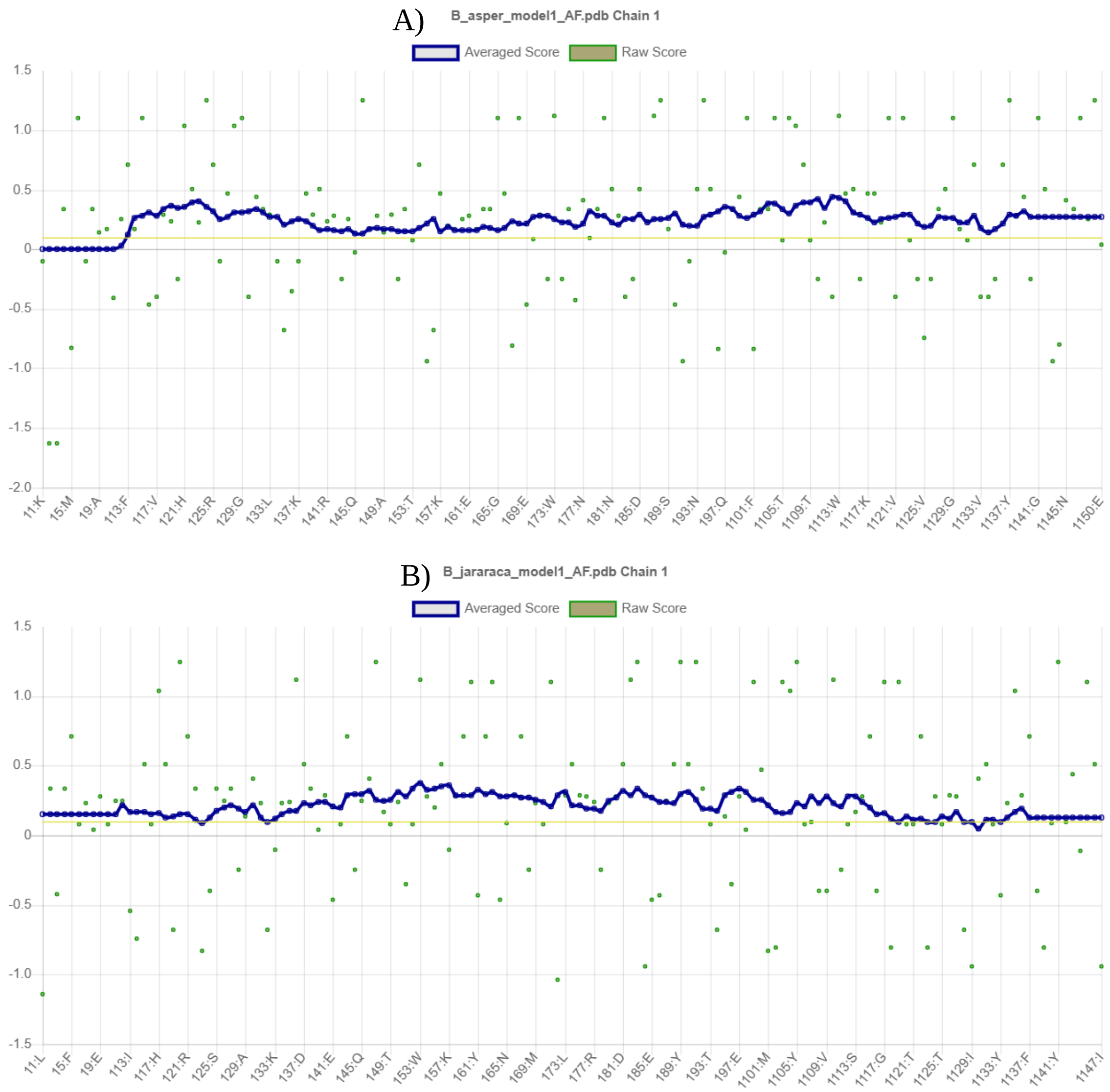

FS1.3. Verify3D. Structural validation of CRISP models of (A) *Bothrops asper* and (B) *Bothrops jararaca*.

2. Von Willebrand Factor

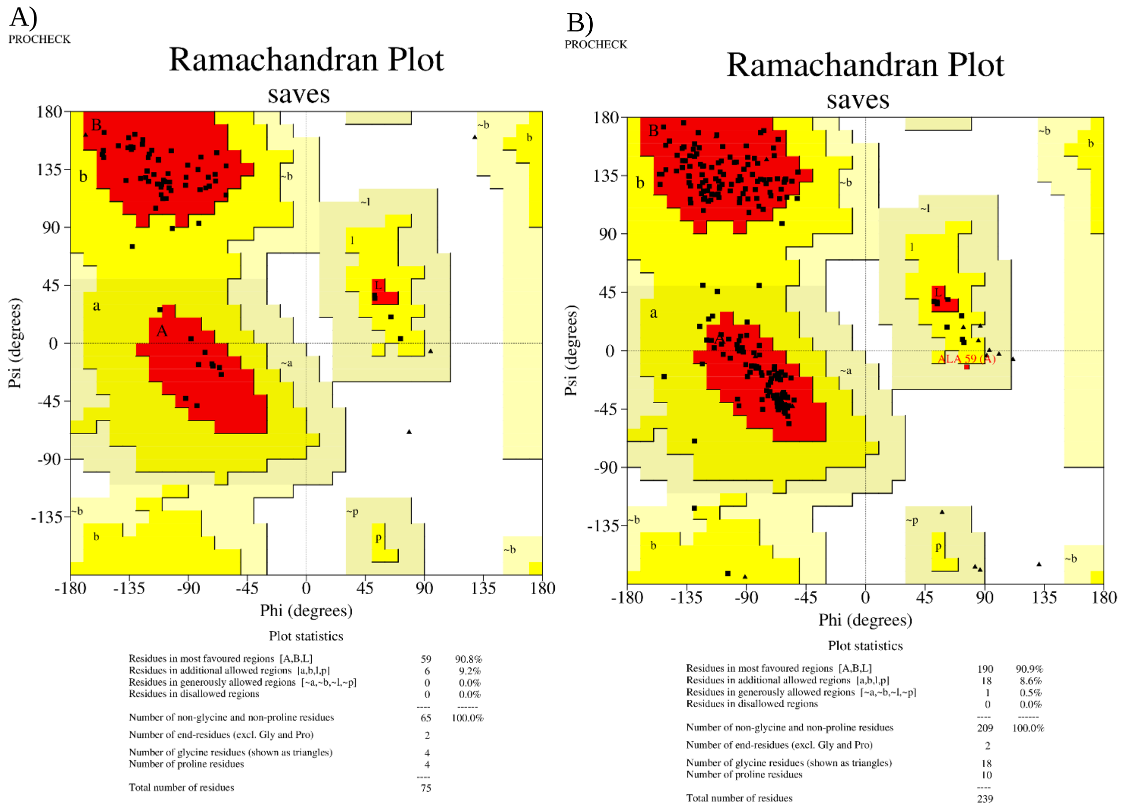

FS2.1. Ramachandran plots. Structural validation of von Willebrand Factor models of (A) *Bothrops asper* and (B) *Bothrops jararaca*.

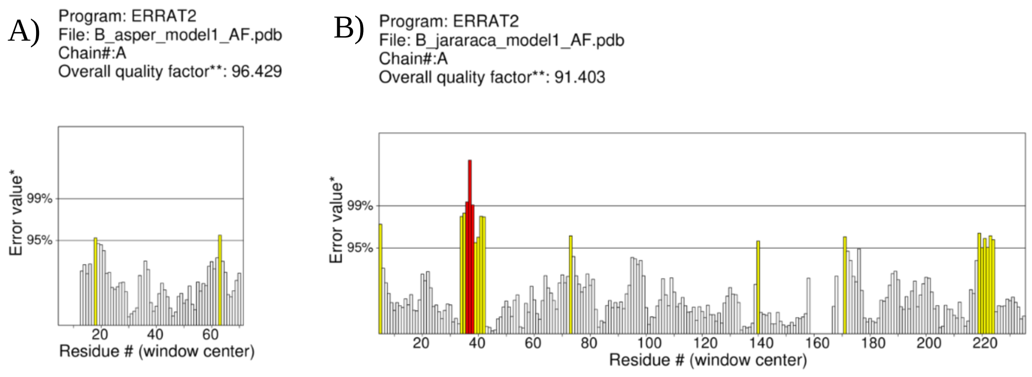

FS2.2. ERRAT. Structural validation of von Willebrand Factor models of (A) *Bothrops asper* and (B) *Bothrops jararaca*.

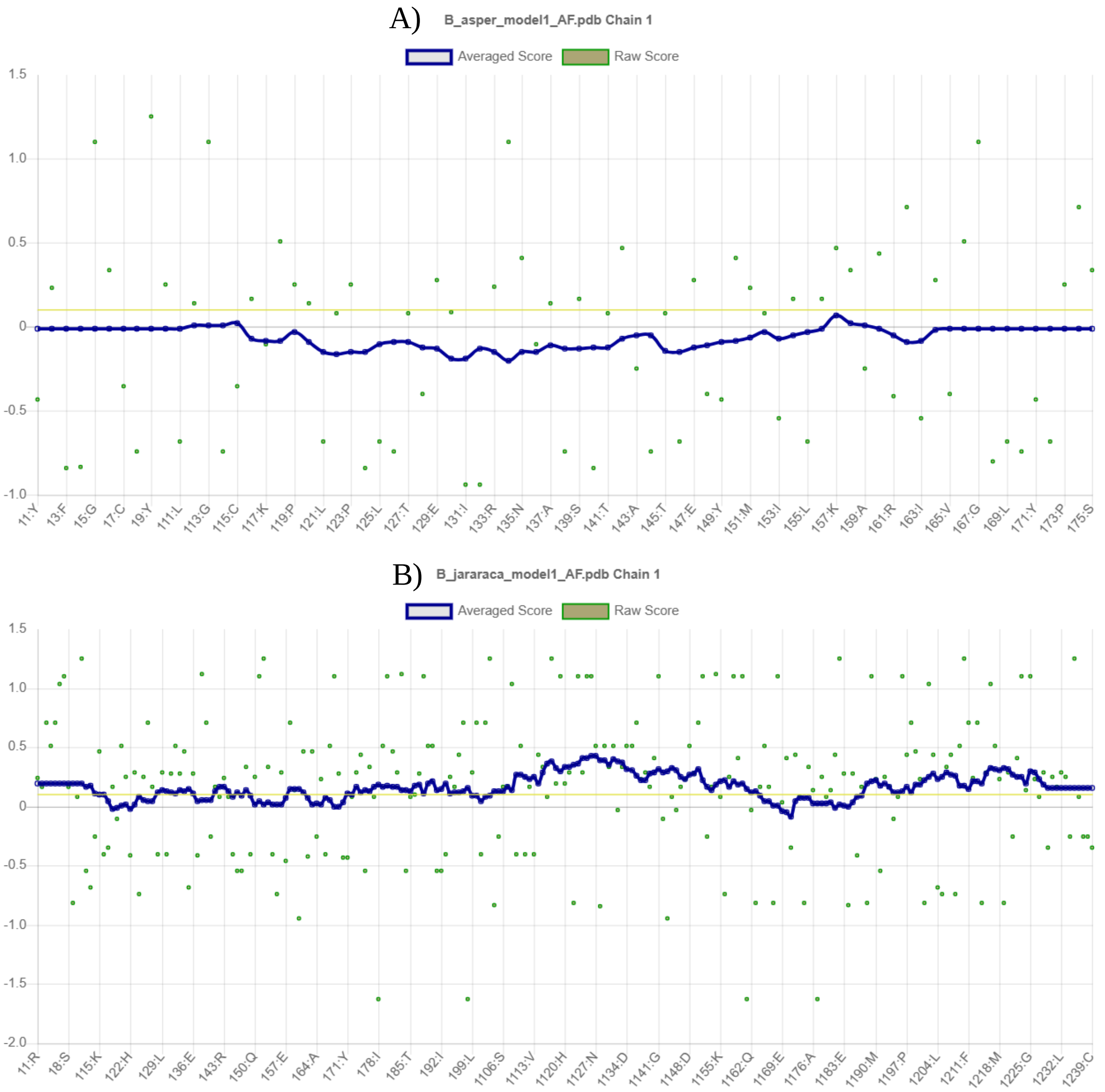

FS2.3. Verify3D. Structural validation of von Willebrand Factor models of (A) *Bothrops asper* and (B) *Bothrops jararaca*.

3. Von Willebrand Factor type D

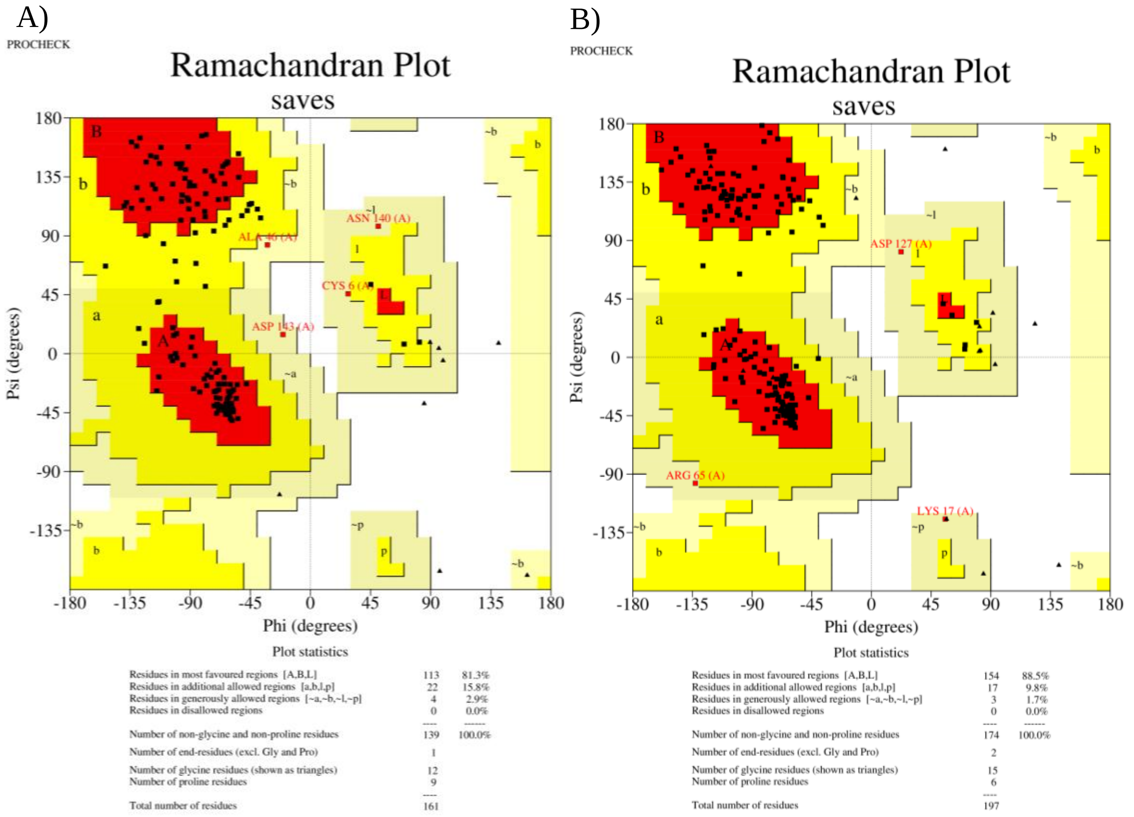

FS2.1.1. Ramachandran plots. Structural validation of von Willebrand Factor type D models of (A) *Bothrops asper* and (B) *Bothrops jararaca*.

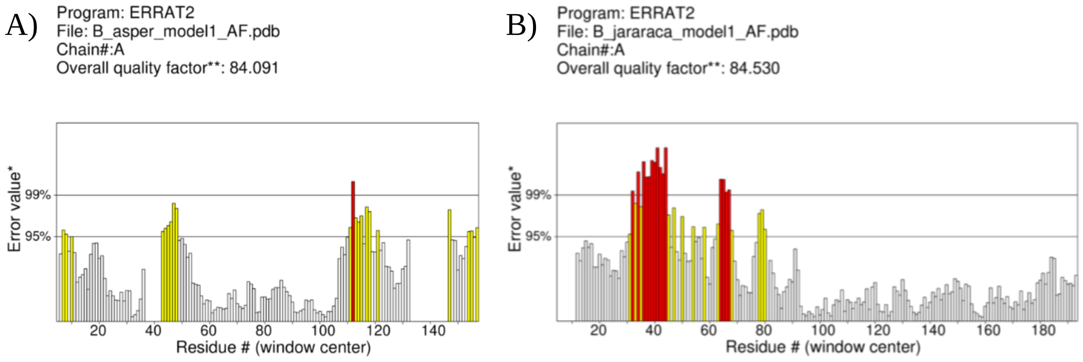

FS2.1.2. ERRAT. Structural validation of von Willebrand Factor type D models of (A) *Bothrops asper* and (B) *Bothrops jararaca*.

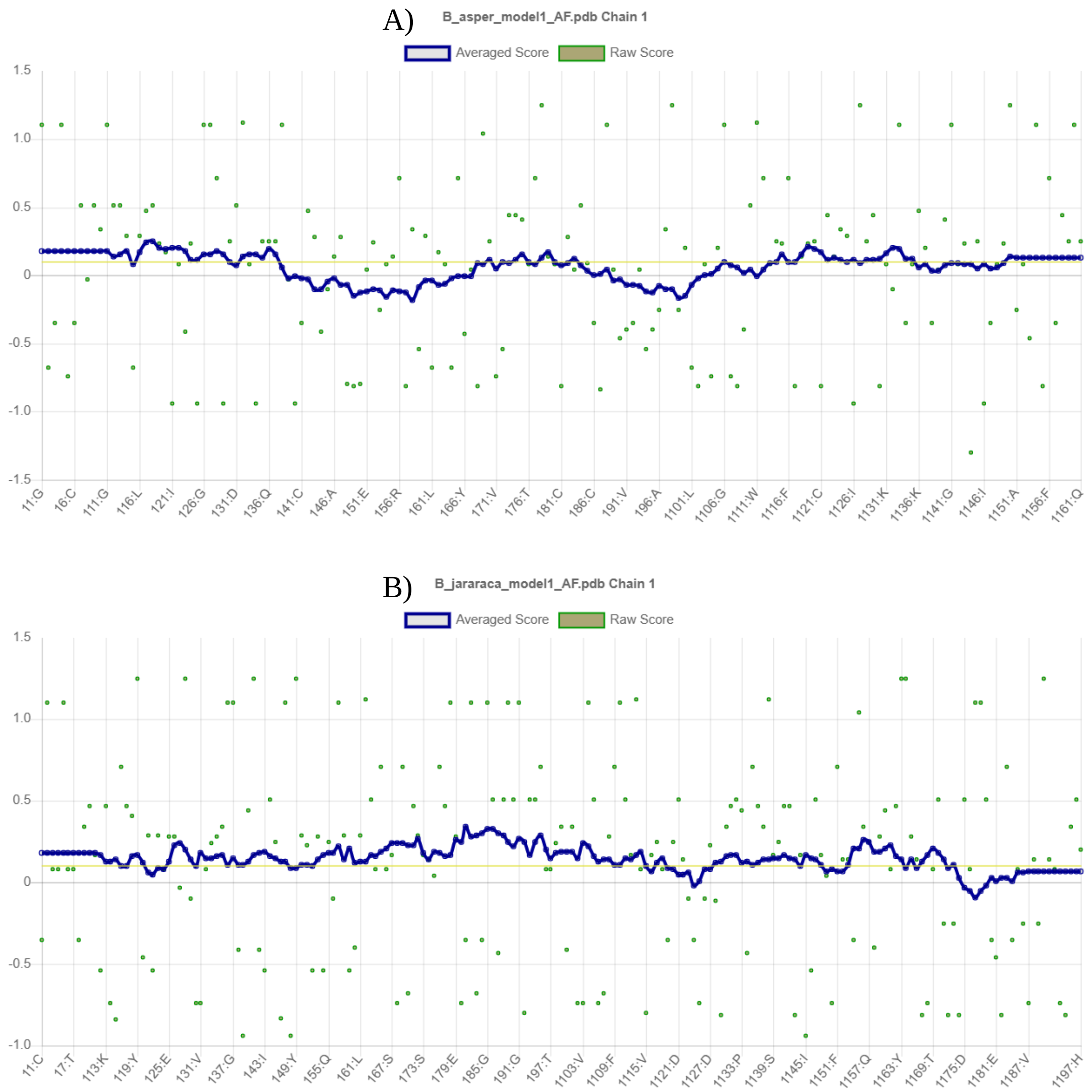

FS2.1.3. Verify3D. Structural validation of von Willebrand Factor type D models of (A) *Bothrops asper* and (B) *Bothrops jararaca*.

4. Arylsulfatase

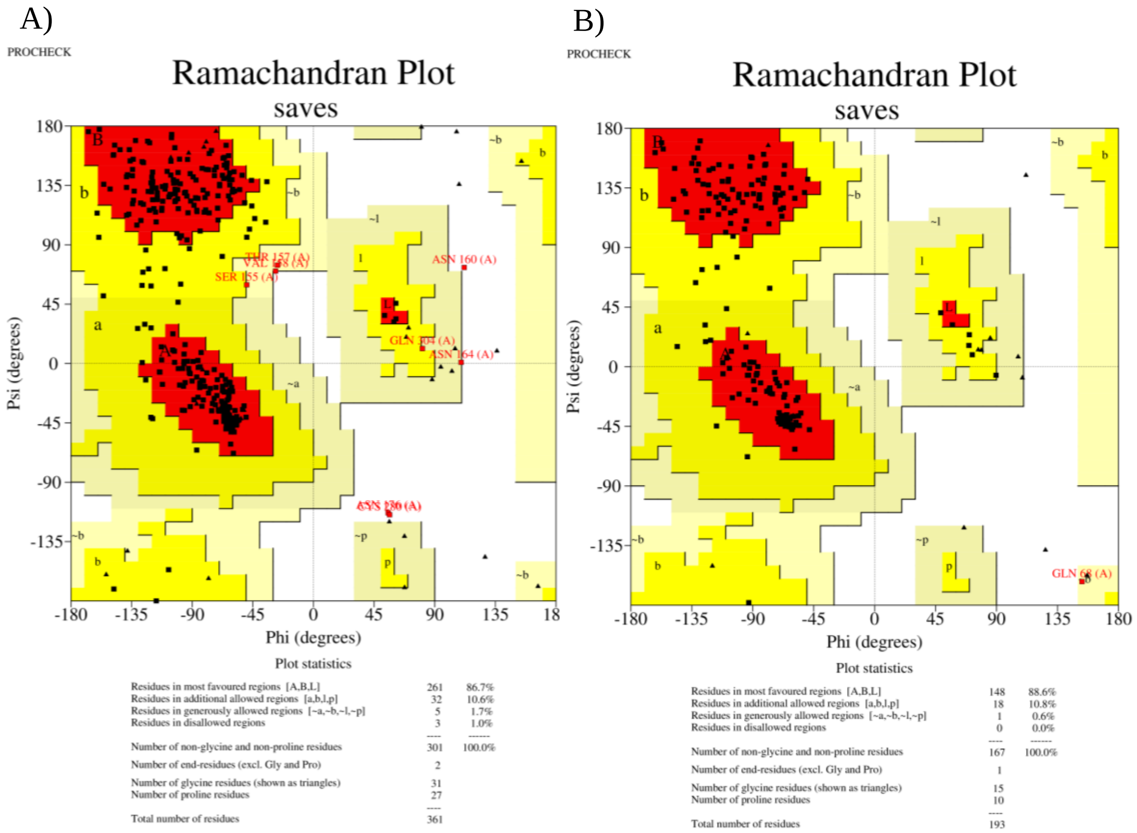

FS3.1. Ramachandran plots. Structural validation of arylsulfatase models of (A) *Bothrops asper* and (B) *Bothrops jararaca*.

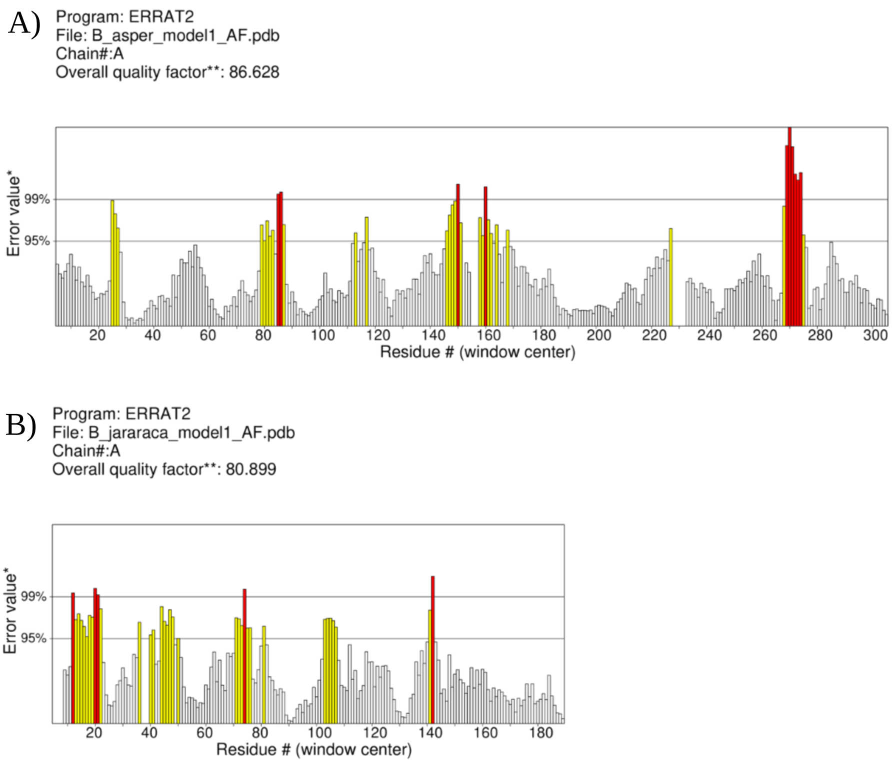

FS3.2. ERRAT. Structural validation of arylsulfatase models of (A) *Bothrops asper* and (B) *Bothrops jararaca*.

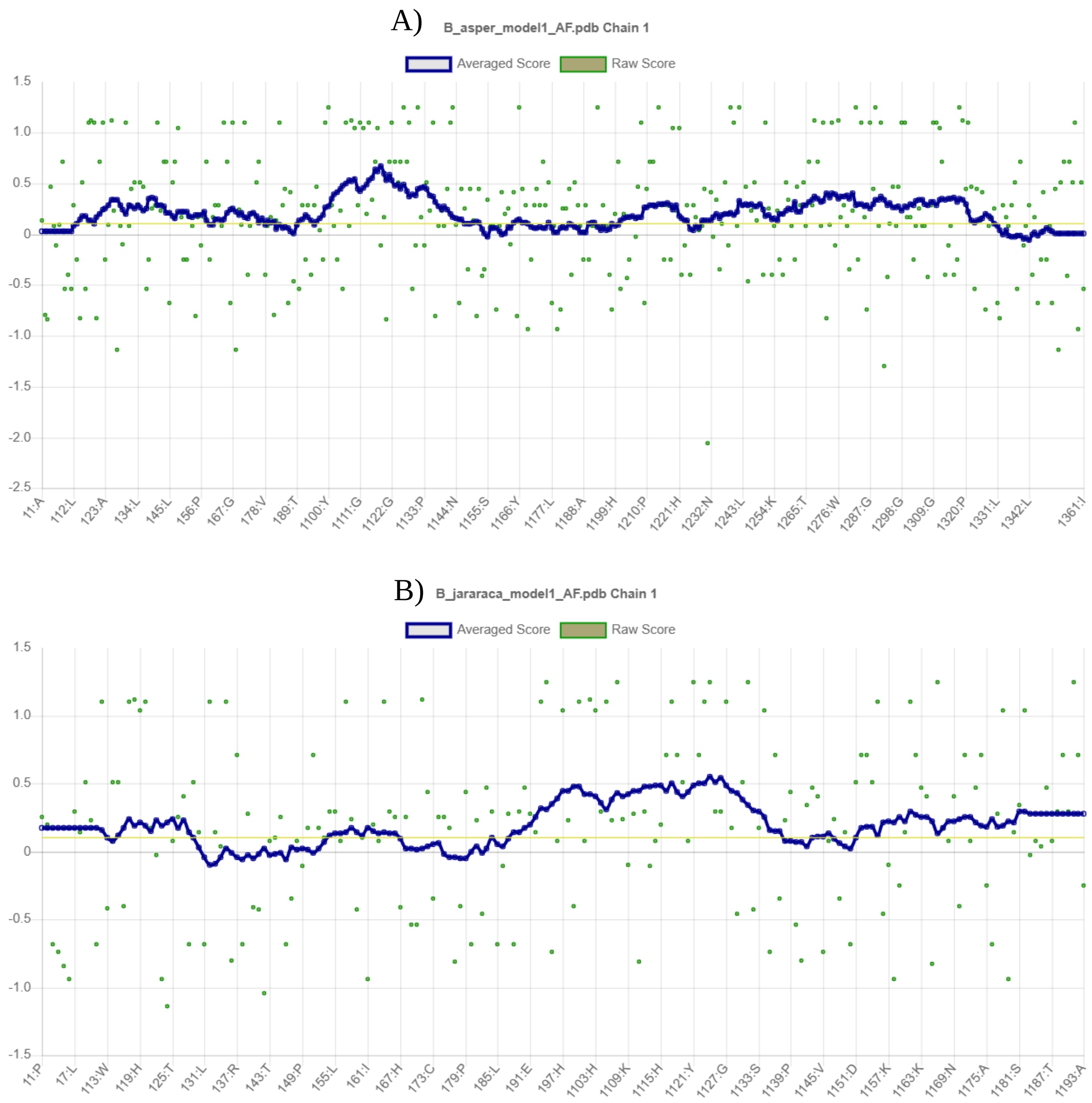

FS3.3. Verify3D. Structural validation of arylsulfatase models of (A) *Bothrops asper* and (B) *Bothrops jararaca*.

5. Botrocetin

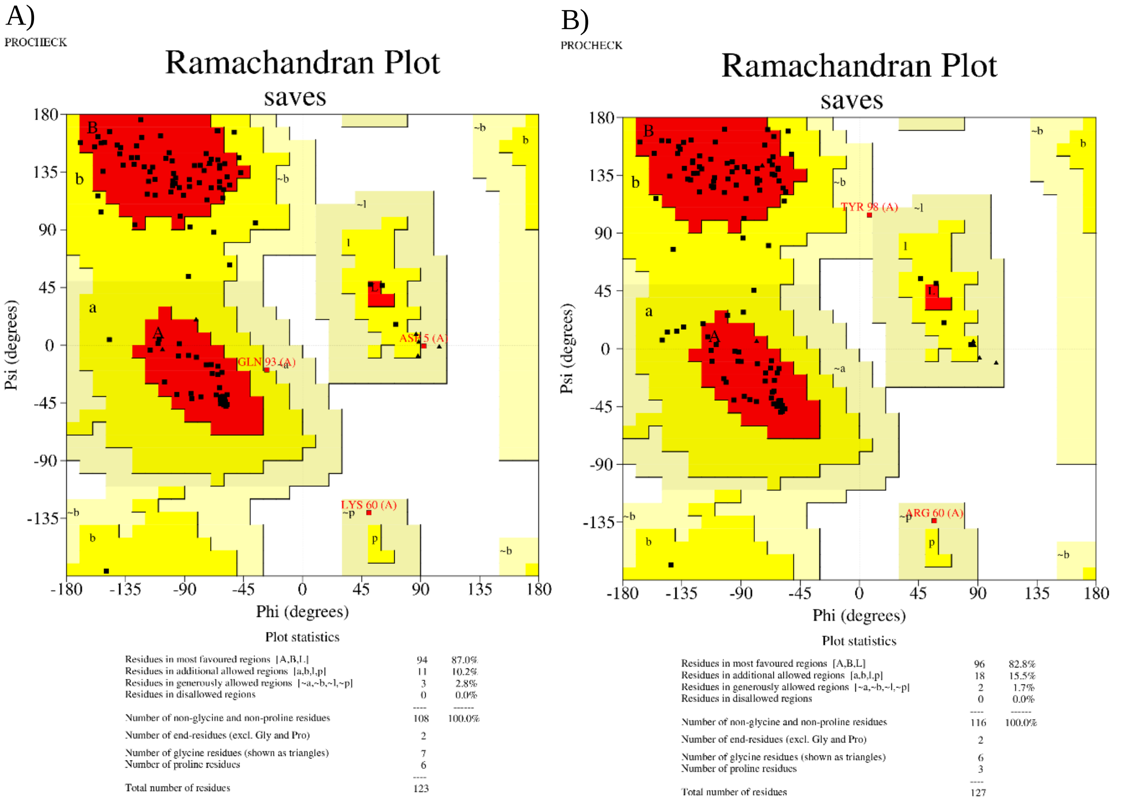

FS4.1. Ramachandran plots. Structural validation of botrocetin models of (A) *Bothrops asper* and (B) *Bothrops jararaca*.

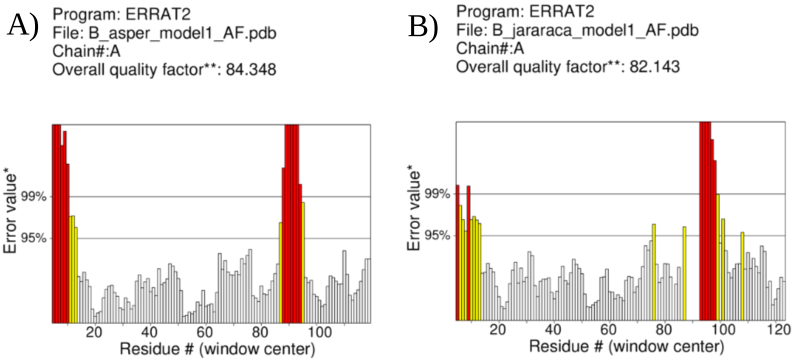

FS4.2. ERRAT. Structural validation of botrocetin models of (A) *Bothrops asper* and (B) *Bothrops jararaca*.

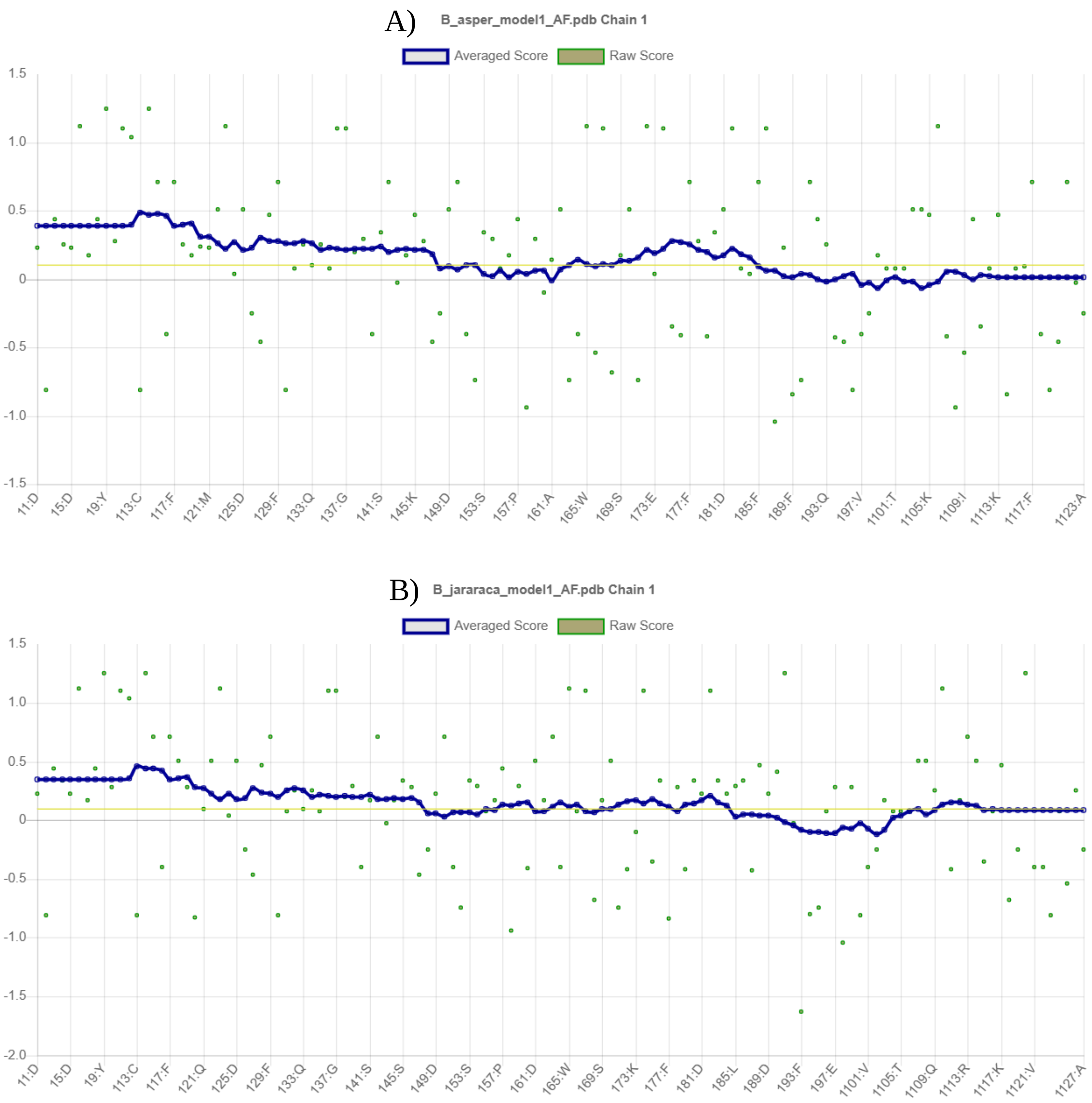

FS4.3. Verify3D. Structural validation of botrocetin models of (A) *Bothrops asper* and (B) *Bothrops jararaca*.

6. Dihydroorotate dehydrogenase

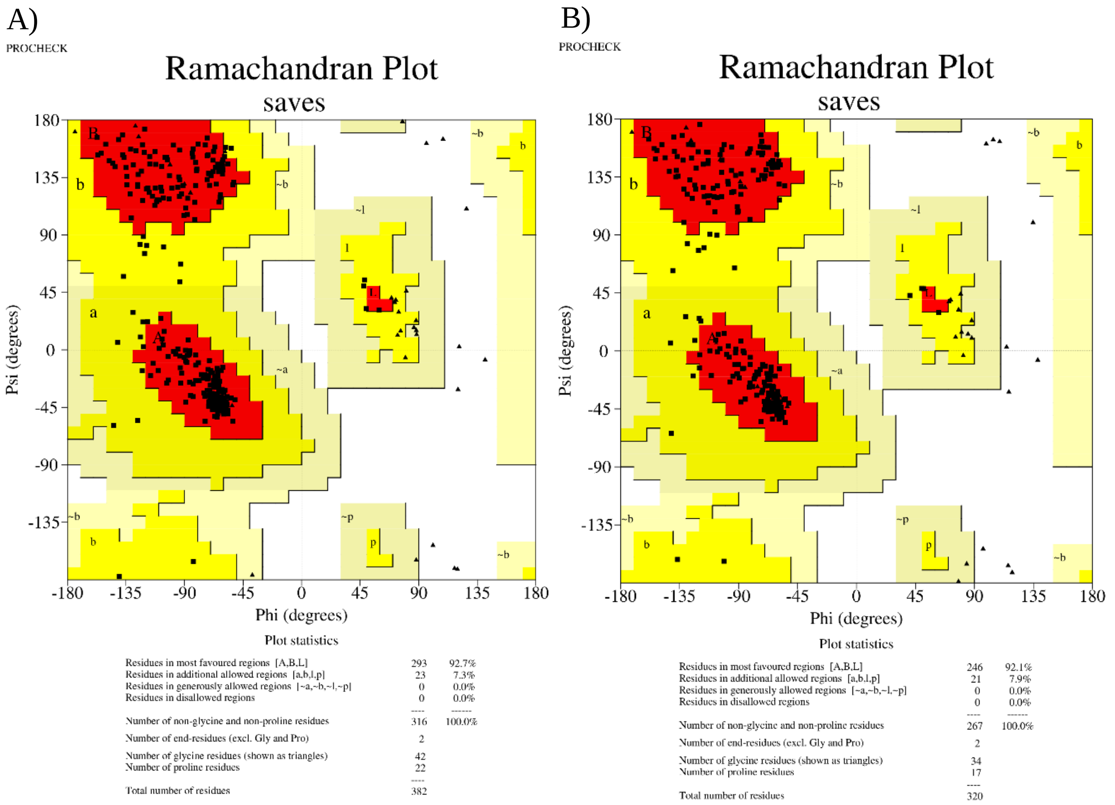

FS5.1. Ramachandran plots. Structural validation of Dihydroorotate dehydrogenase models of (A) *Bothrops asper* and (B) *Bothrops jararaca*.

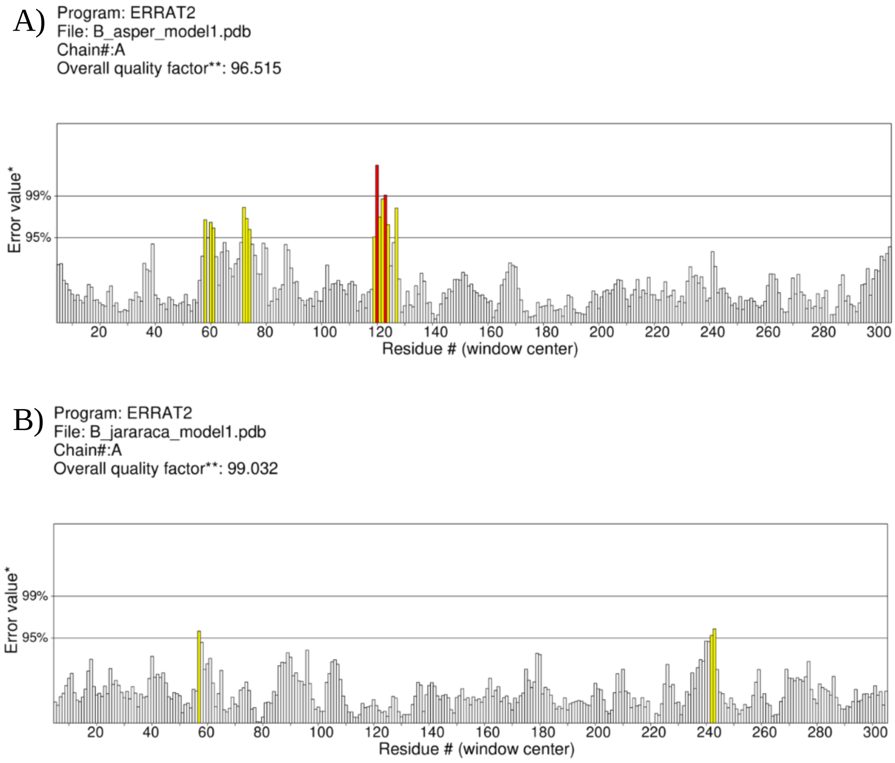

FS5.2. ERRAT. Structural validation of Dihydroorotate dehydrogenase models of (A) *Bothrops asper* and (B) *Bothrops jararaca*.

A)

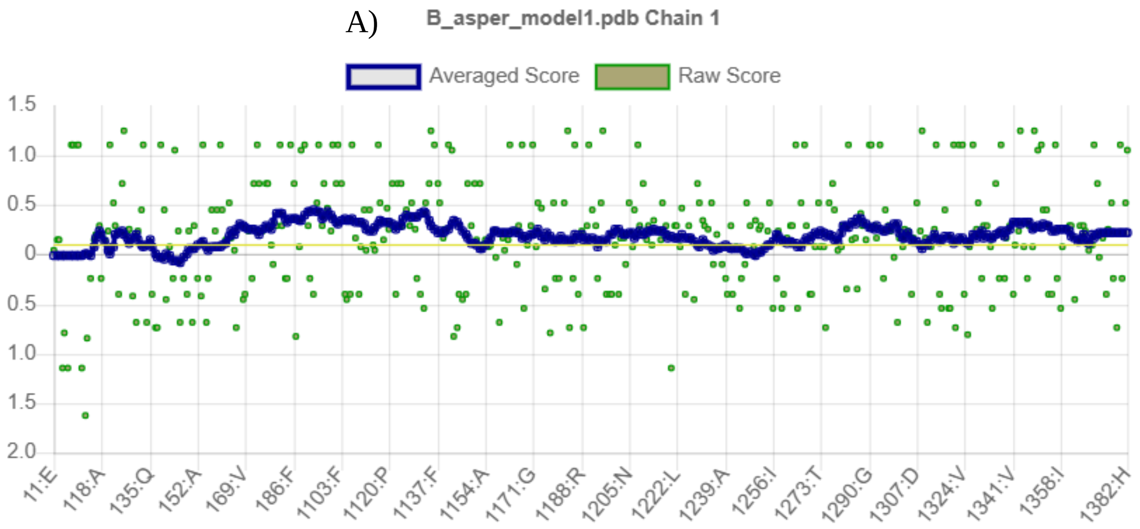

B)

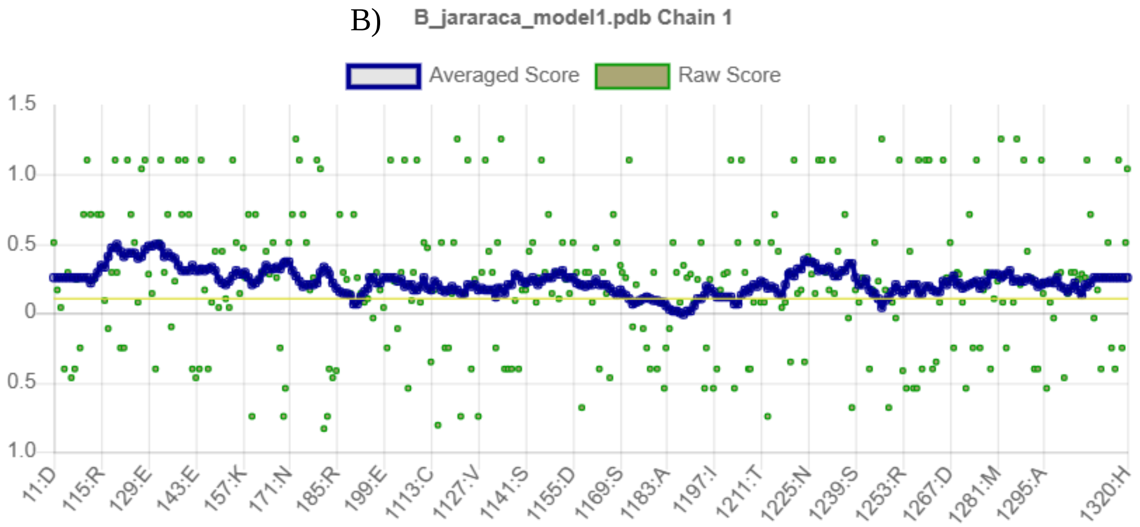

FS5.3. Verify3D. Structural validation of Dihydroorotate dehydrogenase models of (A) *Bothrops asper* and (B) *Bothrops jararaca*.

7. Basparin

This section compares two structural models of the *Bothrops asper* Basparin protein: one predicted from the transcriptome sequence obtained in this study, and another available in the UniProt database (P84035). Although both models are based on the same protein, they present structural differences that are reflected in the validation scores. In particular, the model predicted in this study obtained a low score in Verify3D (1.79%), while the reference model in UniProt achieved a moderate value (55.56%). However, the latter also presents flexible regions with low scores, suggesting that the observed limitations are associated with the intrinsically flexible nature of the protein and not necessarily with errors in the modeling.

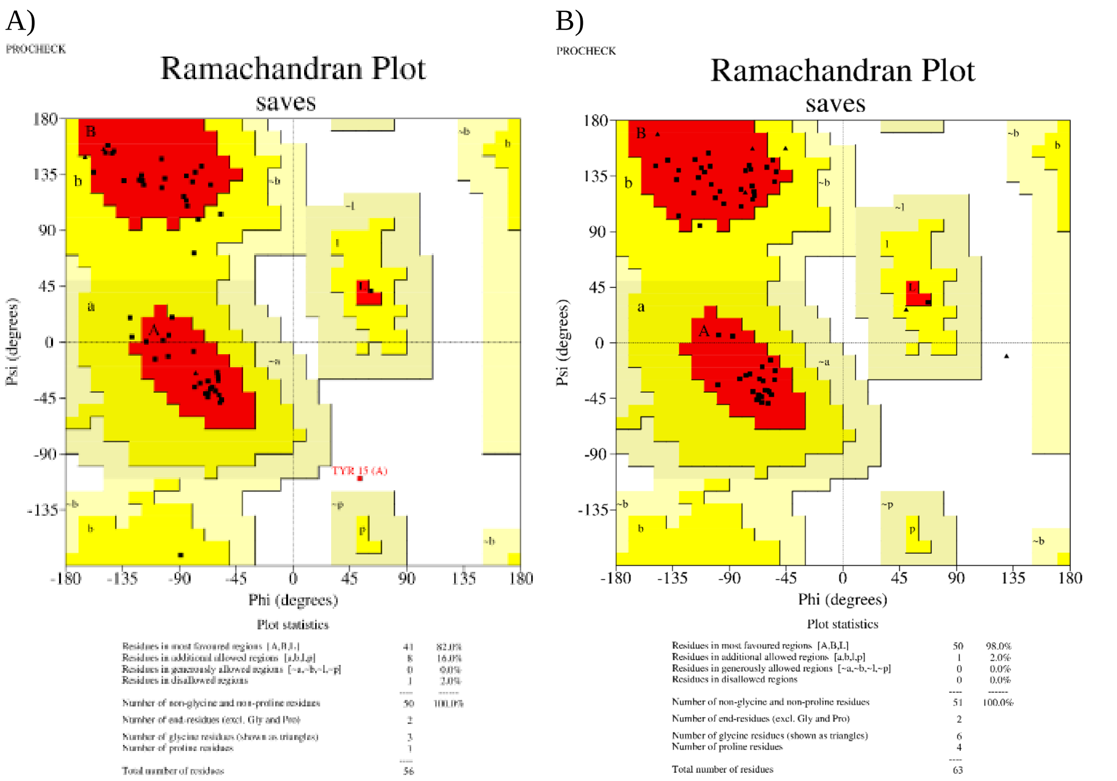

FS6.1. Ramachandran plots. A) Structural validation of the predicted model for Basaprin from *Bothrops asper*. B) Structural validation of Basaprin from UniProt database [P84035].

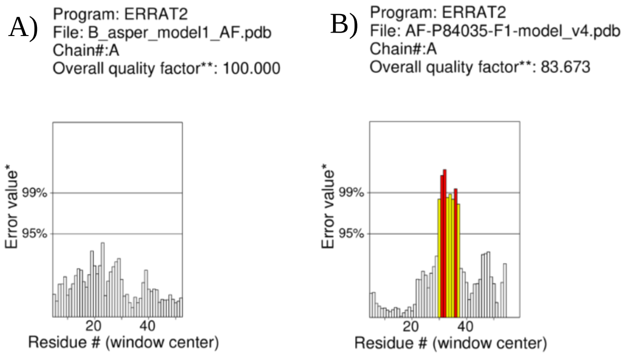

FS6.2. ERRAT. A) Structural validation of the predicted model for Basaprin from *Bothrops asper*. B) Structural validation of Basaprin from UniProt database [P84035].

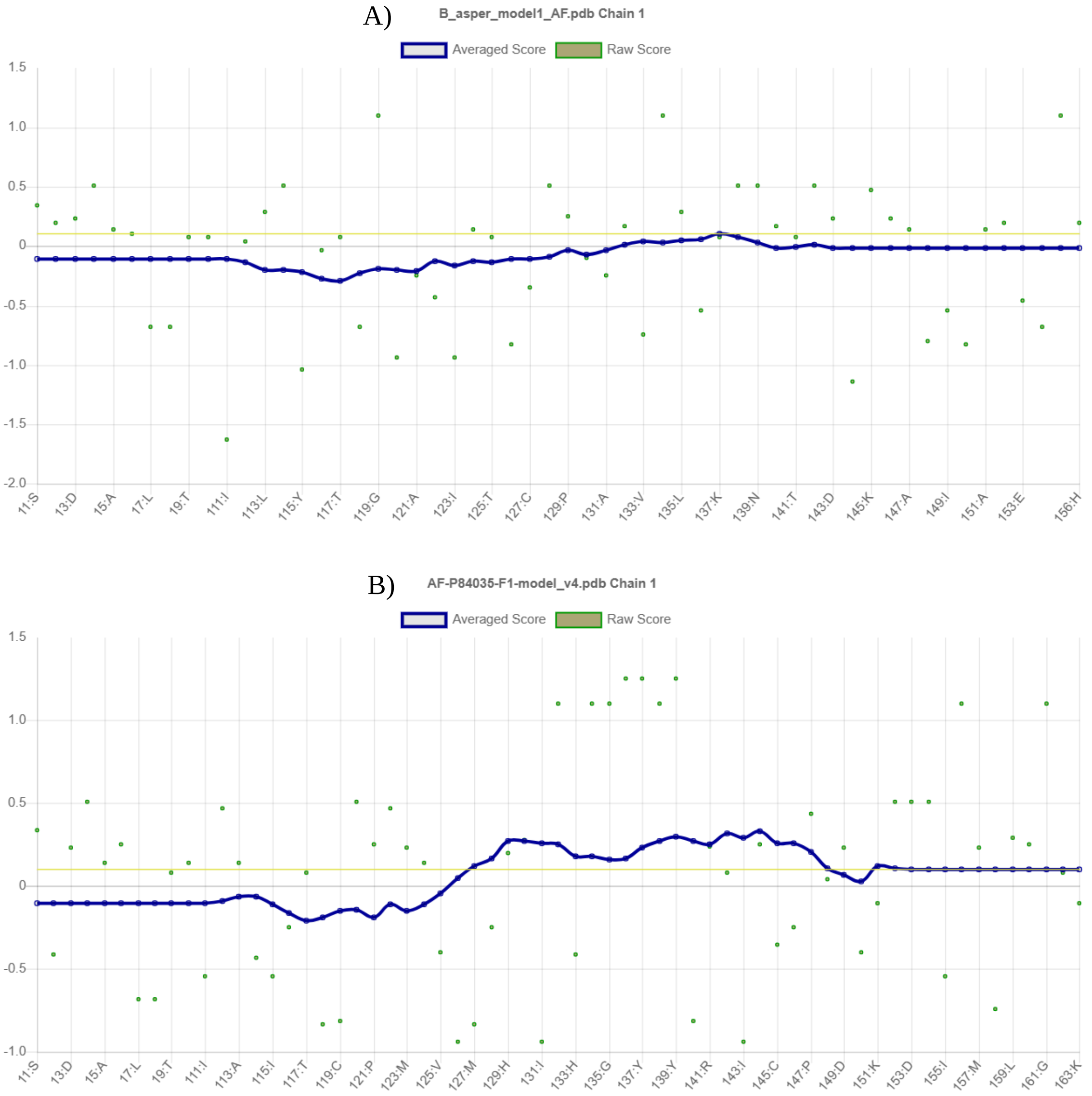

FS6.3. Verify3D. A) Structural validation of the predicted model for Basaprin from *Bothrops asper*. B) Structural validation of Basaprin from UniProt database [P84035].

8. Bothropasin

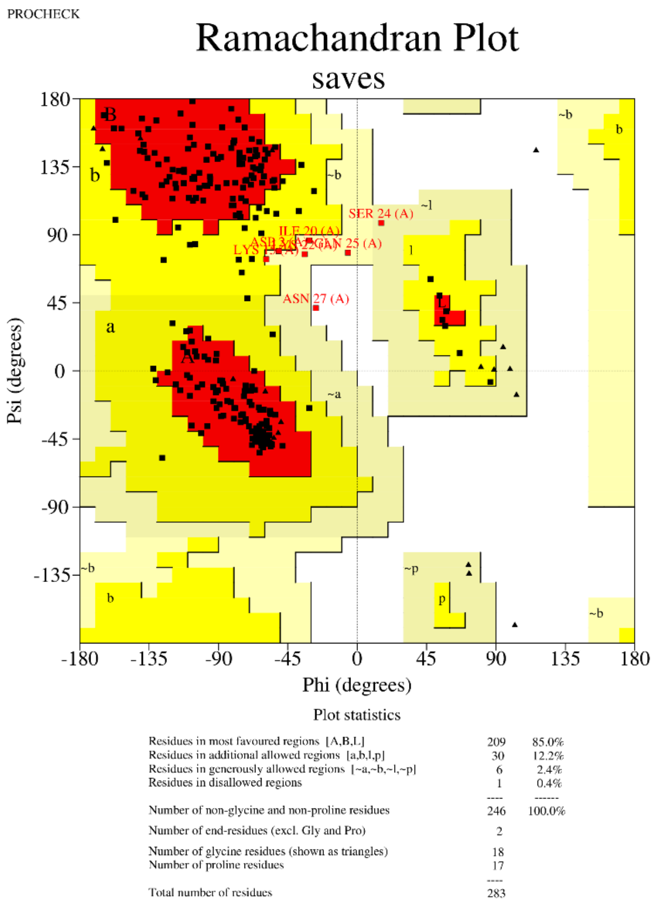

FS7.1 Ramachandran plots. Structural validation of bothropasin models of *Bothrops asper*.

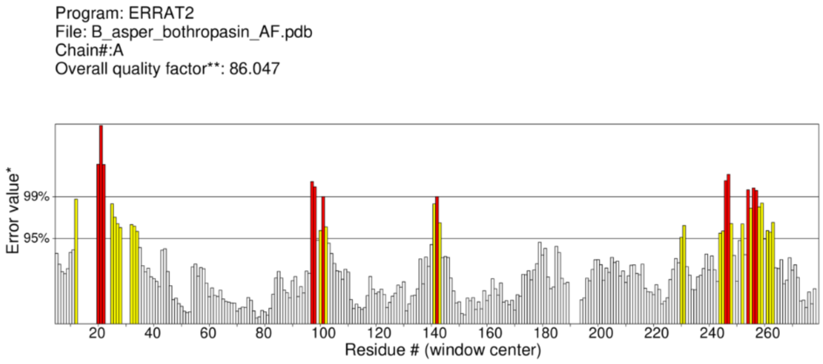

FS7.2. ERRAT. Structural validation of bothropasin models of *Bothrops asper*.

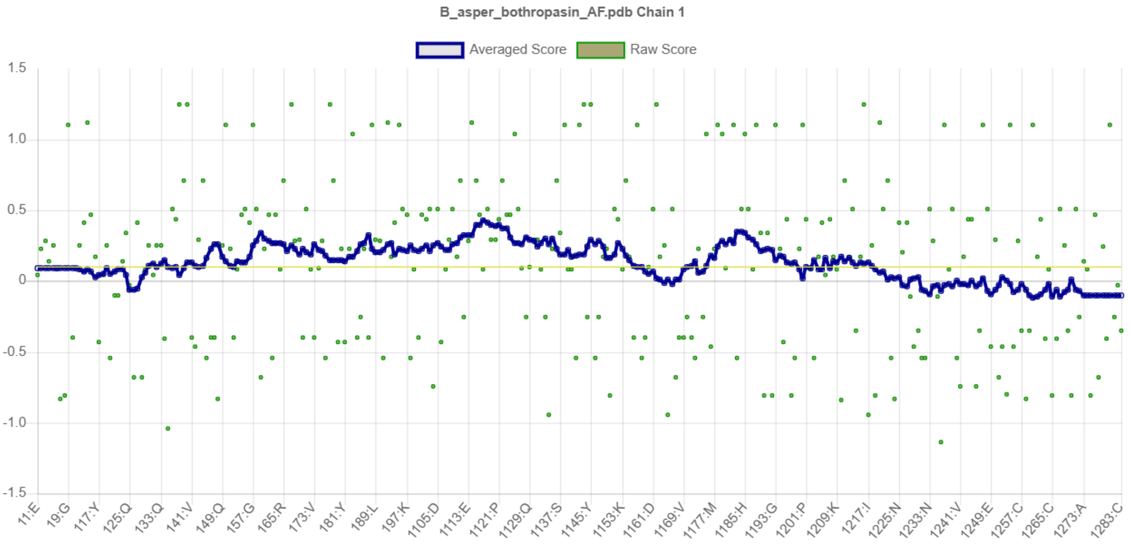

FS7.3. Verify3D. Structural validation of bothropasin models of *Bothrops asper*.
