# Supplementary material for: Computational Modeling of Low-Abundance Proteins in Venom Gland Transcriptomes: Bothrops asper and Bothrops jararaca
Source: Toxins (Basel). 2025 May 22;17(6):262. doi: 10.3390/toxins17060262 (PMC12197698; doi:10.3390/toxins17060262)
Supplement: Supplementary file 1 [file toxins-17-00262-s001.zip › Supplementary Material 3/Supplementary Material 3.pdf]

Computational Modeling of Low-Abundance Proteins in Venom Gland Transcriptomes: *Bothrops asper* and *Bothrops jararaca*

Joseph Espín-Angulo and Doris Vela*

Supplementary Material 3

Table S1. *Bothrops asper* TPM abundance analysis.

Protein	Total TPM	Total reads	# Contigs	% TPM
Botrocetin	6332.77071	26335.302	17	2.16
C-type Lectin	9630.28842	10109.599	35	3.29
Cysteine-rich protein	35.06138	333.593	5	0.01
Dihydroorotate dehydrogenase	45.41007	338.192	4	0.02
L-amino acid oxidase	2897.44097	48551.505	10	0.99
Metalloproteinase	170147.9805	423170.994	319	58.08
Serine protease	26875.22575	50473.221	128	9.17
Von Willebrand Factor	179.59367	1087.996	30	0.06
Von Willebrand Factor Type D	13.17625	25.927	3	0.01
Arylsulfatase	47.19779	829.578	8	0.02
Bradykinin potentiating	21279.60052	129848.198	10	7.26
Lysophospholipase D	108.86689	728.597	9	0.04
Phospholipase A2	54674.01259	75274.971	96	18.66
Phospholipase A2 inhibitor	421.35083	2538.889	13	0.14
Snake venom vascular endothelial growth factor	250.07877	1489.99	2	0.09

Table S2. *Bothrops jararaca* TPM abundance analysis.

Protein	Total TPM	Total reads	# Contigs	% TPM
Botrocetin	1.10E+03	1116.392	7	0.65
C-type Lectin	2.45E+04	32565.466	96	14.57
Cysteine-rich protein	8.22E+01	579.846	10	0.05
Dihydroorotate dehydrogenase	5.45E+01	584.475	5	0.03
L-amino acid oxidase	1.10E+03	21430.553	13	0.66
Metalloproteinase	1.00E+05	158583.907	436	59.73
Serine protease	3.32E+04	50351.152	183	19.75
Von Willebrand Factor	4.13E+02	6528.209	34	0.24
Von Willebrand Factor Type D	1.83E+00	0.973	1	0.01
Arylsulfatase	1.24E+02	1159.862	19	0.07
Bradykinin potentiating	3.53E+03	28780.307	9	2.1
Lysophospholipase D	2.13E+01	22.972	4	0.01
Phospholipase A2	2.39E+03	24854.252	61	1.43
Phospholipase A2 inhibitor	5.25E+02	2501.685	20	0.31
Snake venom vascular endothelial growth factor	6.50E+02	4644.82	8	0.39

These tables summarizes the total expression of each protein identified in the venom gland transcriptome of *Bothrops asper* and *Bothrops jararaca*, expressed in transcripts per million (TPM), total number of mapped reads, number of associated contigs, and the relative abundance percentage relative to the total transcriptome. While some proteins show low relative abundance (<0.10%), this does not imply that they lack biological relevance. In fact, when analyzed individually, they present multiple contigs with consistent TPM values, supporting their genuine presence. Detailed data by contig can be found in Supplementary Material 3 files (TPM_BA.zip, TPM_BJ.zip).
