# Supplementary figures and images for: Computational Modeling of Low-Abundance Proteins in Venom Gland Transcriptomes: Bothrops asper and Bothrops jararaca
Source: Toxins (Basel). 2025 May 22;17(6):262. doi: 10.3390/toxins17060262 (PMC12197698; doi:10.3390/toxins17060262)

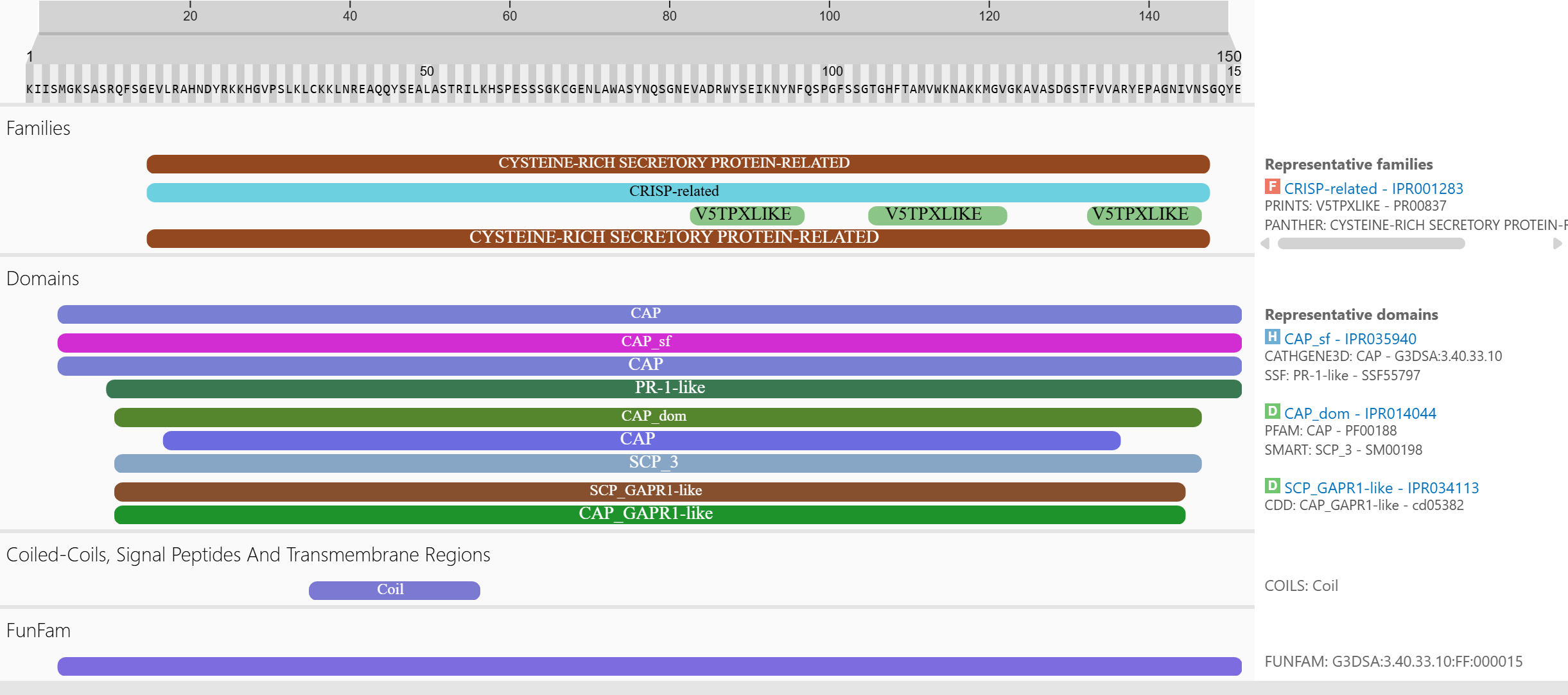

Supplement: Supplementary file 1 [file toxins-17-00262-s001.zip › Supplementary Material 1/File S1 CRISP/CRISP-B.asper_PFAM.png]

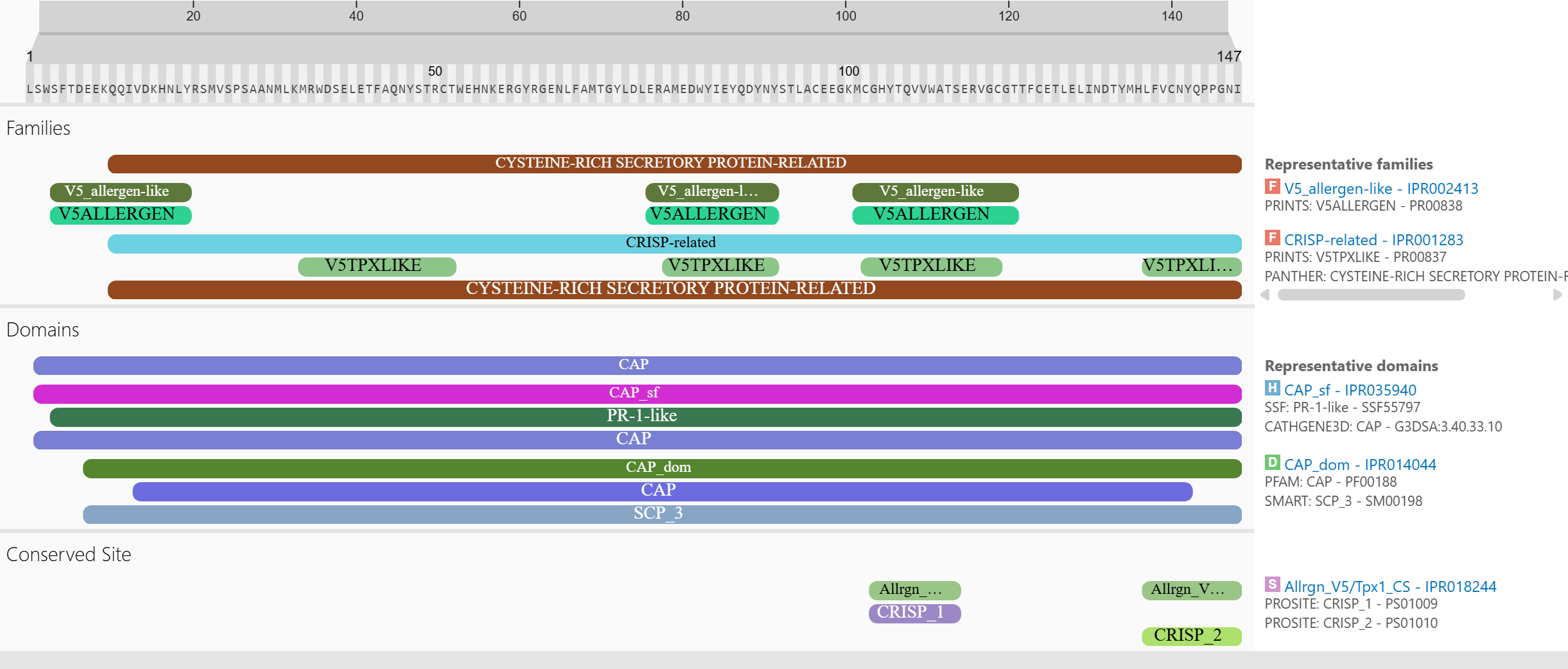

Supplement: Supplementary file 1 [file toxins-17-00262-s001.zip › Supplementary Material 1/File S1 CRISP/CRISP-B.jararaca_PFAM.png]

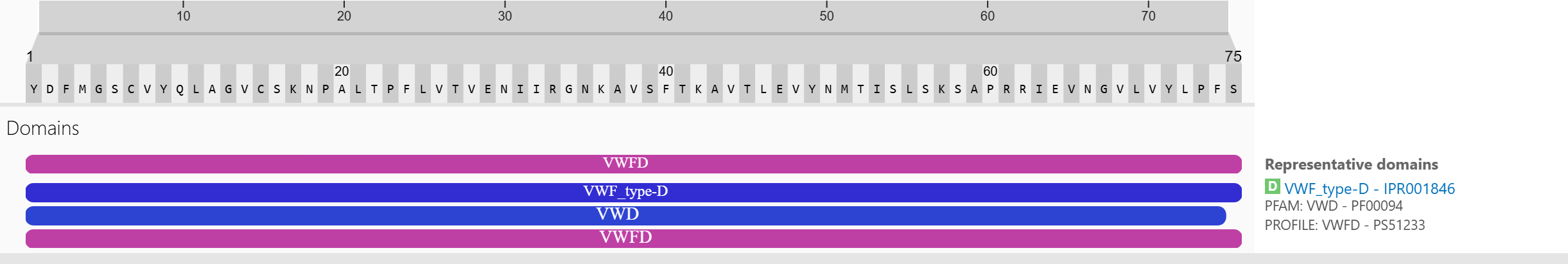

Supplement: Supplementary file 1 [file toxins-17-00262-s001.zip › Supplementary Material 1/File S2 Apolipophorin/Apolipophorin-B.asper_PFAM.png]

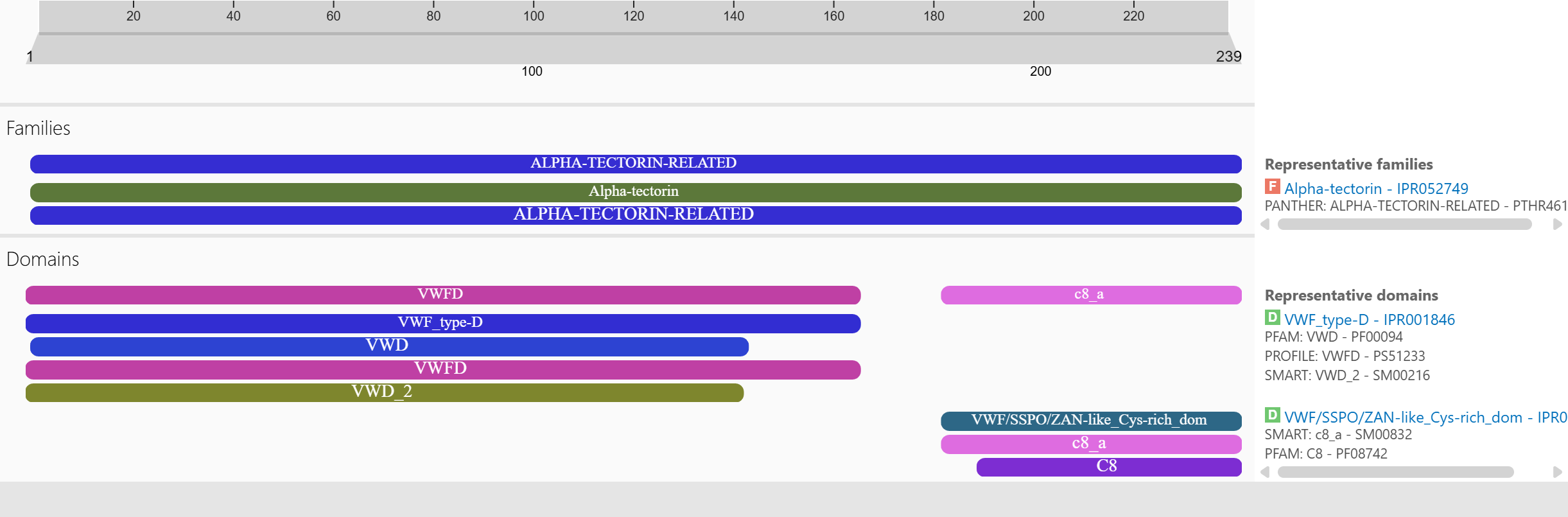

Supplement: Supplementary file 1 [file toxins-17-00262-s001.zip › Supplementary Material 1/File S2 Apolipophorin/Apolipophorin-B.jararaca_PFAM.png]

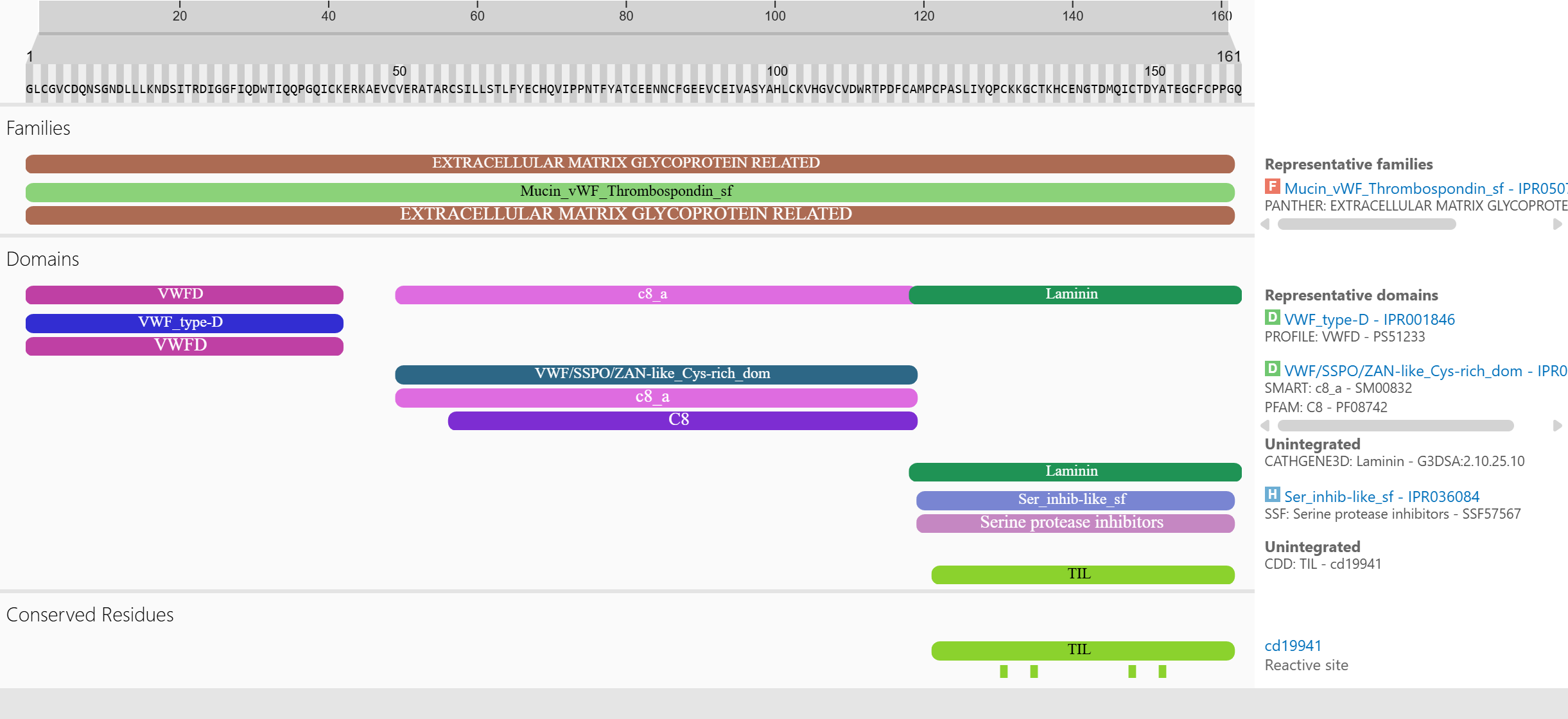

Supplement: Supplementary file 1 [file toxins-17-00262-s001.zip › Supplementary Material 1/File S2 Apolipophorin/vWFD/vWFD-B.asper_PFAM.png]

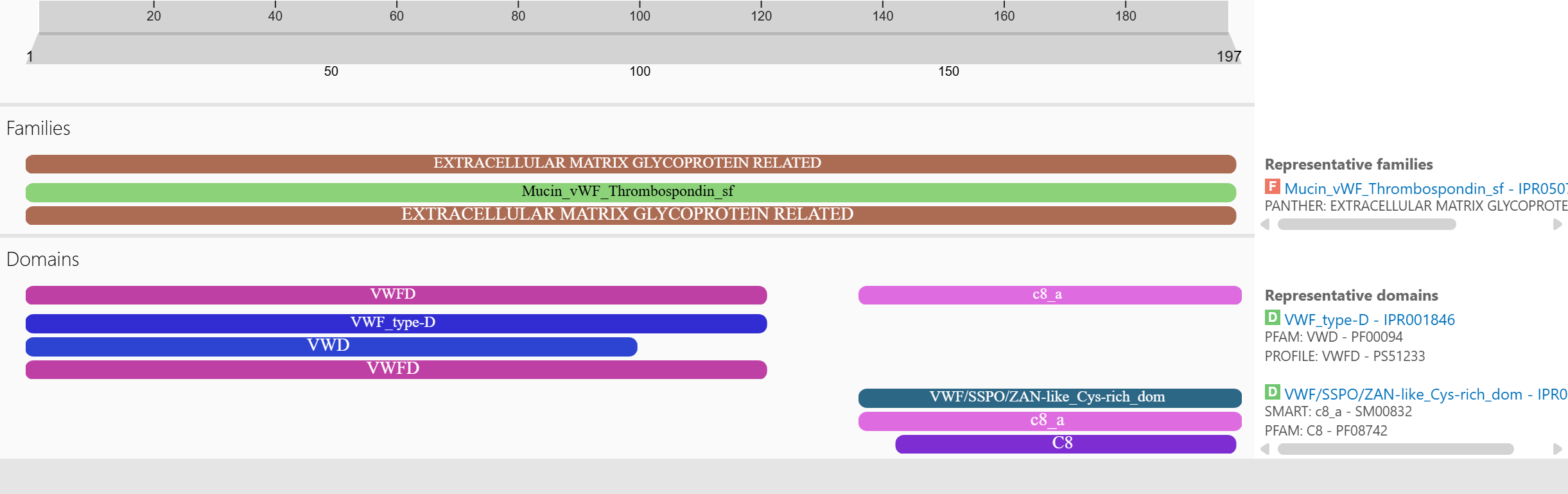

Supplement: Supplementary file 1 [file toxins-17-00262-s001.zip › Supplementary Material 1/File S2 Apolipophorin/vWFD/vWFD-B.jararaca_PFAM.png]

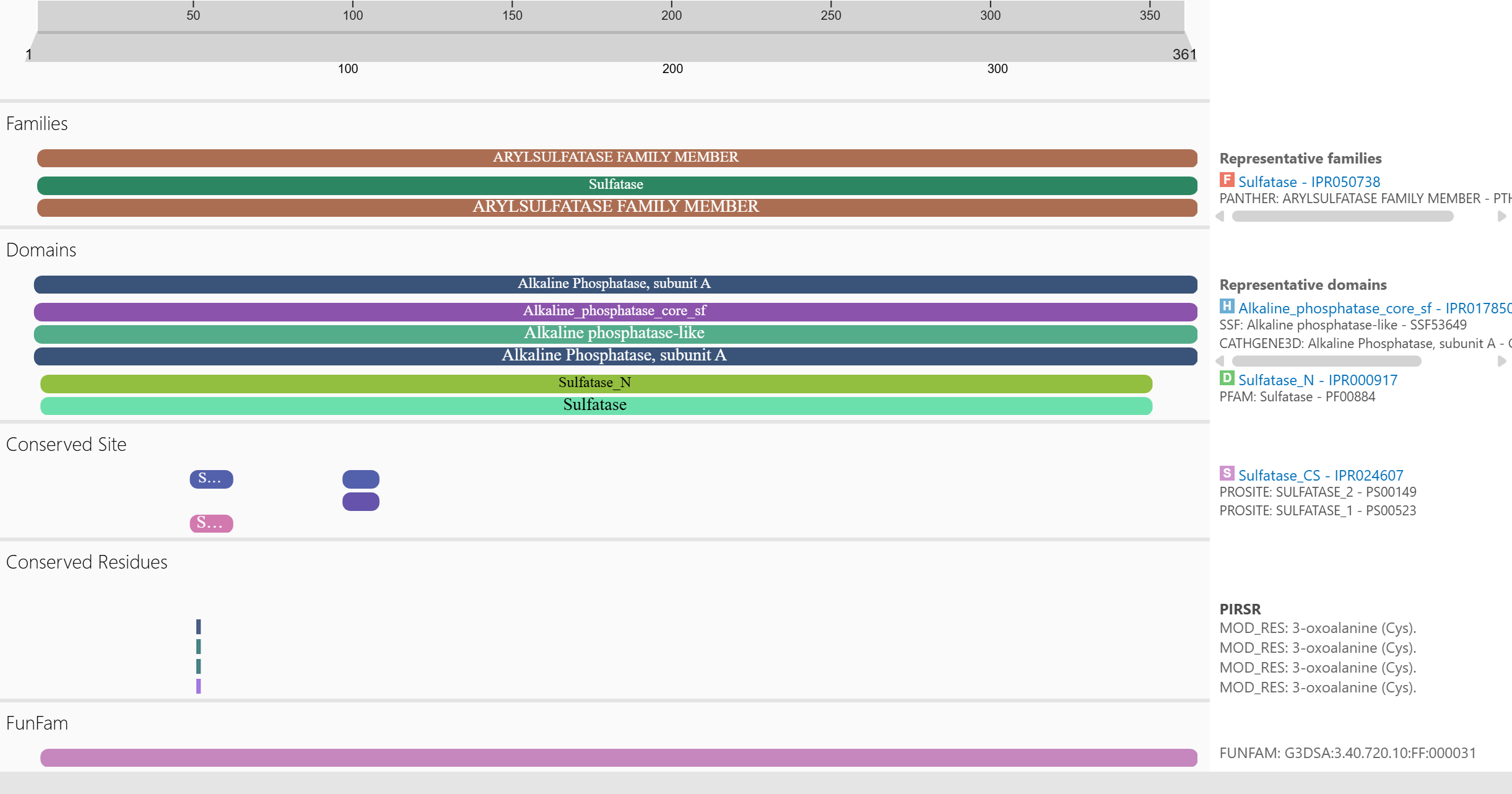

Supplement: Supplementary file 1 [file toxins-17-00262-s001.zip › Supplementary Material 1/File S3 ARSB/ARSB-B.asper_PFAM.png]

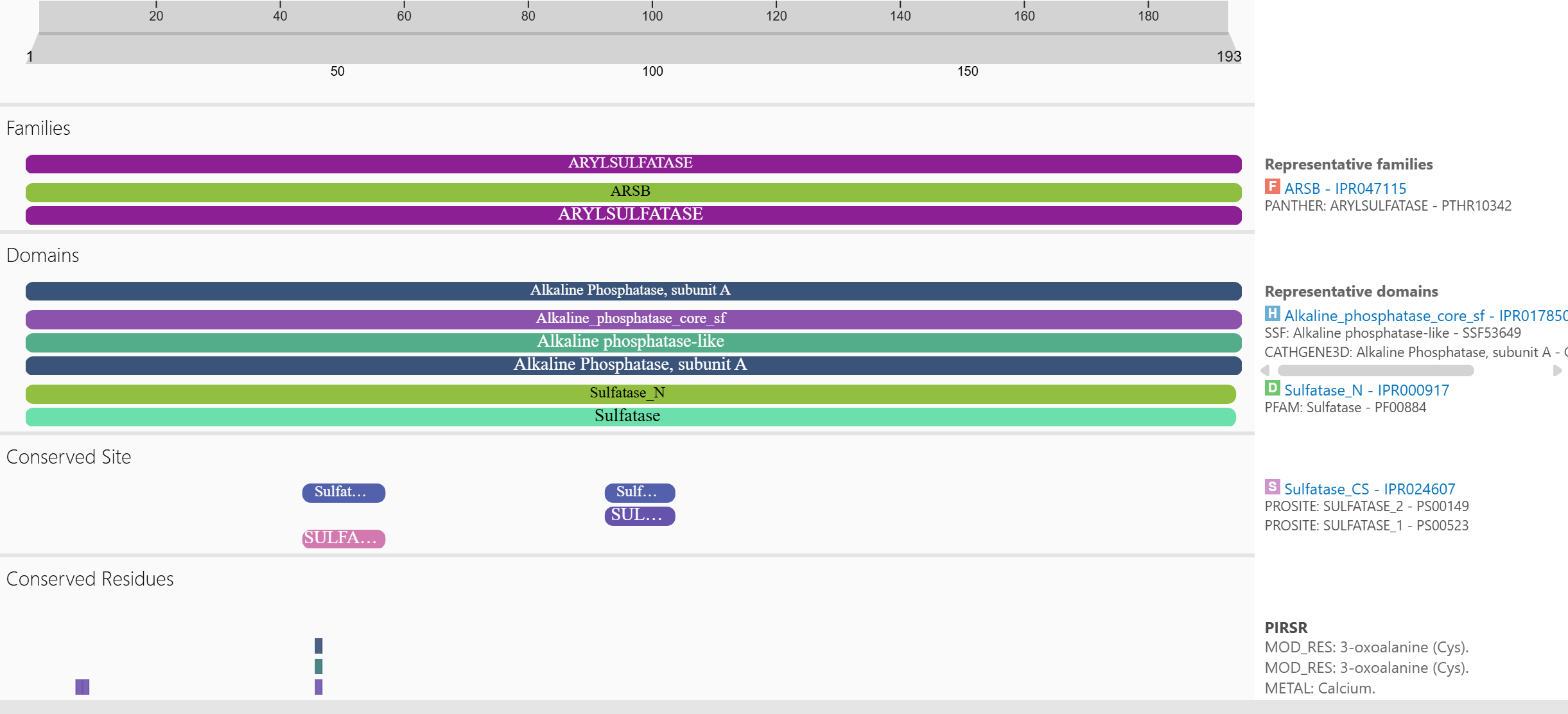

Supplement: Supplementary file 1 [file toxins-17-00262-s001.zip › Supplementary Material 1/File S3 ARSB/ARSB-B.jararaca_PFAM.png]

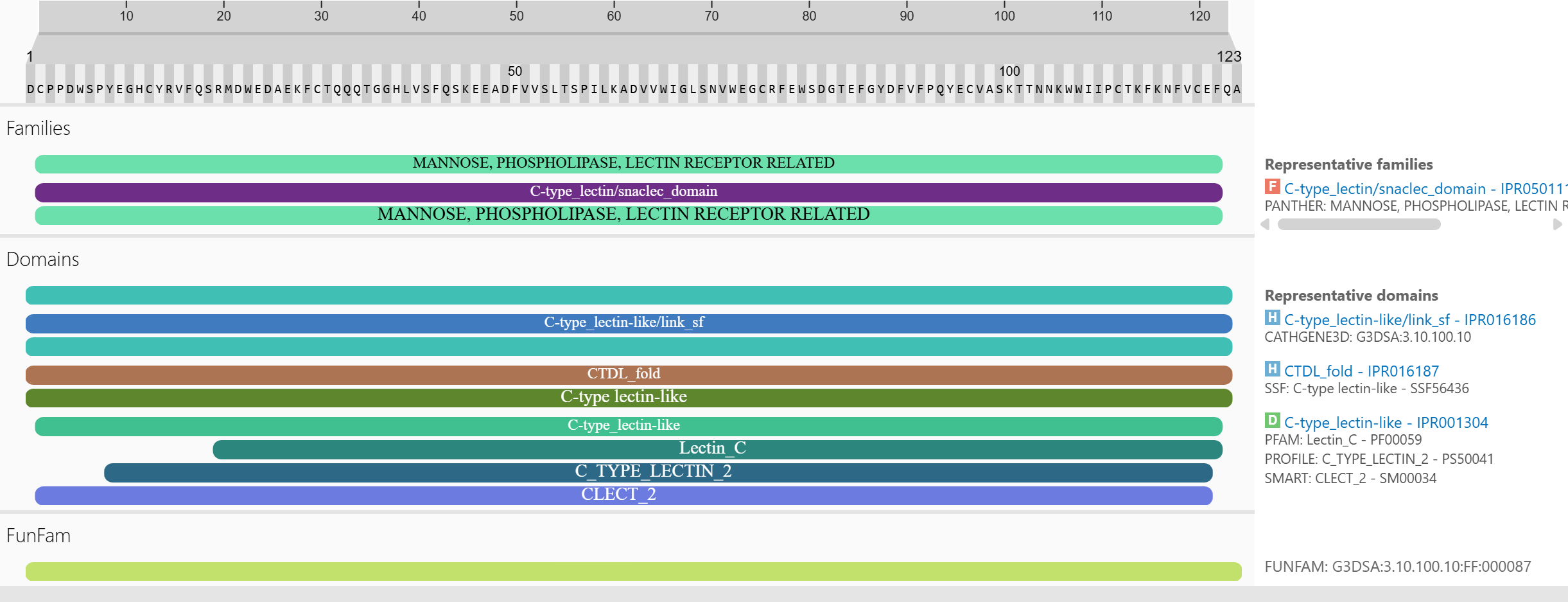

Supplement: Supplementary file 1 [file toxins-17-00262-s001.zip › Supplementary Material 1/File S4 botrocetin/Botrocetin-B.asper_PFAM.png]

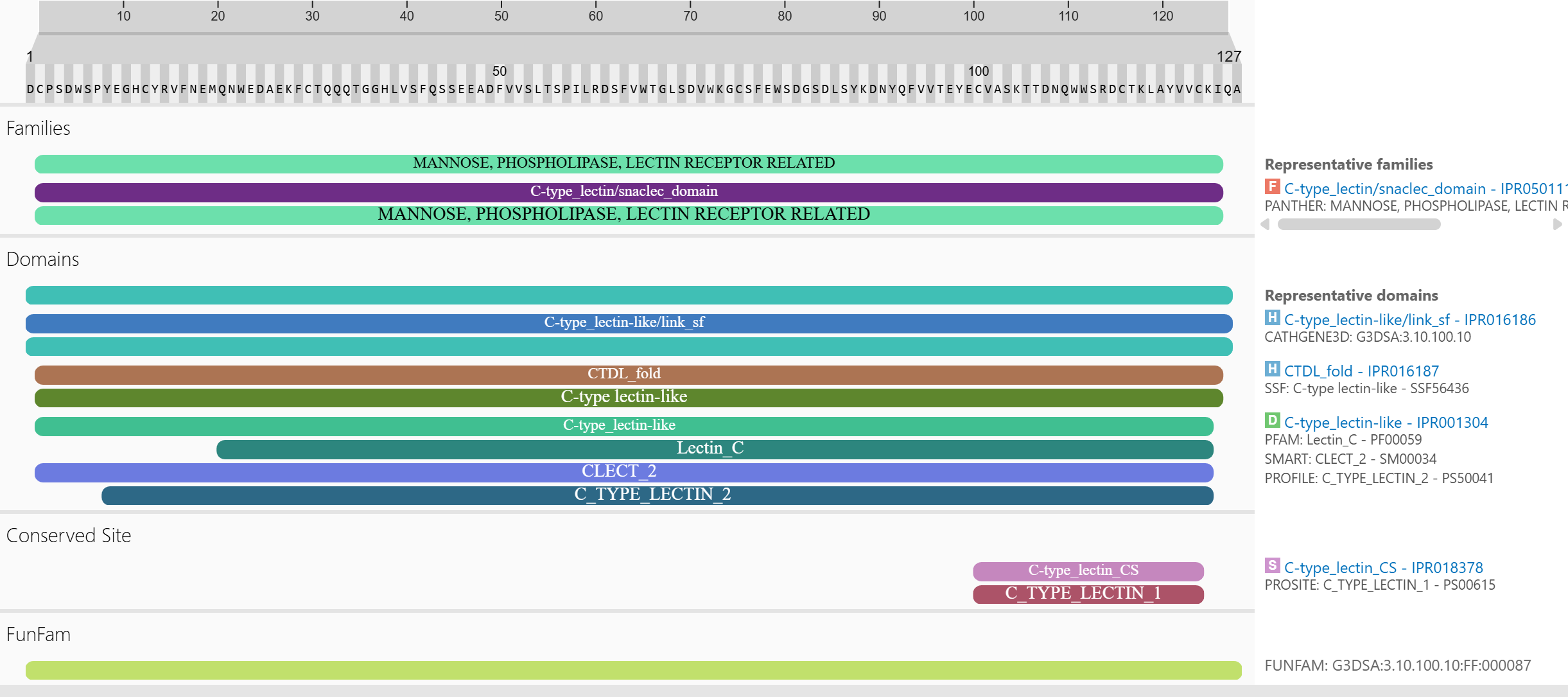

Supplement: Supplementary file 1 [file toxins-17-00262-s001.zip › Supplementary Material 1/File S4 botrocetin/Botrocetin-B.jararaca_PFAM.png]

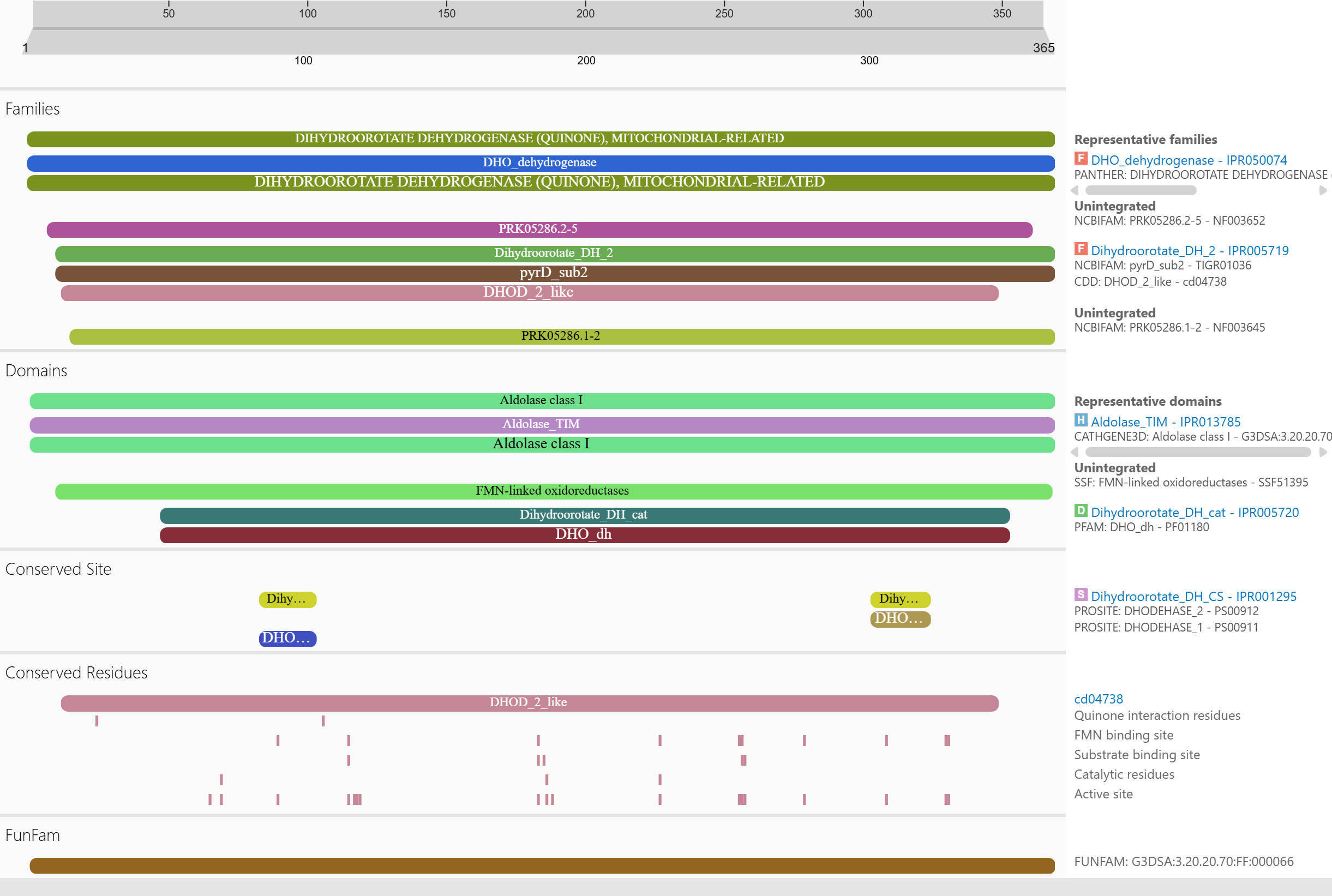

Supplement: Supplementary file 1 [file toxins-17-00262-s001.zip › Supplementary Material 1/File S5 DHODH/DHODH-B.asper_PFAM.png]

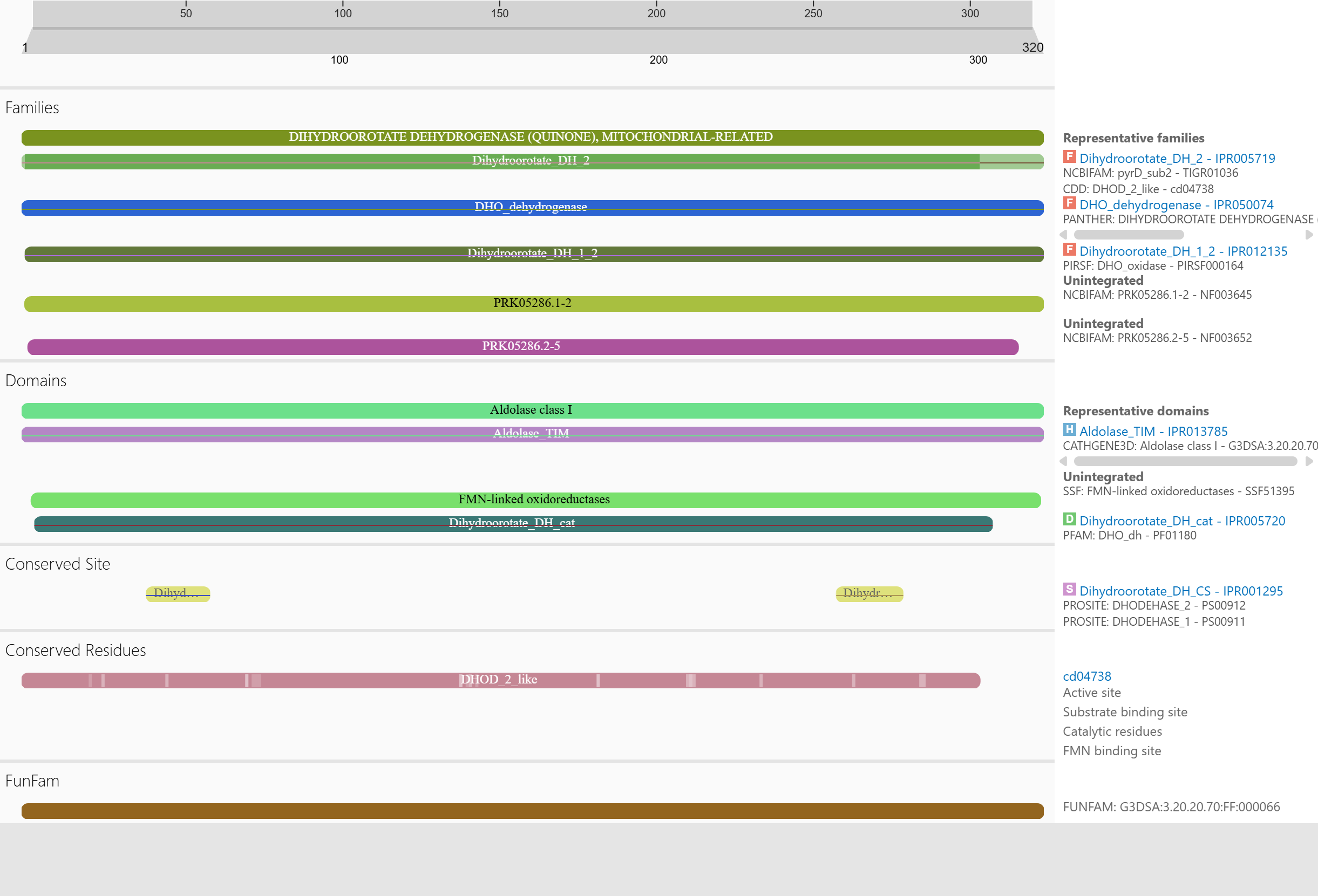

Supplement: Supplementary file 1 [file toxins-17-00262-s001.zip › Supplementary Material 1/File S5 DHODH/DHODH-B.jararaca_PFAM.png]

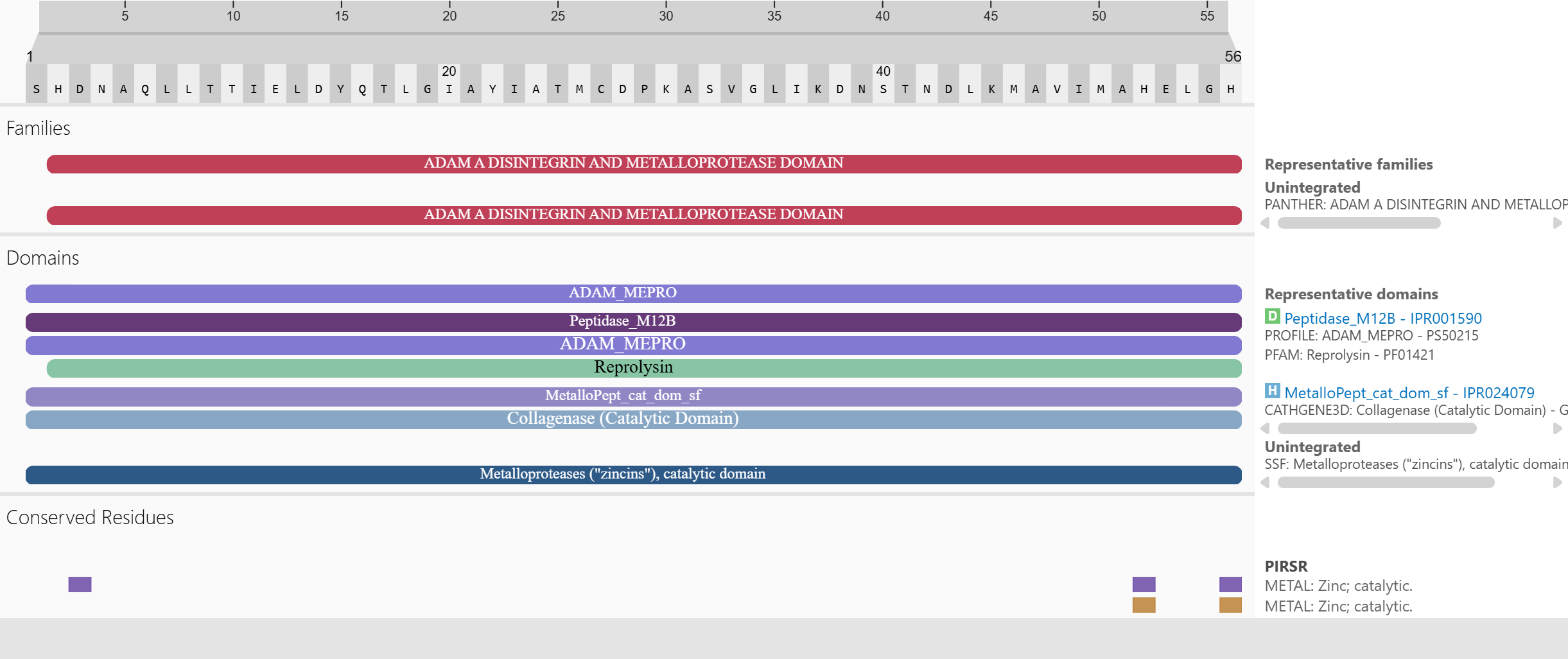

Supplement: Supplementary file 1 [file toxins-17-00262-s001.zip › Supplementary Material 1/File S6 basparin/Basparin-B.asper_PFAM.png]

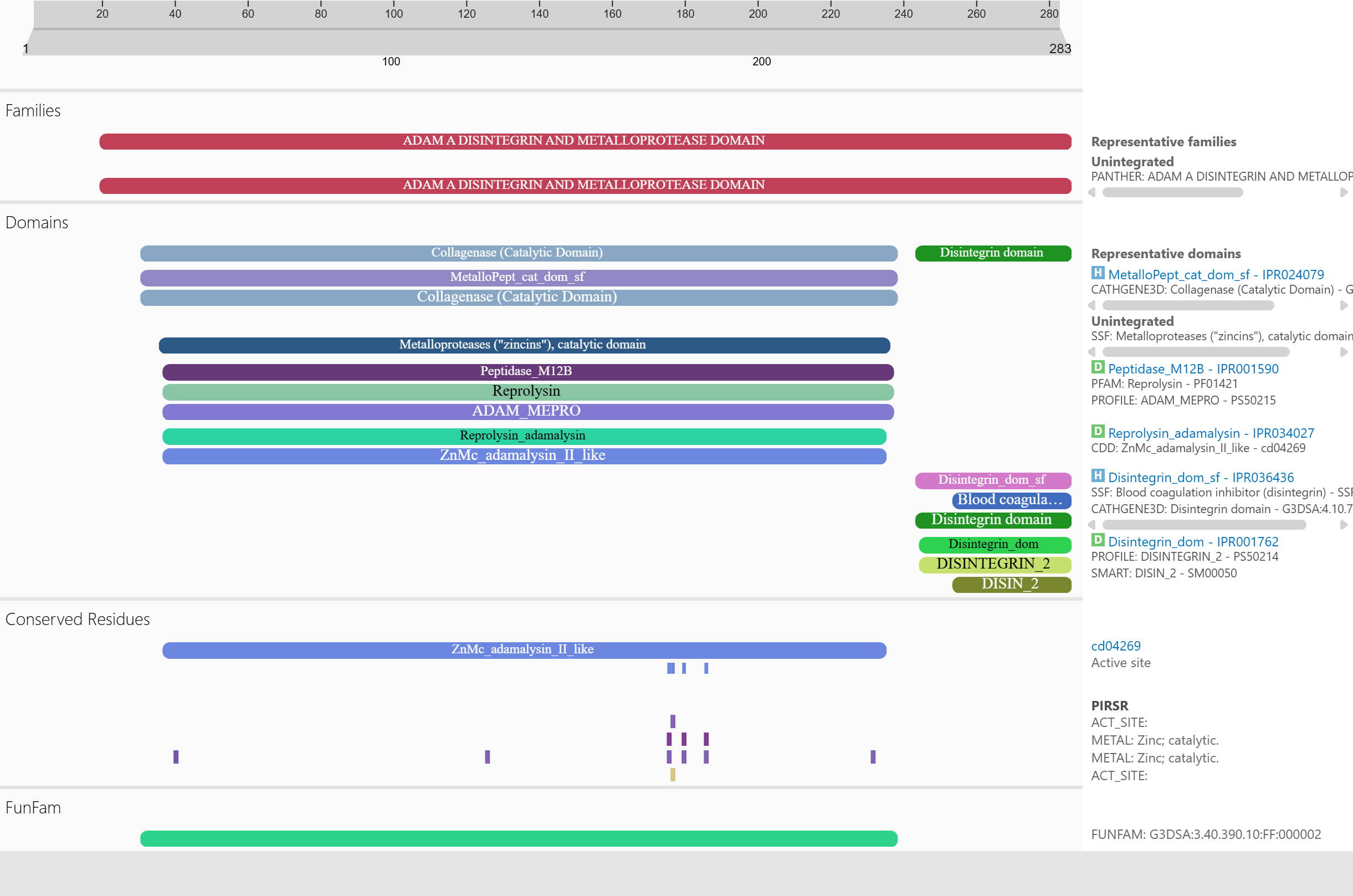

Supplement: Supplementary file 1 [file toxins-17-00262-s001.zip › Supplementary Material 1/File S6 basparin/Bothropasin/Bothropasin-B.asper_PFAM.png]

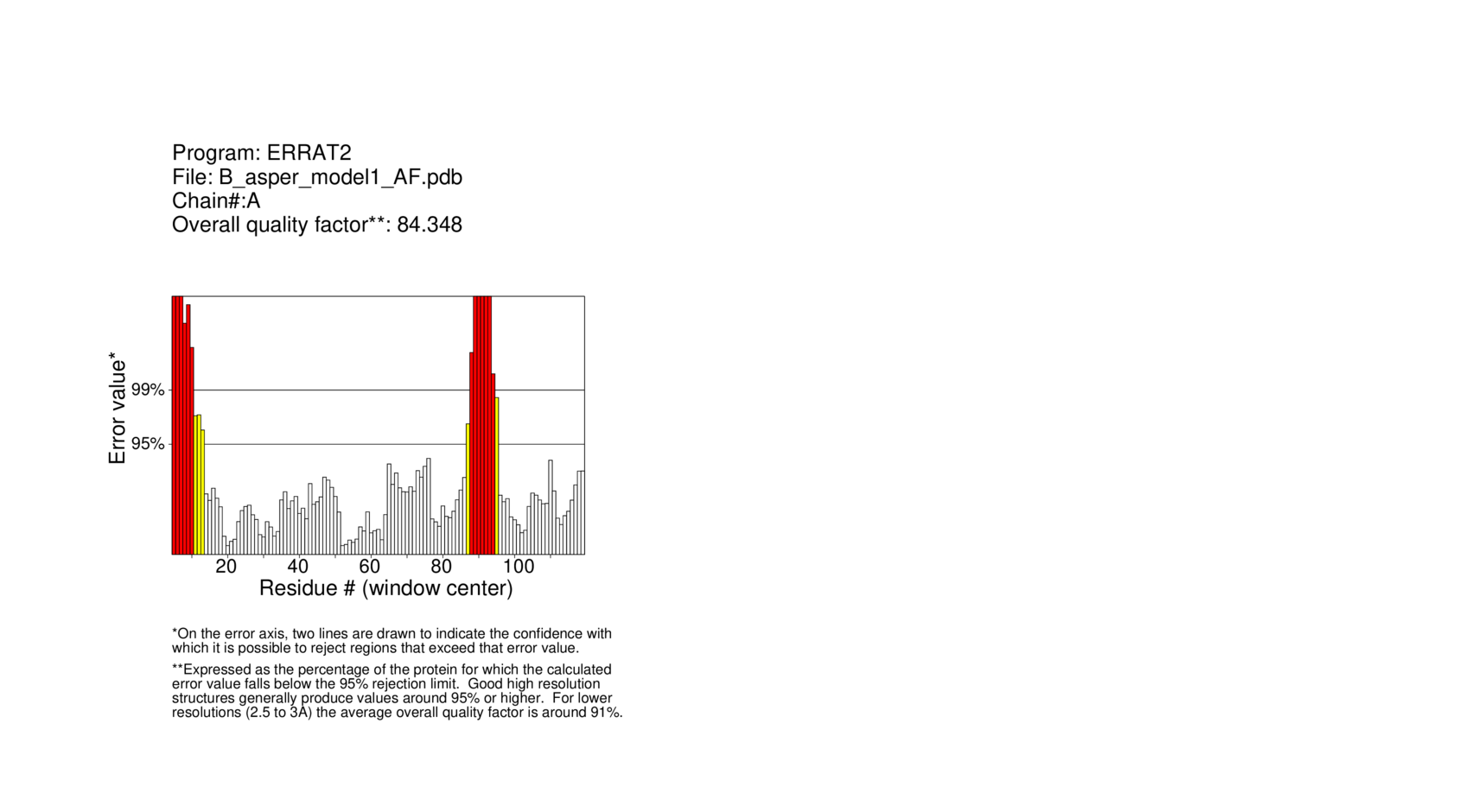

Supplement: Supplementary file 1 [file toxins-17-00262-s001.zip › Supplementary Material 2/File S10 Botrocetin/B.asper/BA-ERRAT.png]

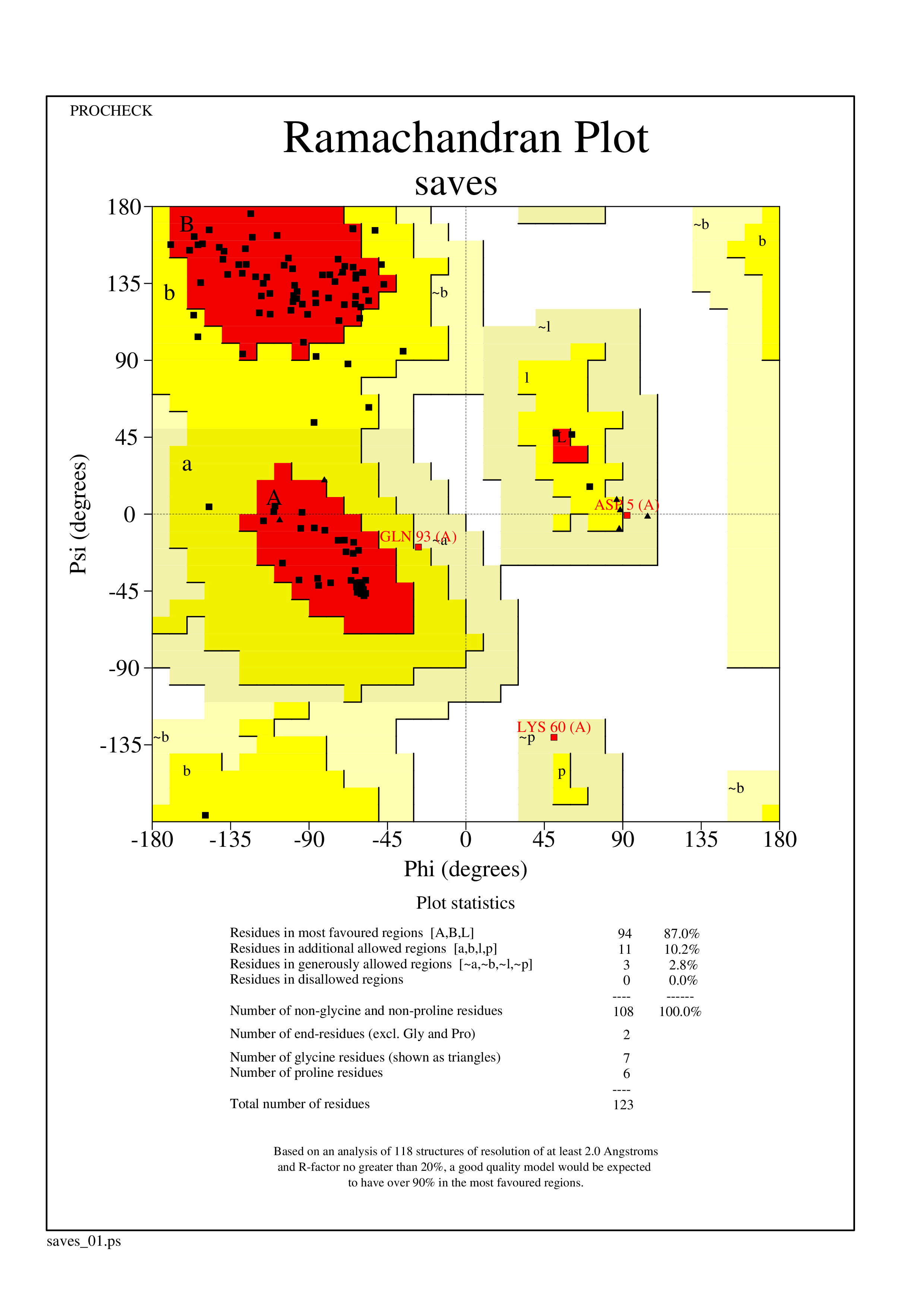

Supplement: Supplementary file 1 [file toxins-17-00262-s001.zip › Supplementary Material 2/File S10 Botrocetin/B.asper/BA-RAMACH.png]

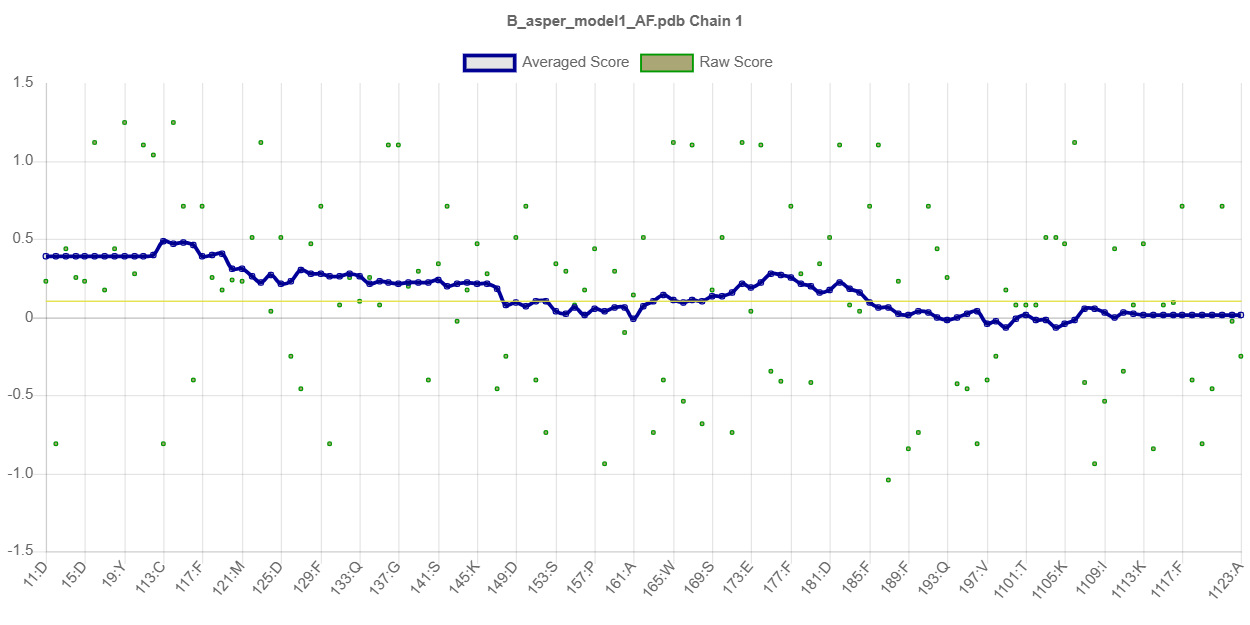

Supplement: Supplementary file 1 [file toxins-17-00262-s001.zip › Supplementary Material 2/File S10 Botrocetin/B.asper/BA-VF3D.png]

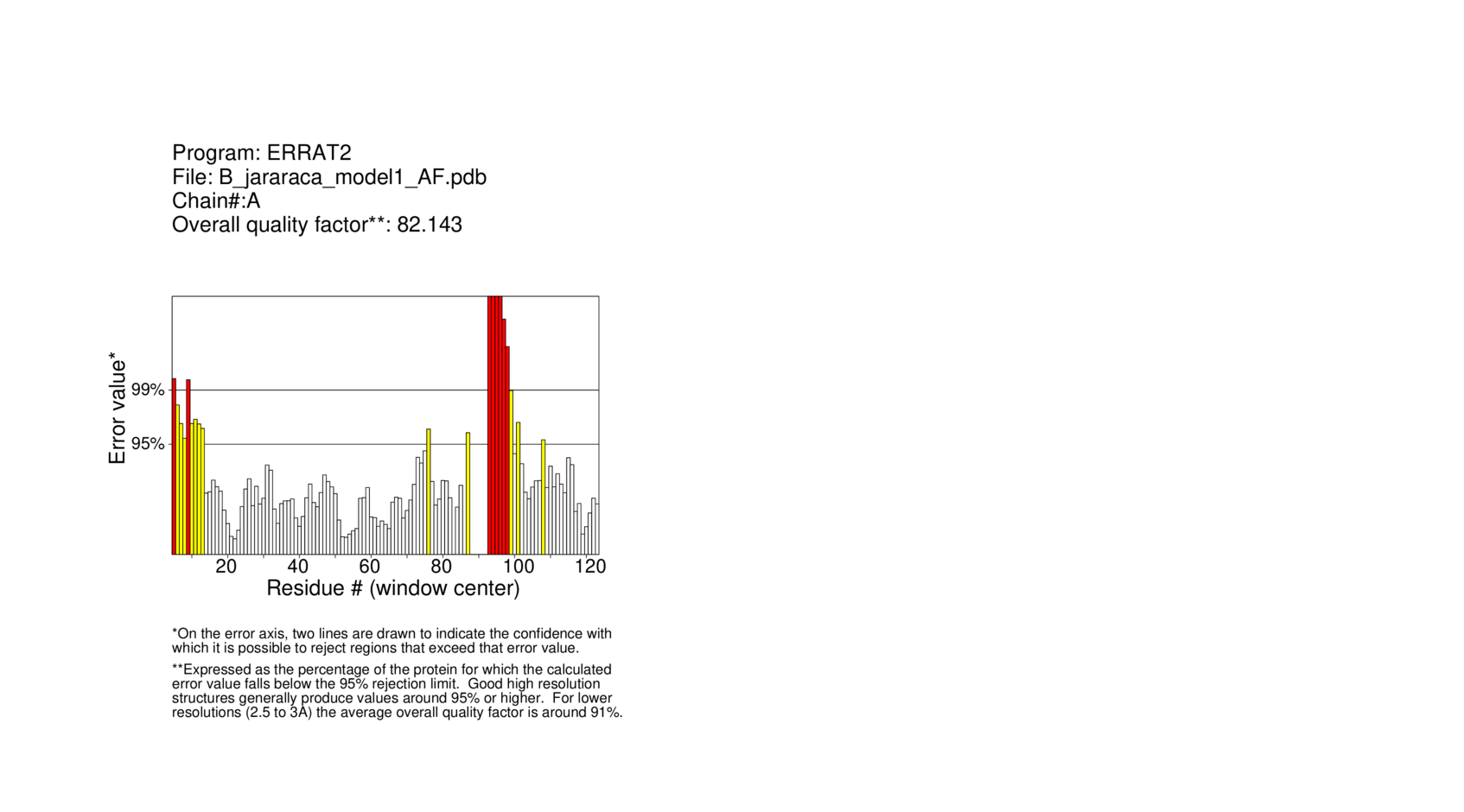

Supplement: Supplementary file 1 [file toxins-17-00262-s001.zip › Supplementary Material 2/File S10 Botrocetin/B.jararaca/BJ-ERRAT.png]

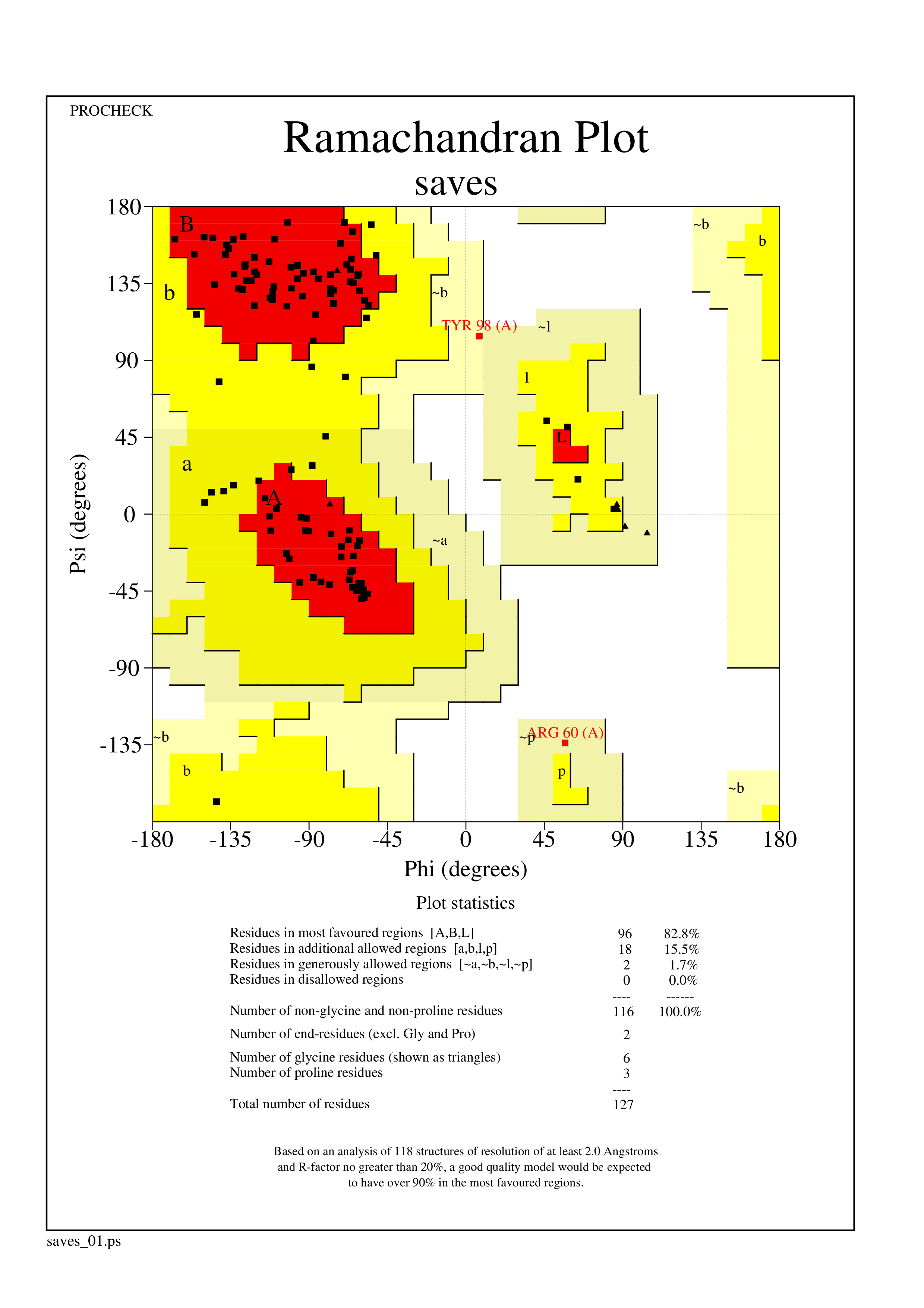

Supplement: Supplementary file 1 [file toxins-17-00262-s001.zip › Supplementary Material 2/File S10 Botrocetin/B.jararaca/BJ-PROCHE.png]

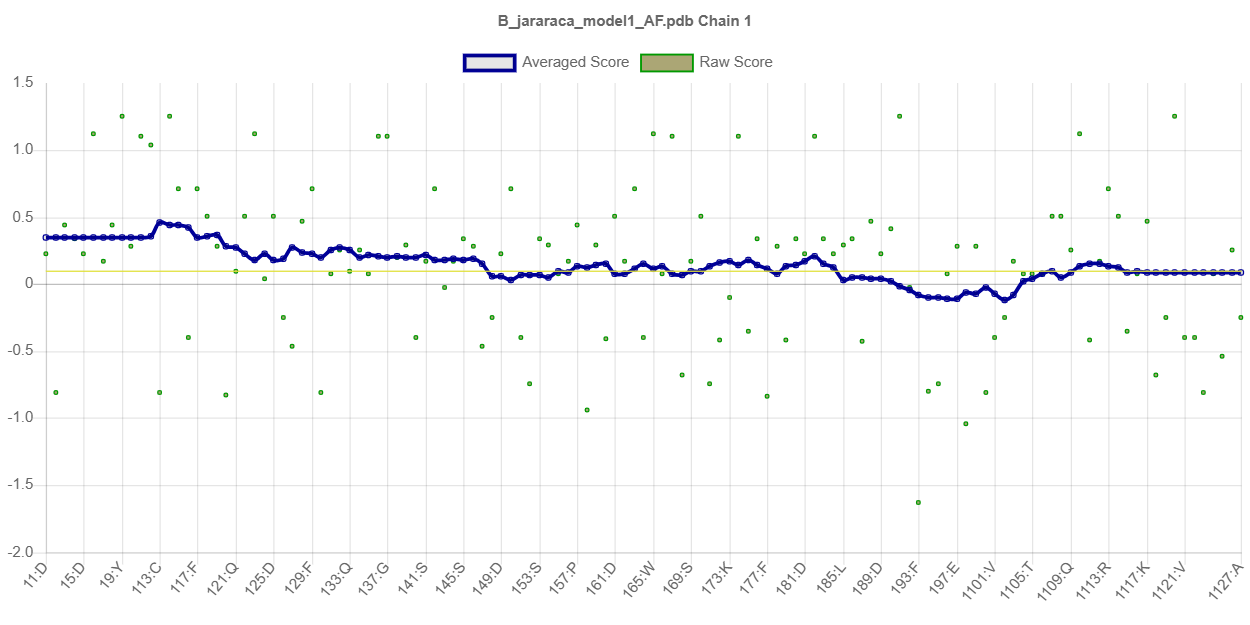

Supplement: Supplementary file 1 [file toxins-17-00262-s001.zip › Supplementary Material 2/File S10 Botrocetin/B.jararaca/BJ-VF3D.png]

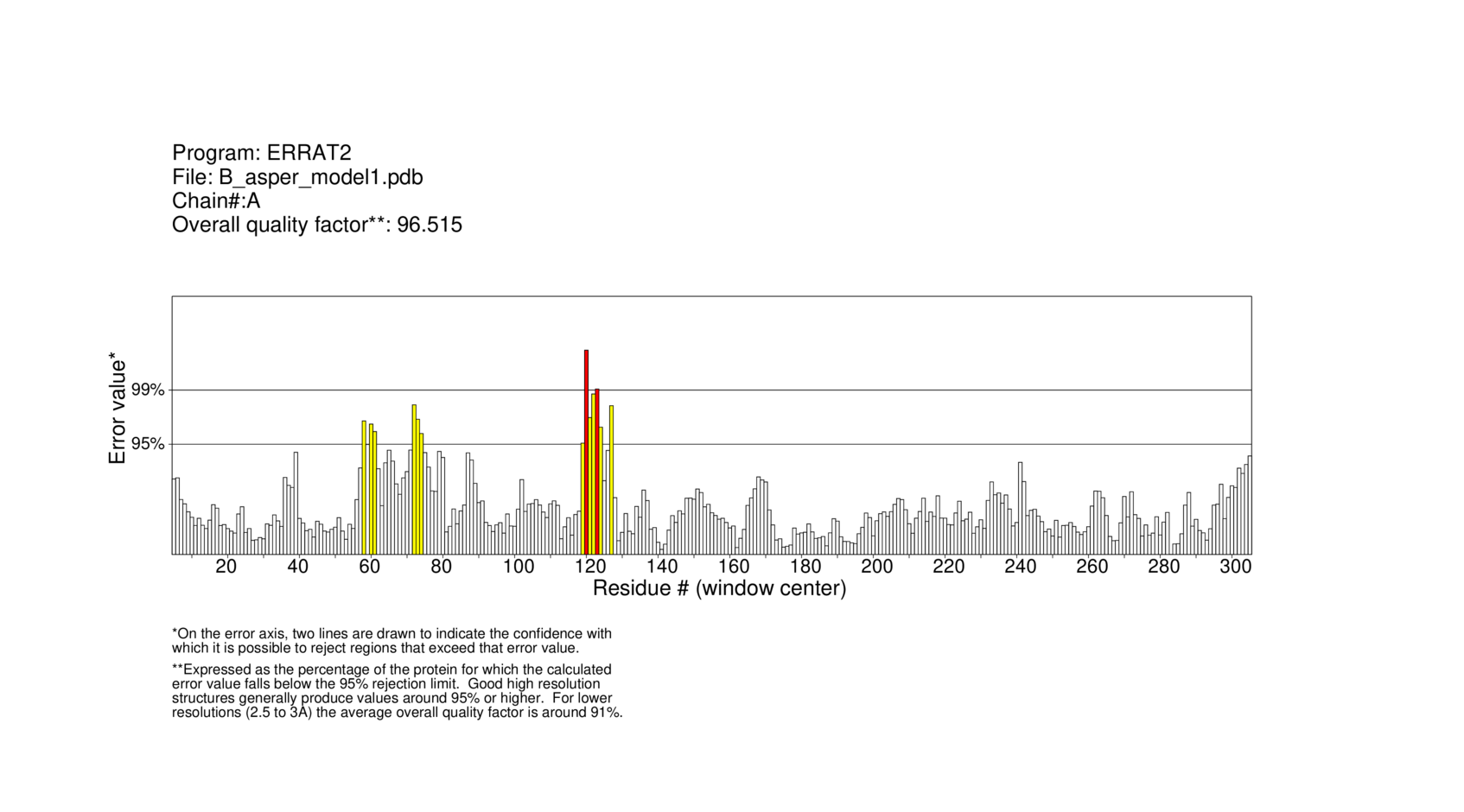

Supplement: Supplementary file 1 [file toxins-17-00262-s001.zip › Supplementary Material 2/File S11 DHODH/B.asper/BA-ERRAT.png]

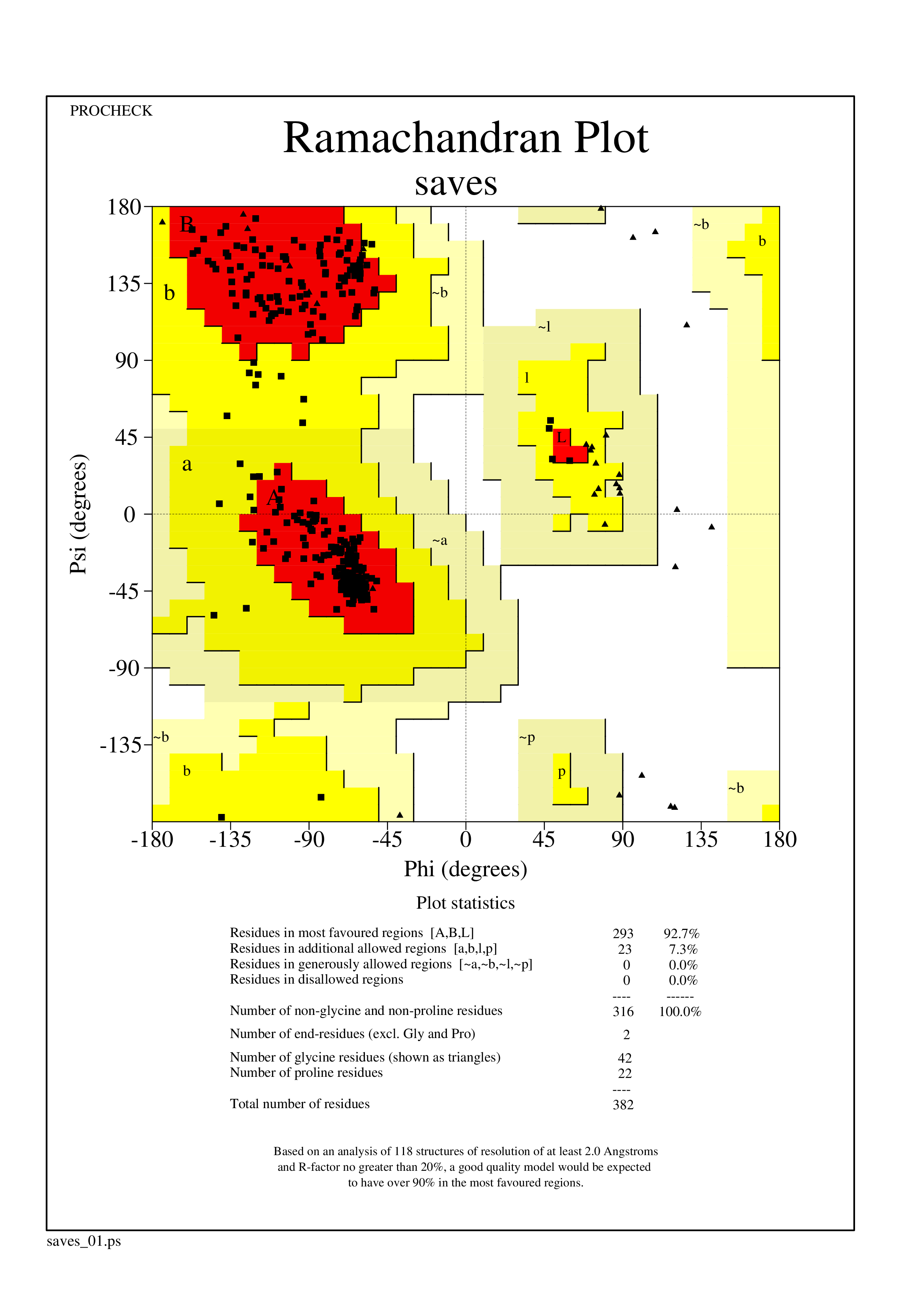

Supplement: Supplementary file 1 [file toxins-17-00262-s001.zip › Supplementary Material 2/File S11 DHODH/B.asper/BA-RAMACH.png]

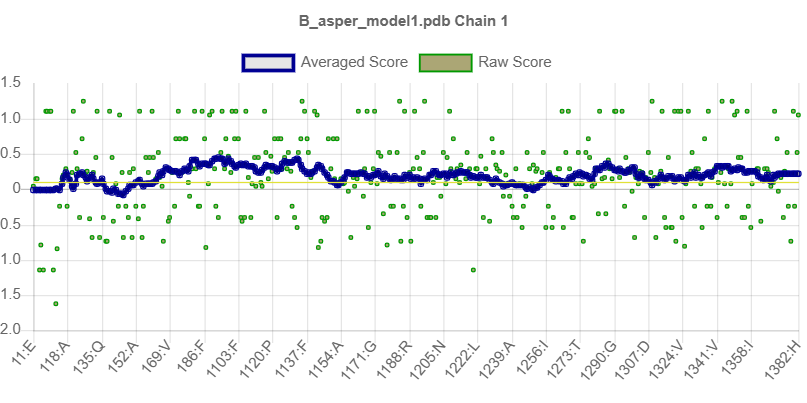

Supplement: Supplementary file 1 [file toxins-17-00262-s001.zip › Supplementary Material 2/File S11 DHODH/B.asper/BA-VERIFY3D.png]

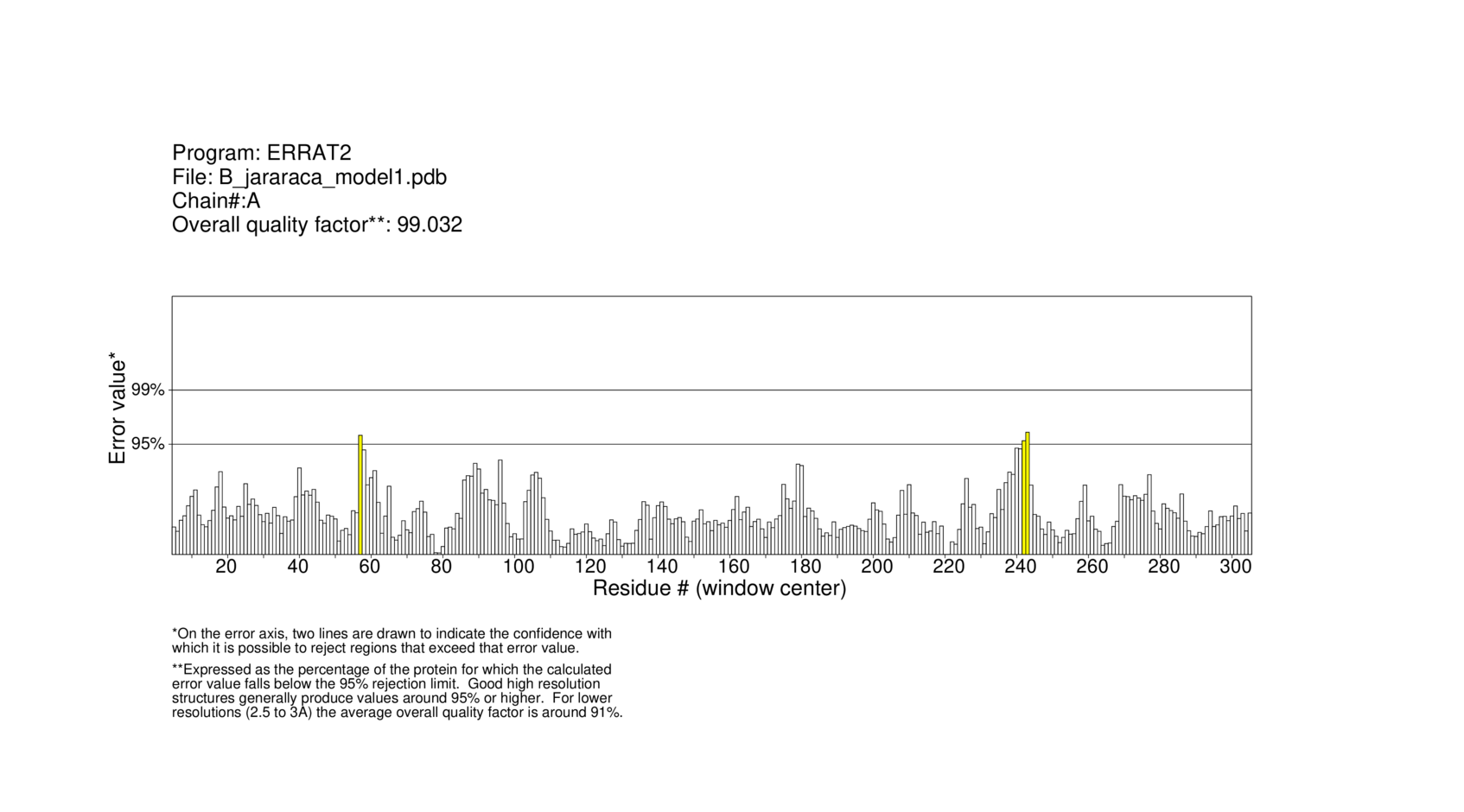

Supplement: Supplementary file 1 [file toxins-17-00262-s001.zip › Supplementary Material 2/File S11 DHODH/B.jararaca/BJ-ERRAT.png]

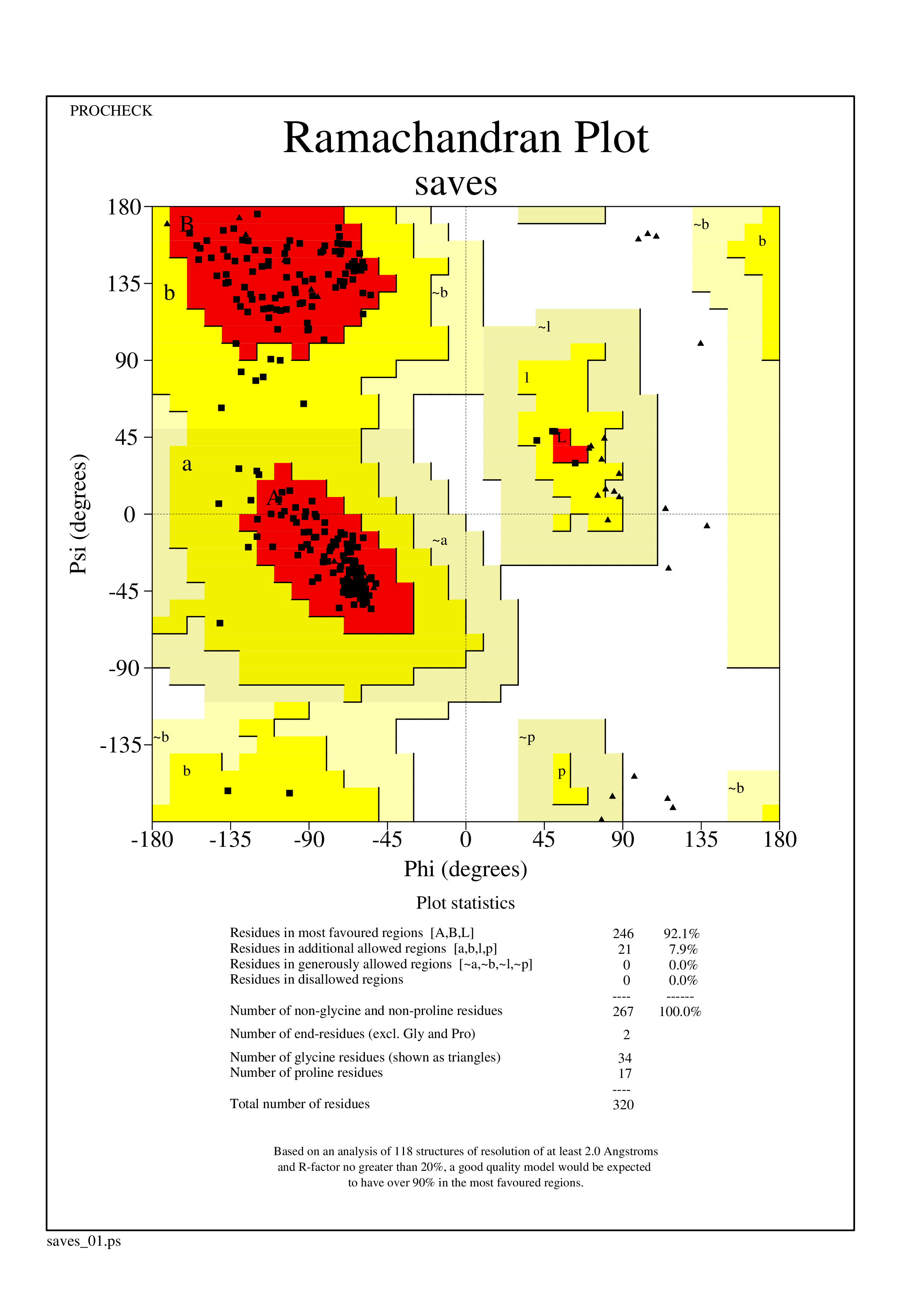

Supplement: Supplementary file 1 [file toxins-17-00262-s001.zip › Supplementary Material 2/File S11 DHODH/B.jararaca/BJ-RAMACH.png]

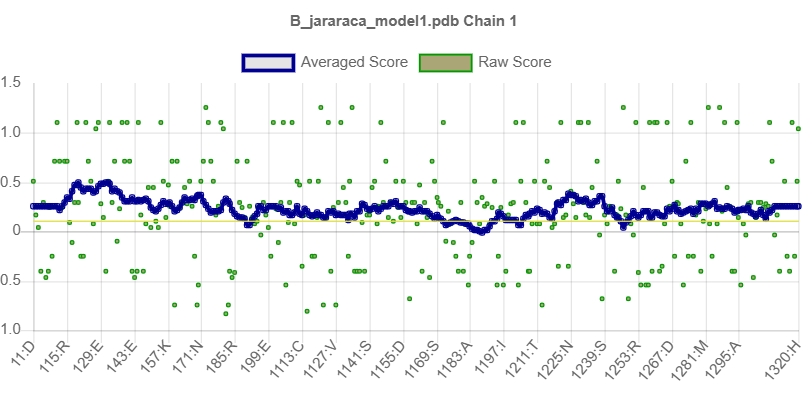

Supplement: Supplementary file 1 [file toxins-17-00262-s001.zip › Supplementary Material 2/File S11 DHODH/B.jararaca/BJ-VERIFY3D.png]

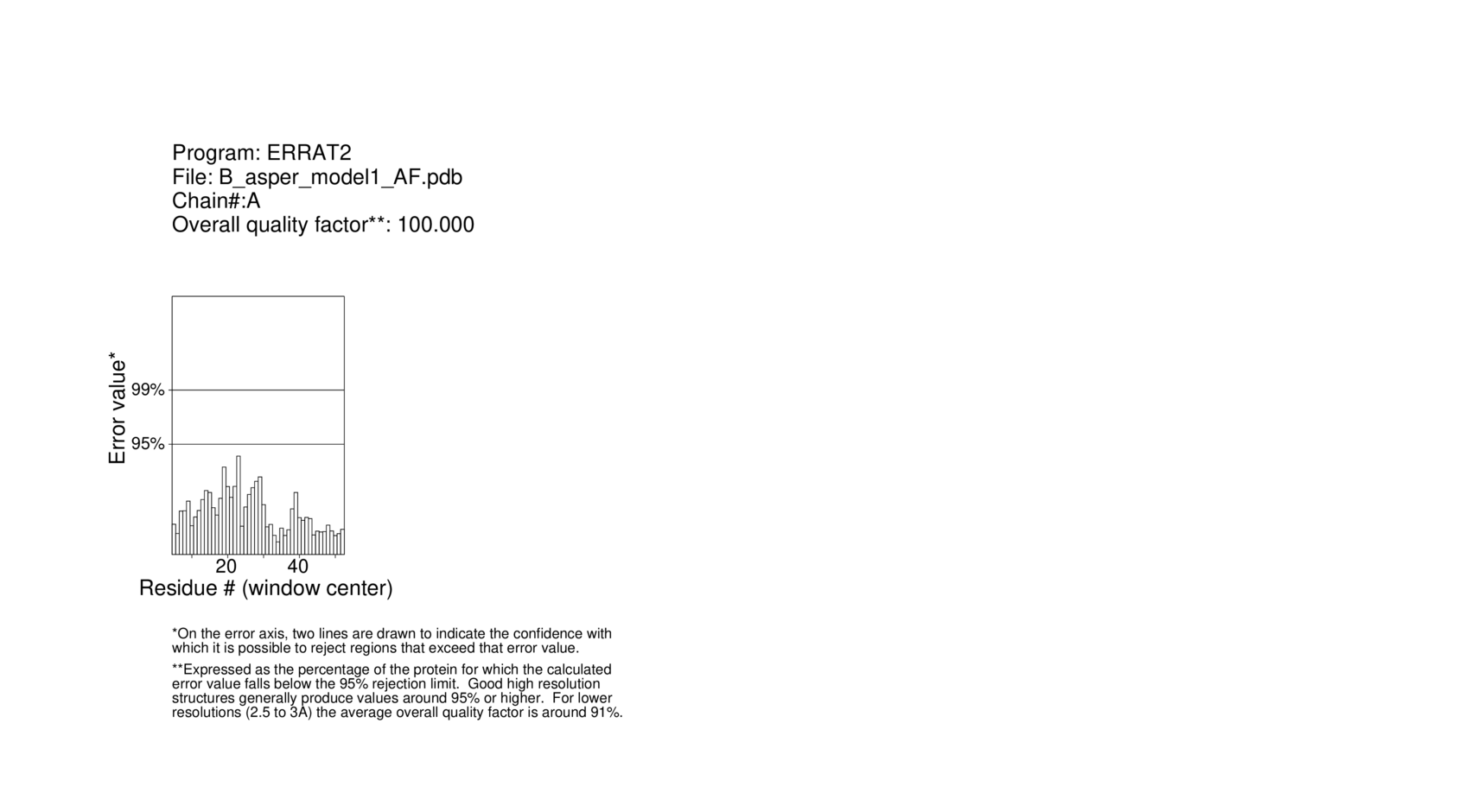

Supplement: Supplementary file 1 [file toxins-17-00262-s001.zip › Supplementary Material 2/File S12 Basparin/BA-ERRAT.png]

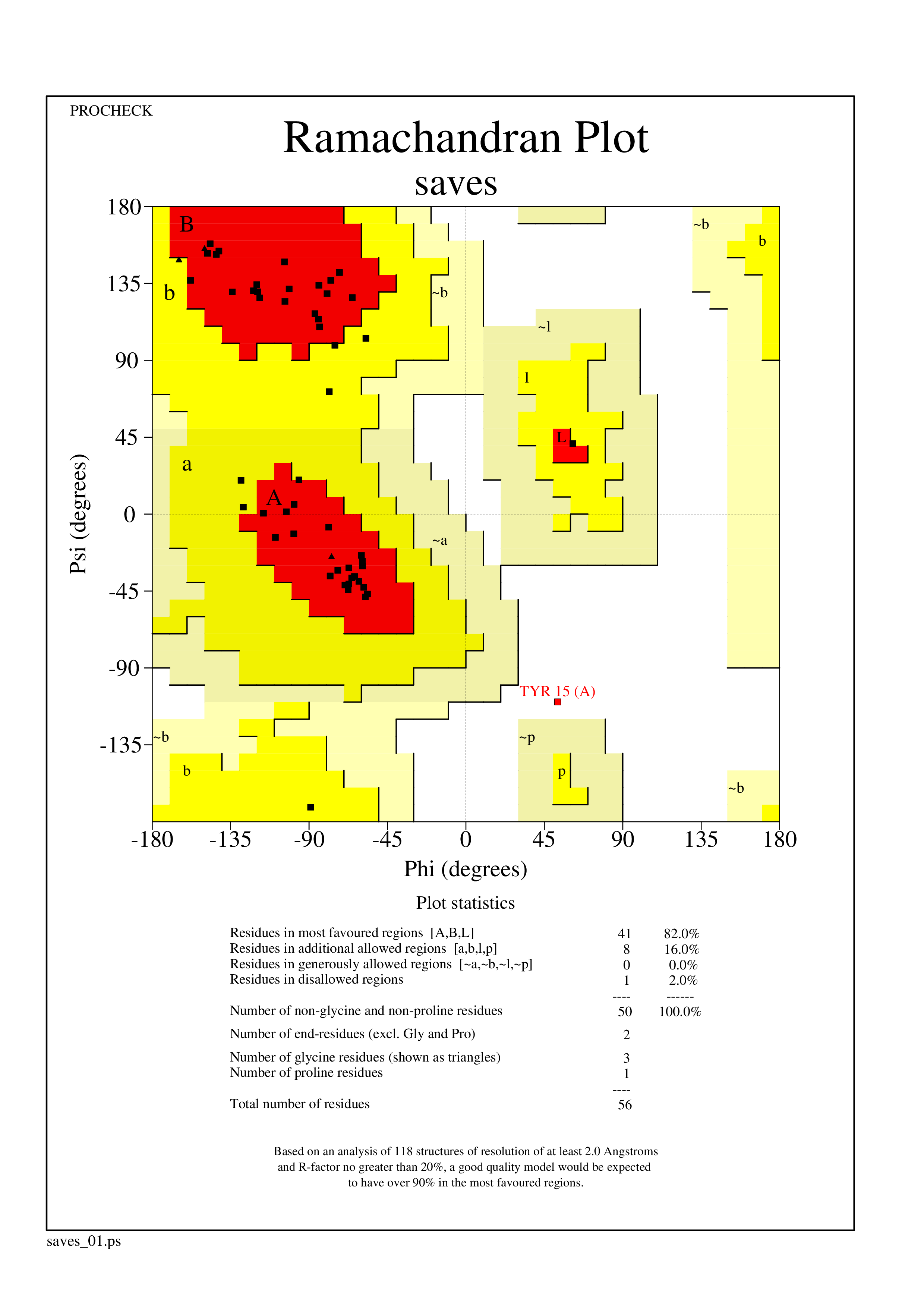

Supplement: Supplementary file 1 [file toxins-17-00262-s001.zip › Supplementary Material 2/File S12 Basparin/BA-PROCHE.png]

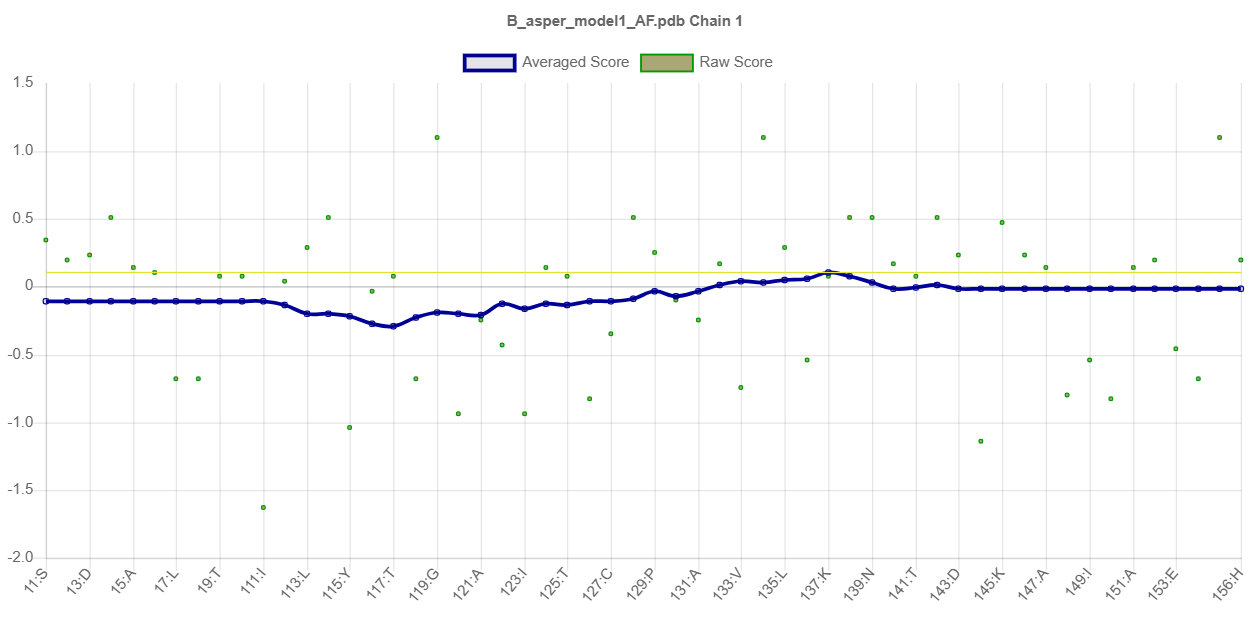

Supplement: Supplementary file 1 [file toxins-17-00262-s001.zip › Supplementary Material 2/File S12 Basparin/BA-VF3D.png]

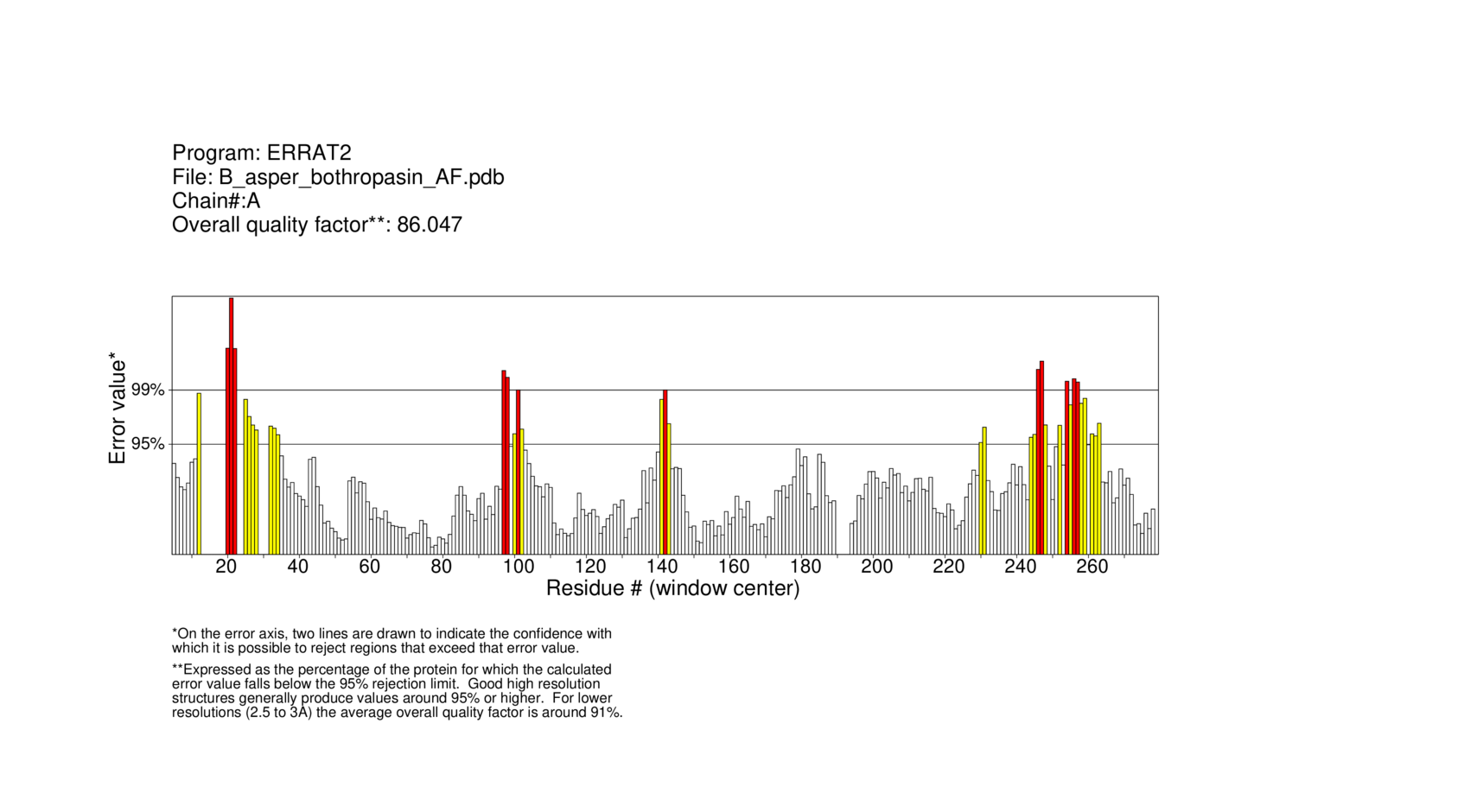

Supplement: Supplementary file 1 [file toxins-17-00262-s001.zip › Supplementary Material 2/File S12 Basparin/Bothropasin/BA-ERRAT-Bothropasin.png]

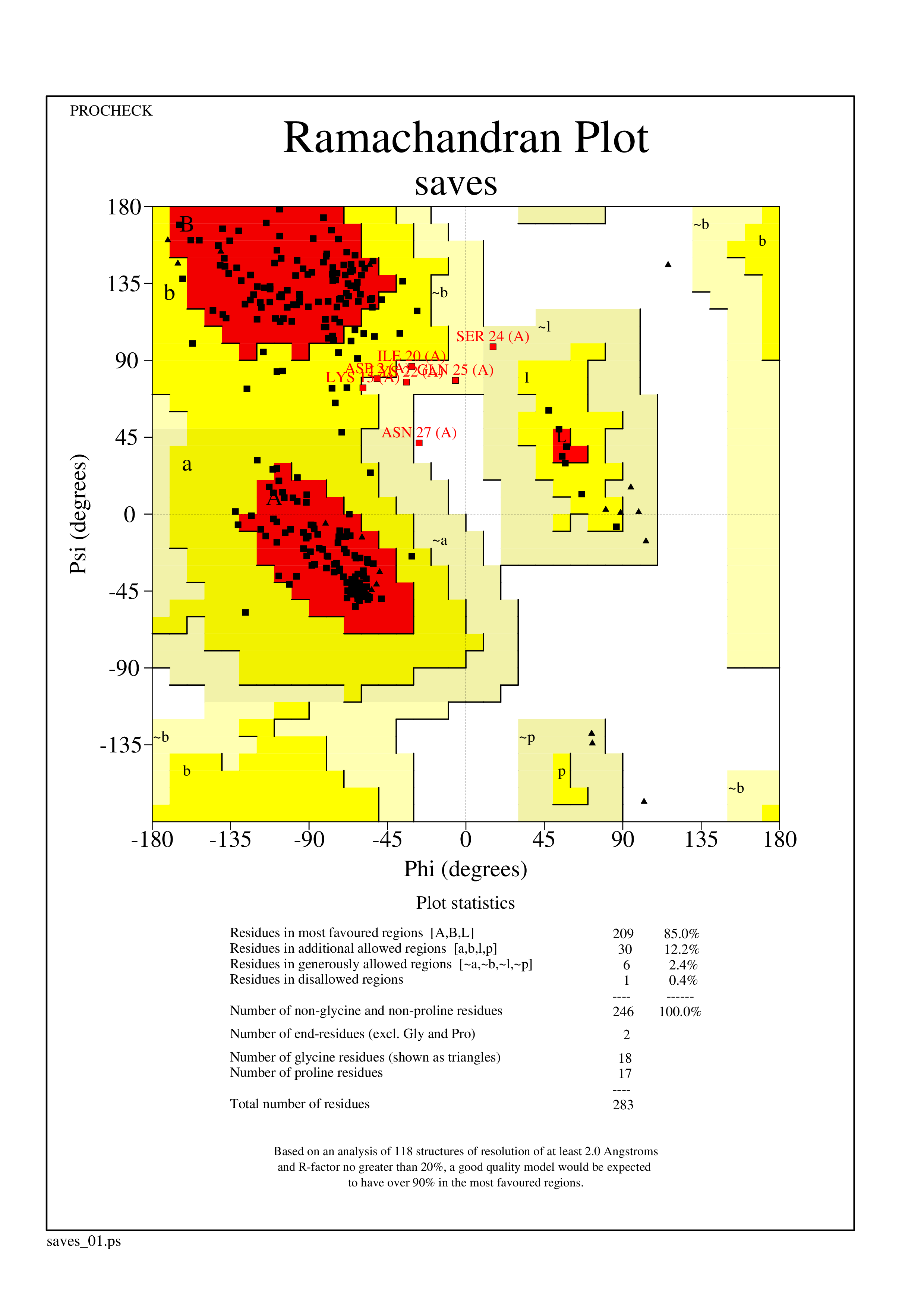

Supplement: Supplementary file 1 [file toxins-17-00262-s001.zip › Supplementary Material 2/File S12 Basparin/Bothropasin/BA-RAMACH-Bothropasin.png]

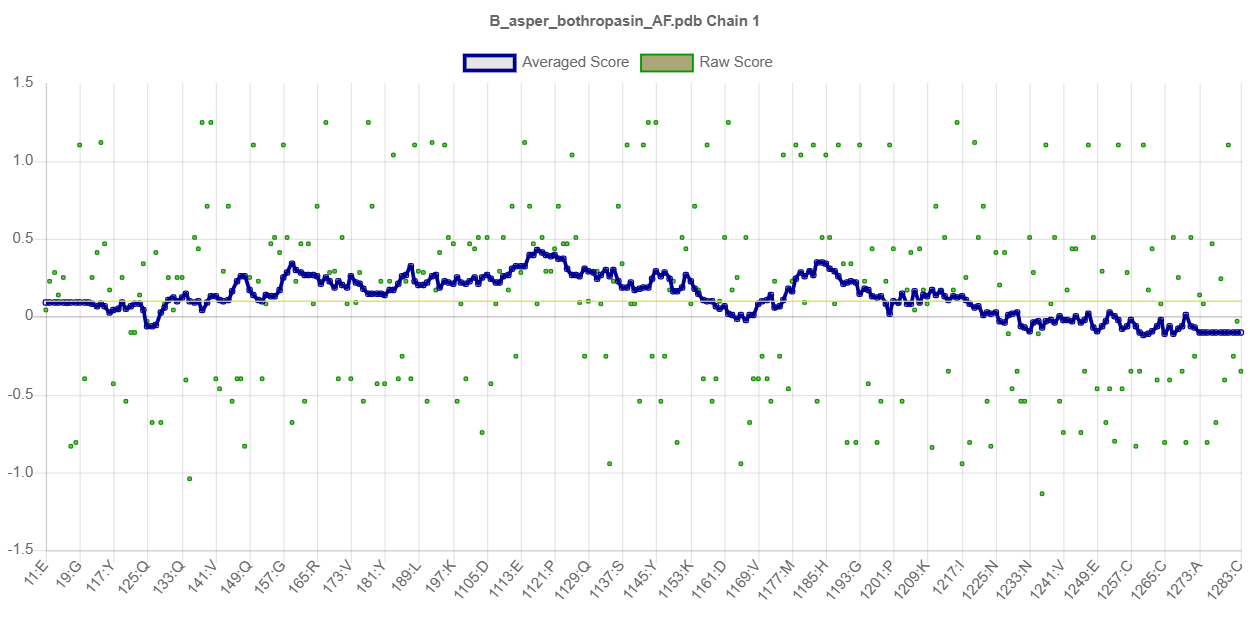

Supplement: Supplementary file 1 [file toxins-17-00262-s001.zip › Supplementary Material 2/File S12 Basparin/Bothropasin/BA-VERIFY3D-Bothropasin.png]

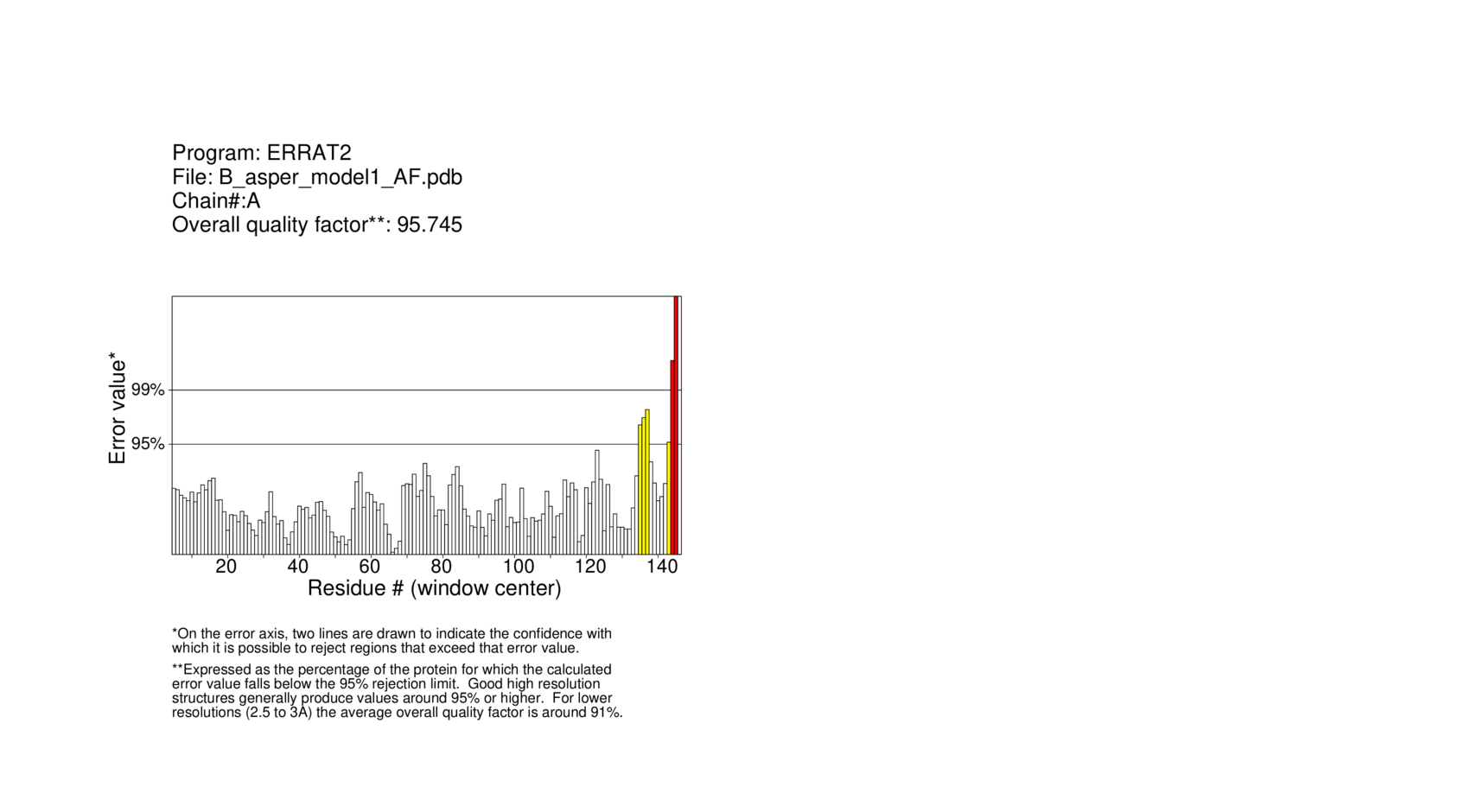

Supplement: Supplementary file 1 [file toxins-17-00262-s001.zip › Supplementary Material 2/File S7 CRISP/B.asper/BA-ERRAT-AG5.png]

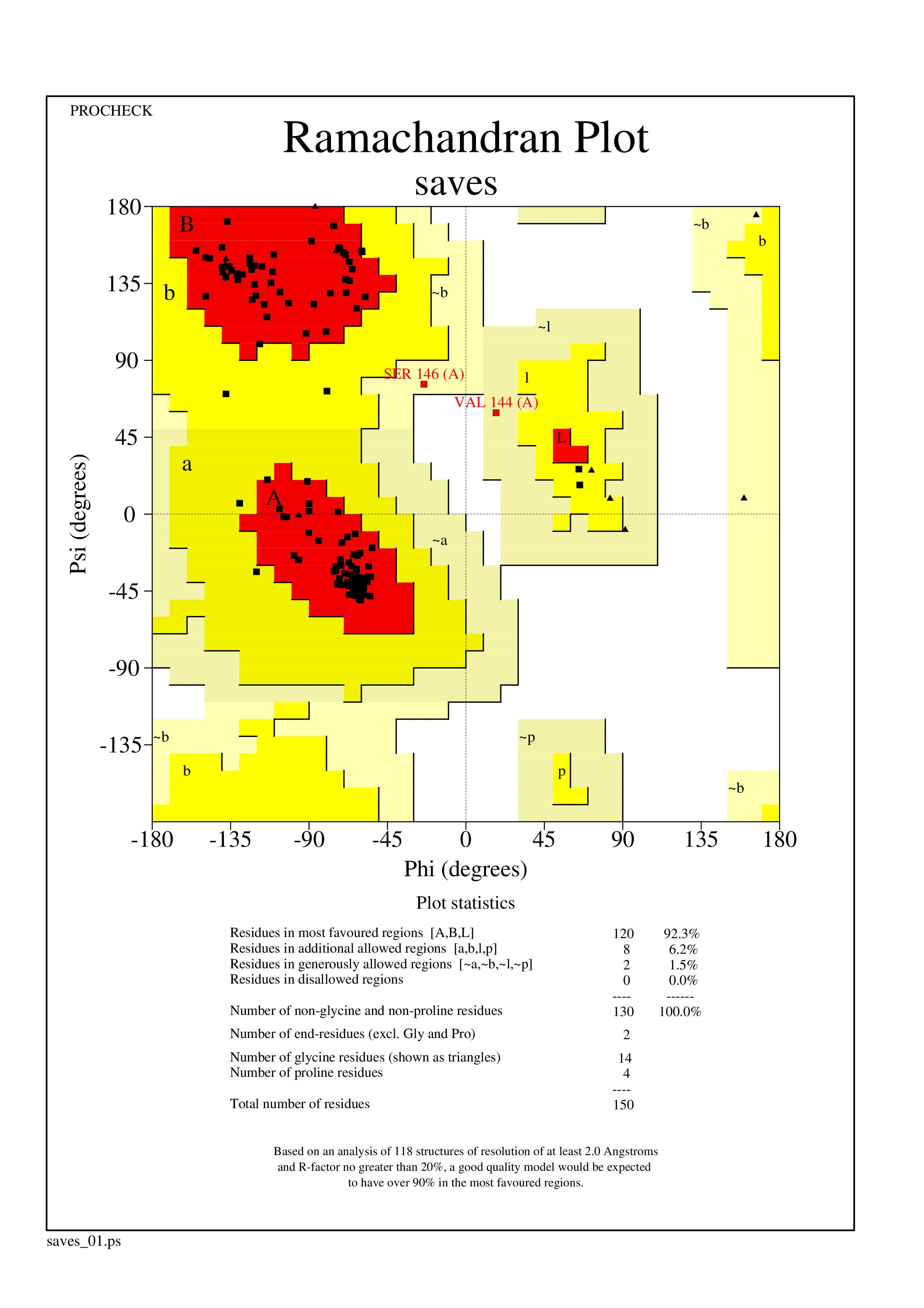

Supplement: Supplementary file 1 [file toxins-17-00262-s001.zip › Supplementary Material 2/File S7 CRISP/B.asper/BA-RAMACHANDRAN-AG5.png]

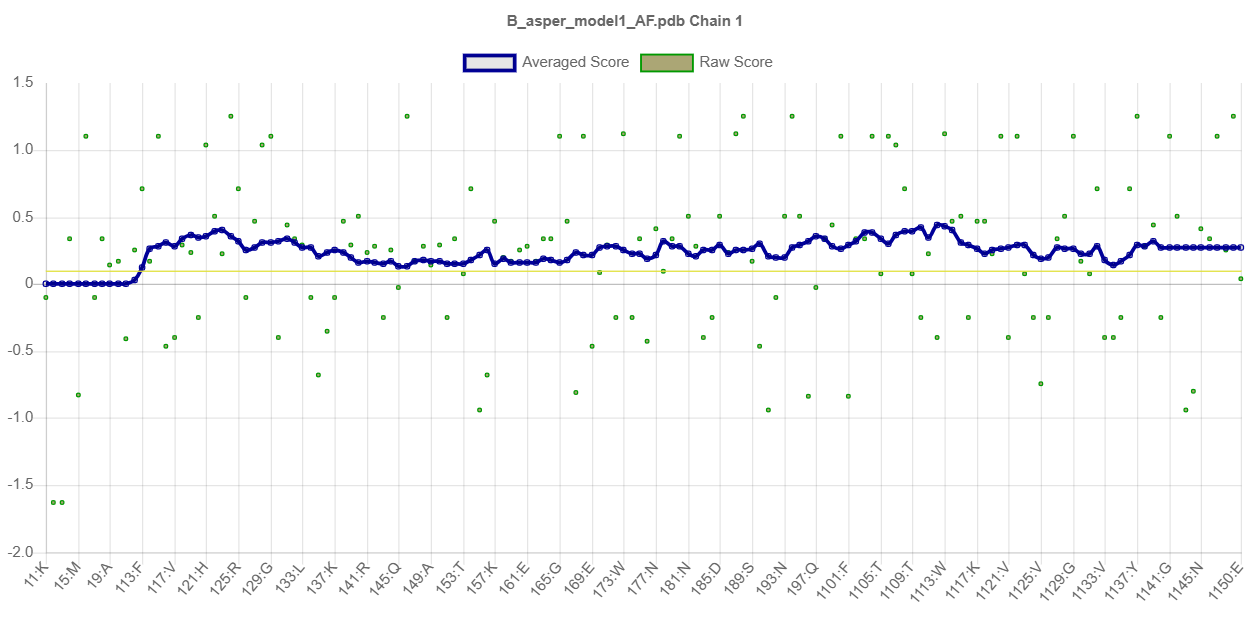

Supplement: Supplementary file 1 [file toxins-17-00262-s001.zip › Supplementary Material 2/File S7 CRISP/B.asper/BA-VERIFY3D-AG5.png]

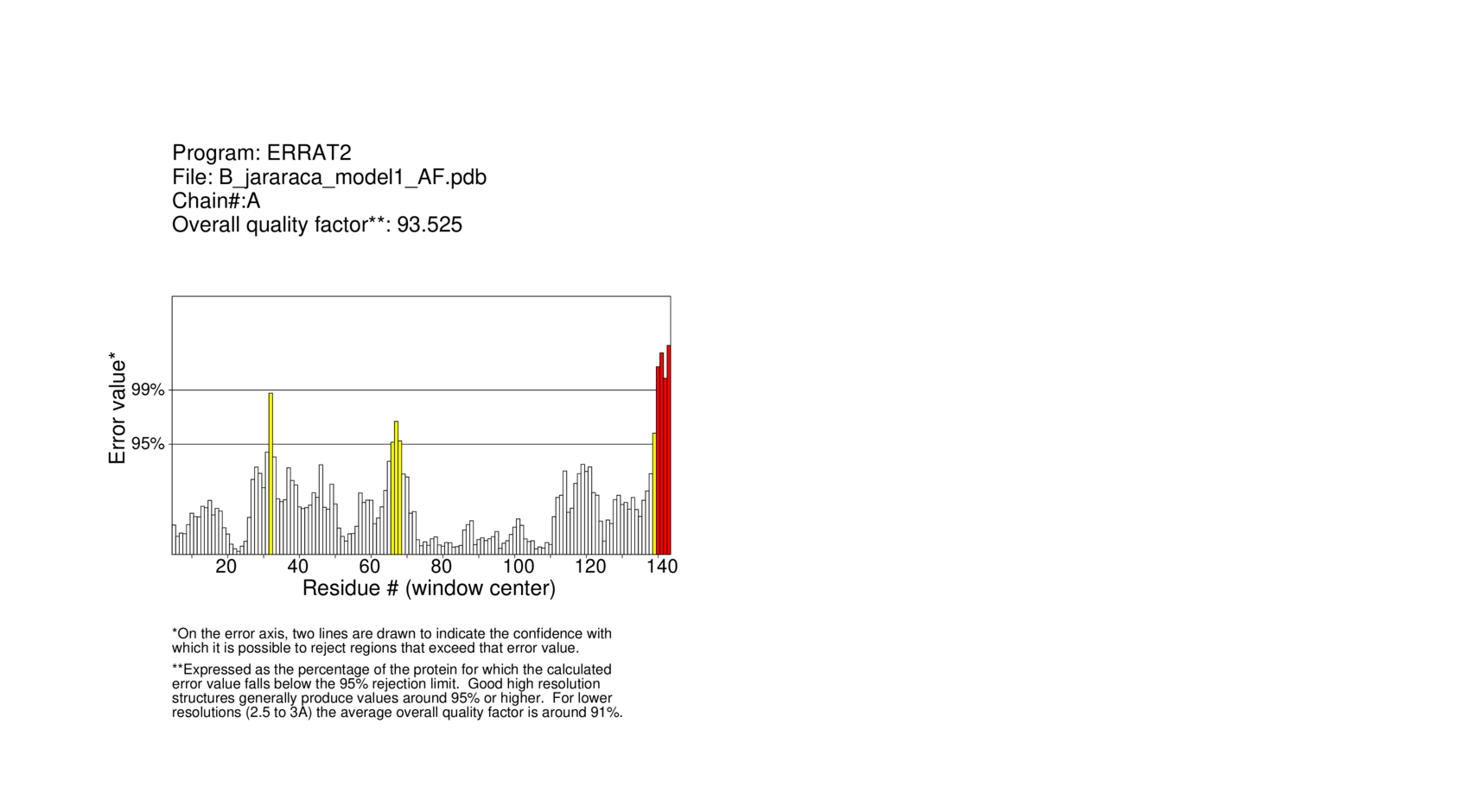

Supplement: Supplementary file 1 [file toxins-17-00262-s001.zip › Supplementary Material 2/File S7 CRISP/B.jararaca/BJ-ERRAT-AG5.png]

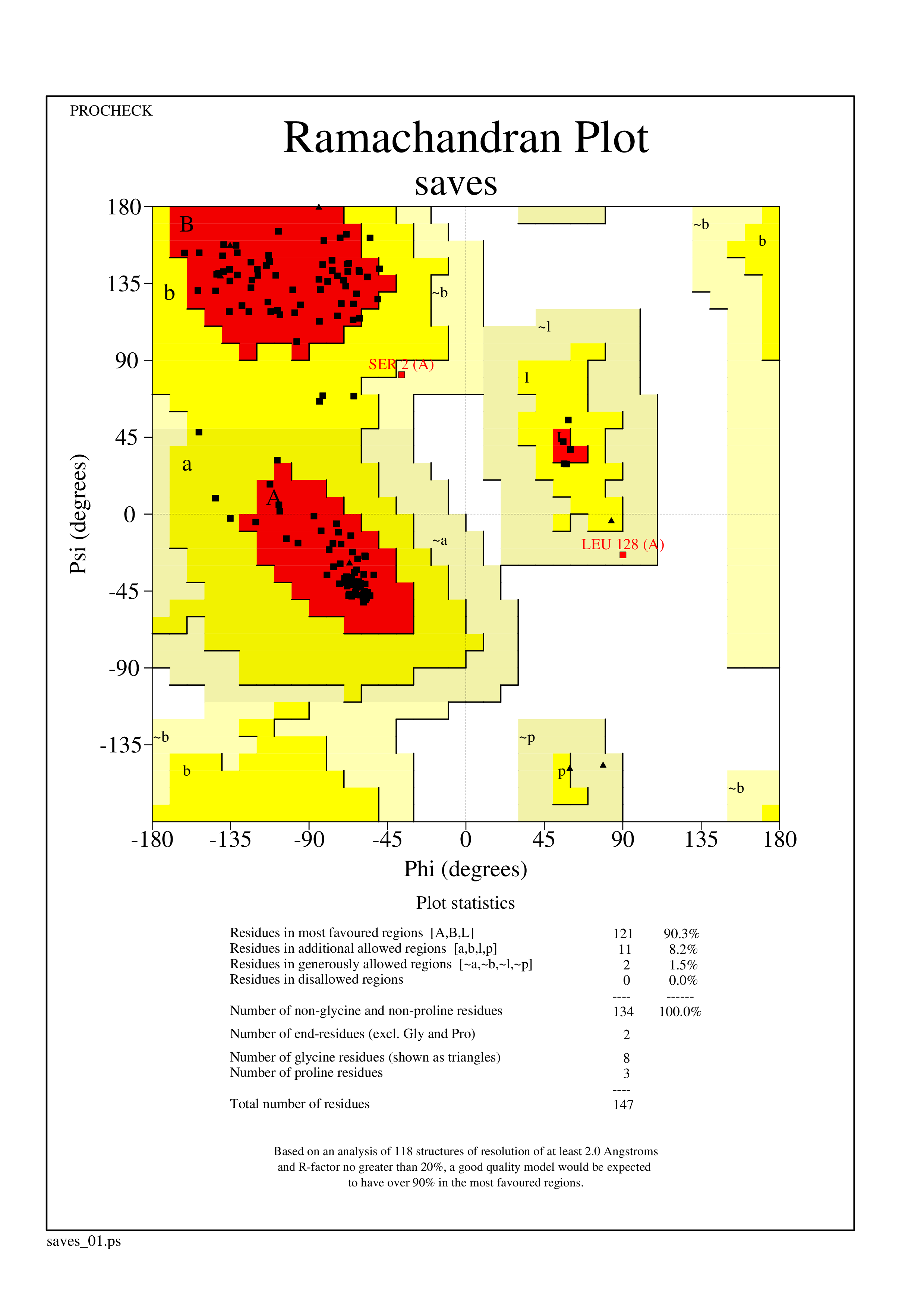

Supplement: Supplementary file 1 [file toxins-17-00262-s001.zip › Supplementary Material 2/File S7 CRISP/B.jararaca/BJ-RAMACHANDRAN-AG5.png]

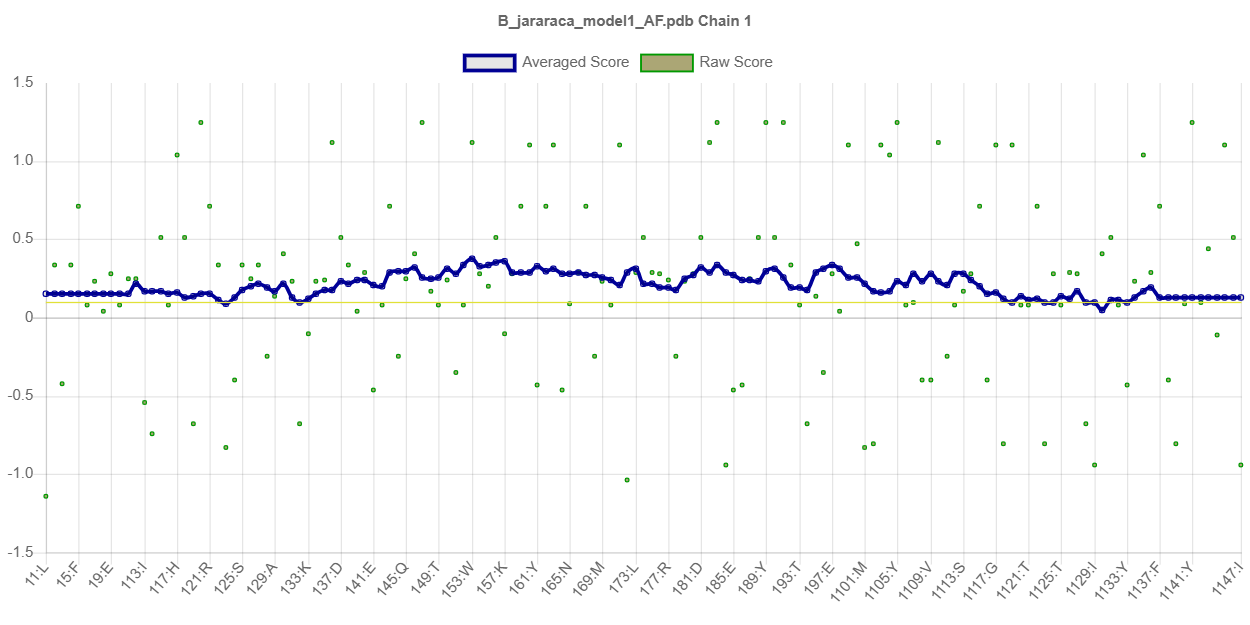

Supplement: Supplementary file 1 [file toxins-17-00262-s001.zip › Supplementary Material 2/File S7 CRISP/B.jararaca/BJ-VERIFY3D-AG5.png]

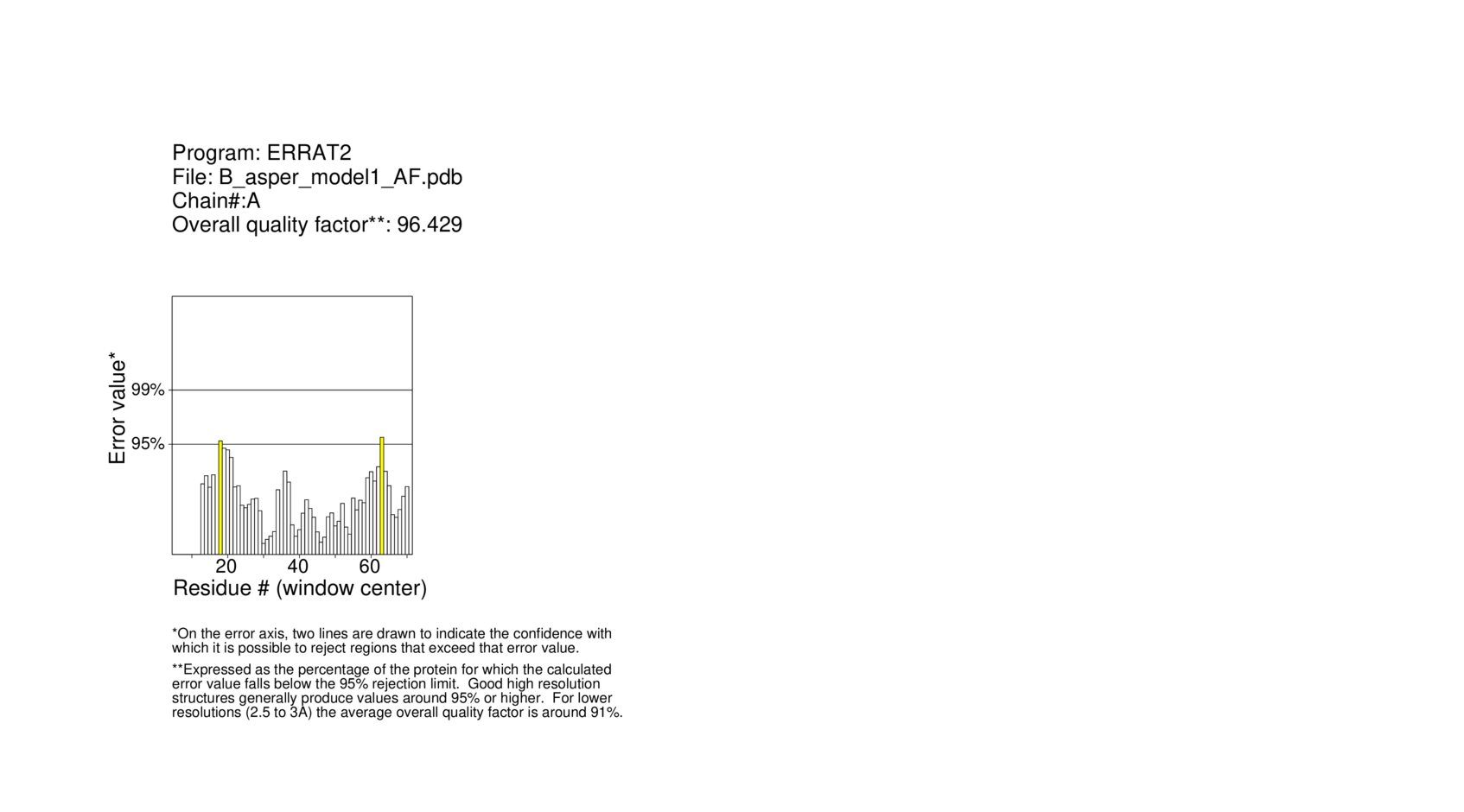

Supplement: Supplementary file 1 [file toxins-17-00262-s001.zip › Supplementary Material 2/File S8 Apolipophorin/B.asper/BA-ERRAT-APOLIPO.png]

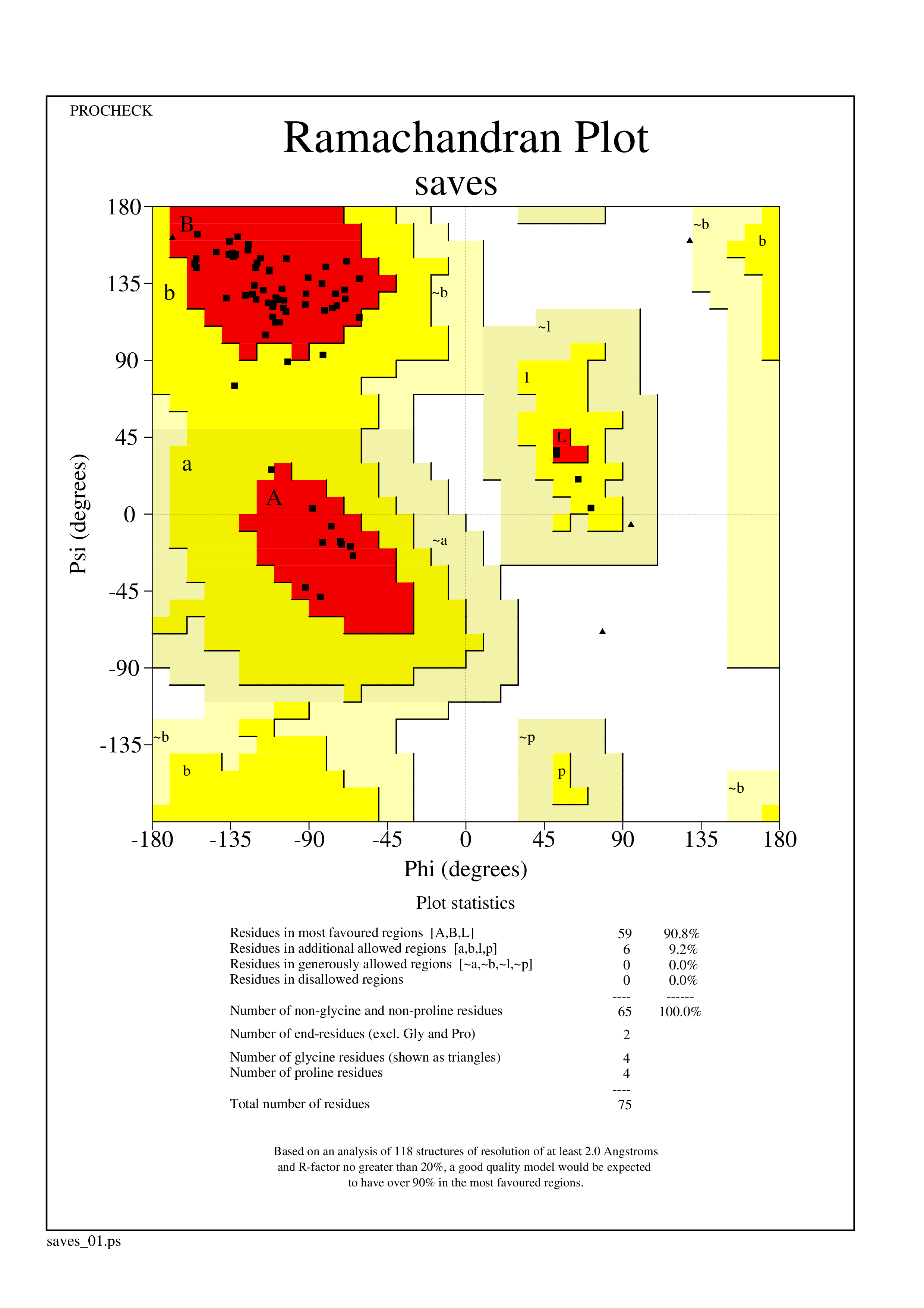

Supplement: Supplementary file 1 [file toxins-17-00262-s001.zip › Supplementary Material 2/File S8 Apolipophorin/B.asper/BA-RAMACH-APOLIPO.png]

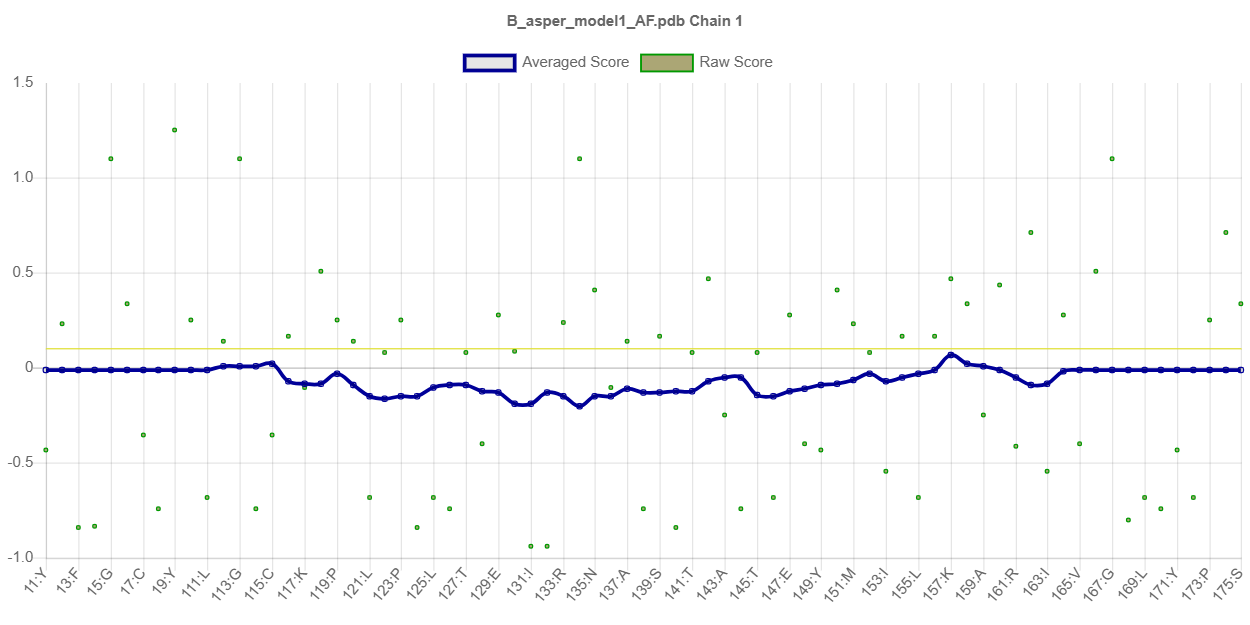

Supplement: Supplementary file 1 [file toxins-17-00262-s001.zip › Supplementary Material 2/File S8 Apolipophorin/B.asper/BA-VERIFY3D-APOLIPO.png]

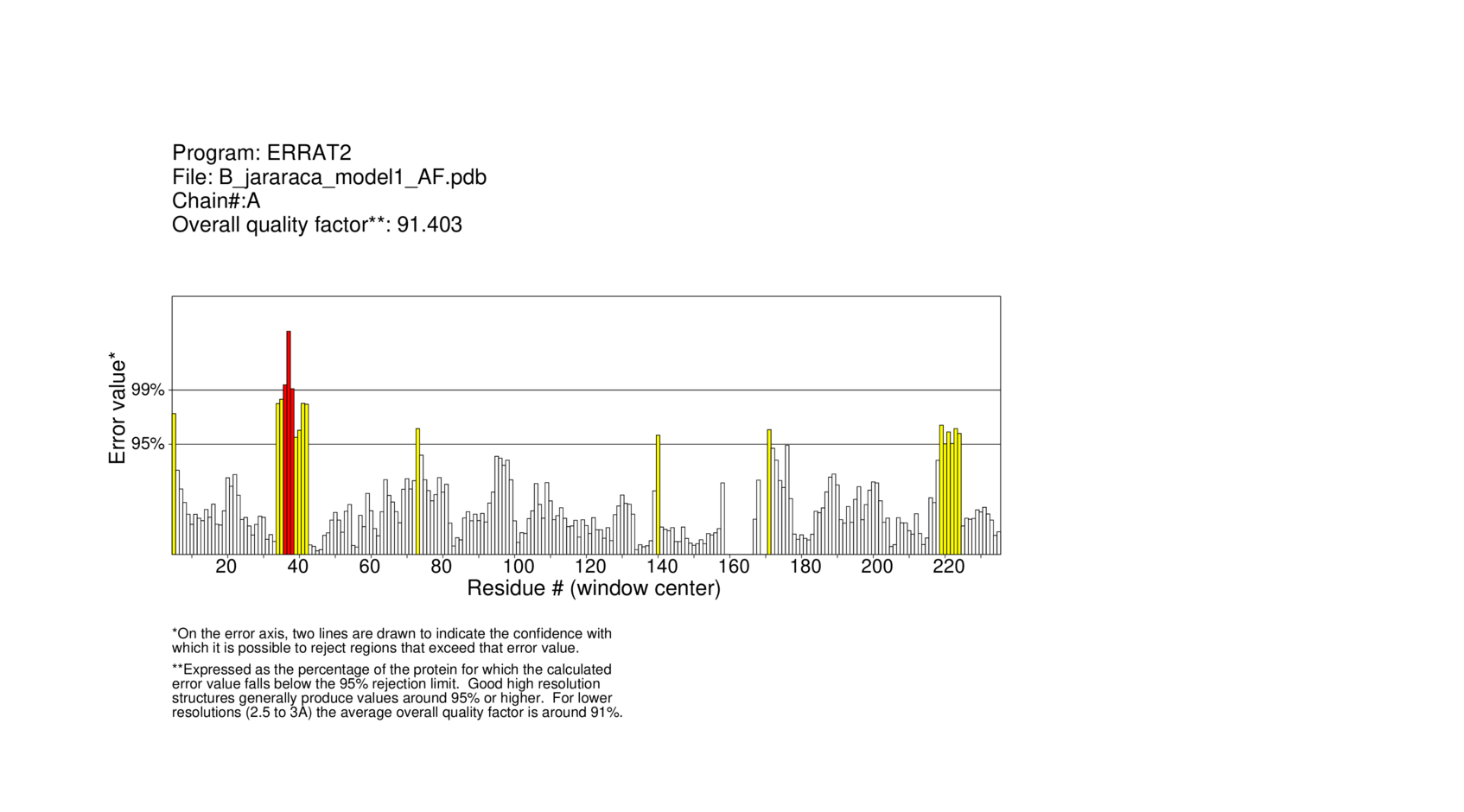

Supplement: Supplementary file 1 [file toxins-17-00262-s001.zip › Supplementary Material 2/File S8 Apolipophorin/B.jararaca/BJ-ERRAT-APOLIPO.png]

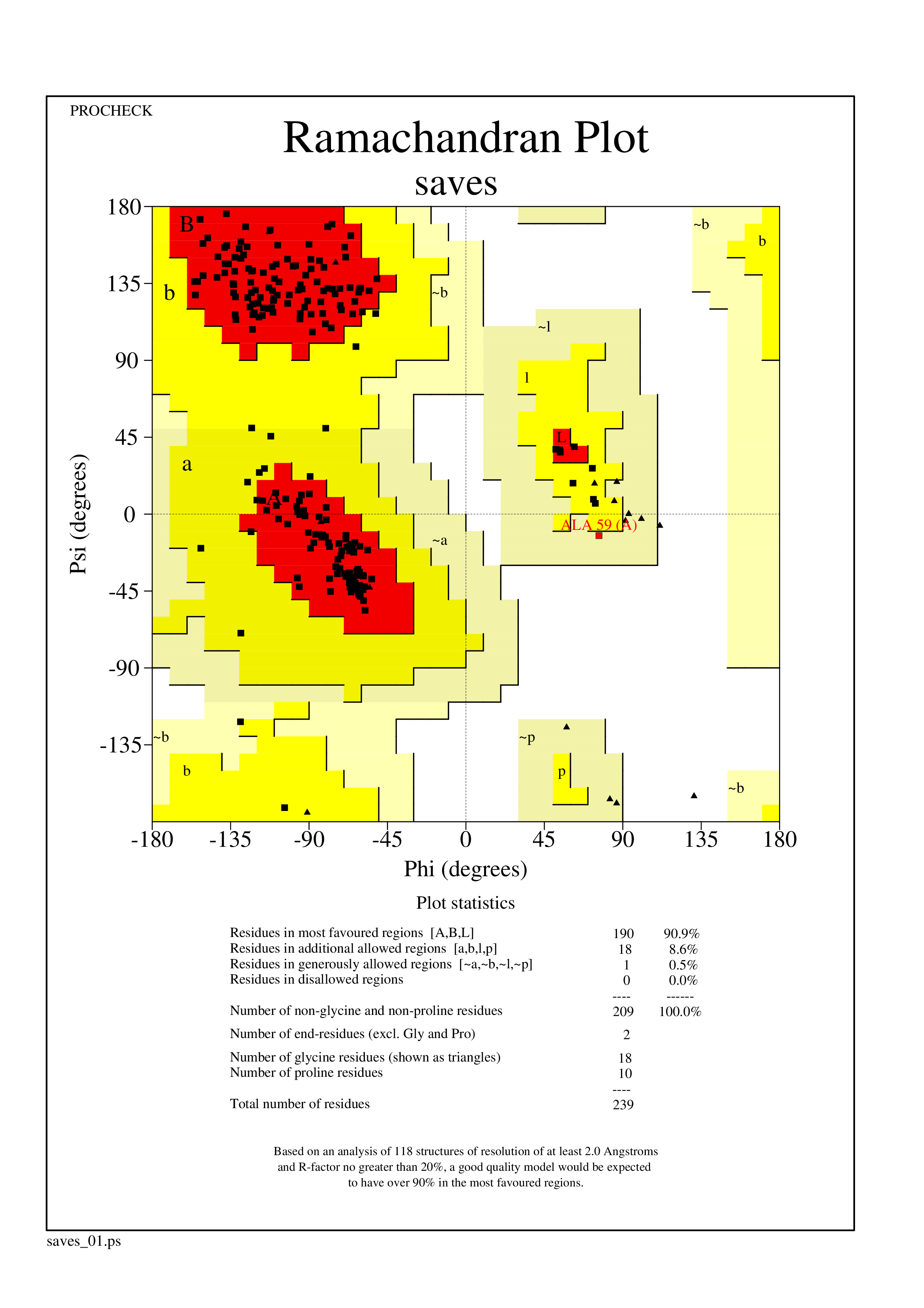

Supplement: Supplementary file 1 [file toxins-17-00262-s001.zip › Supplementary Material 2/File S8 Apolipophorin/B.jararaca/BJ-RAMACH-APOLIPO.png]

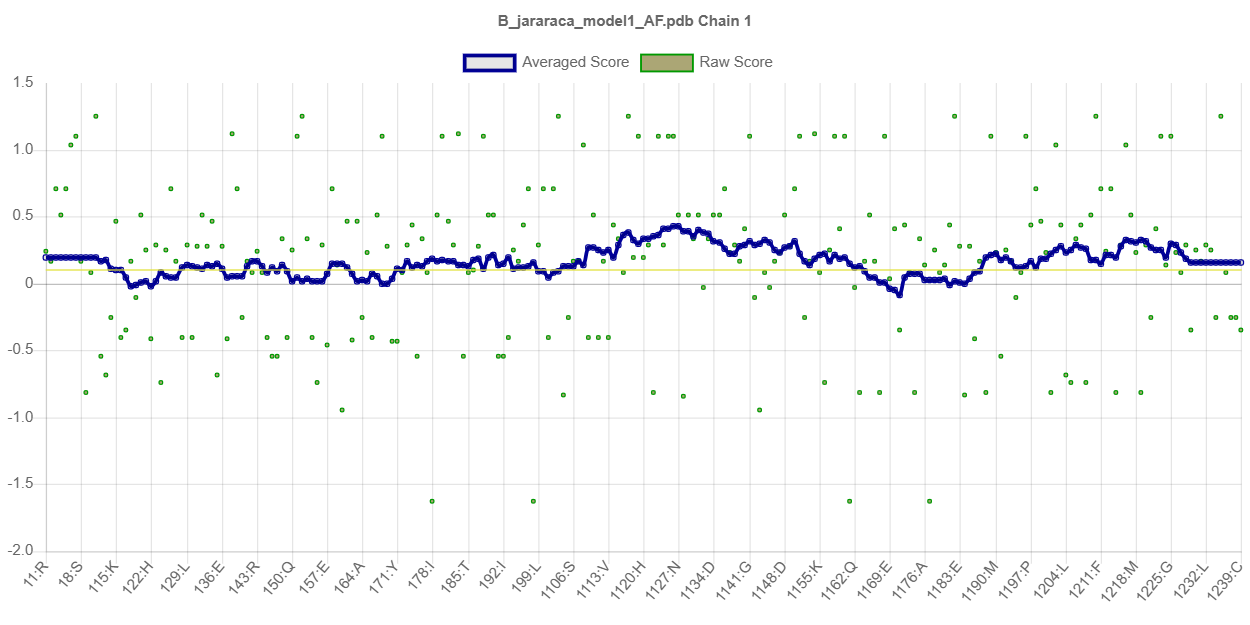

Supplement: Supplementary file 1 [file toxins-17-00262-s001.zip › Supplementary Material 2/File S8 Apolipophorin/B.jararaca/BJ-VERIFY3D-APOLIPO.png]

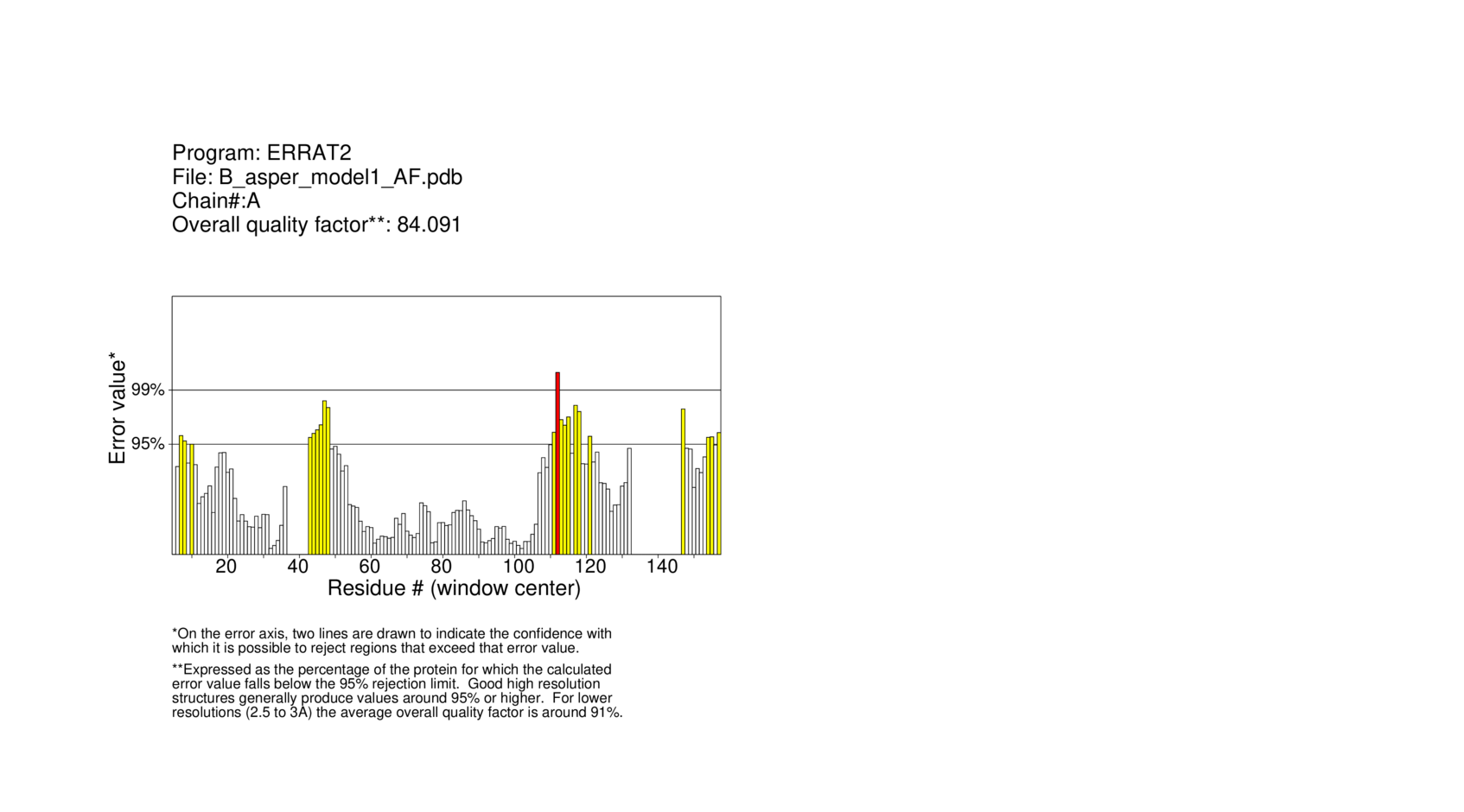

Supplement: Supplementary file 1 [file toxins-17-00262-s001.zip › Supplementary Material 2/File S8 Apolipophorin/vWFD/B.asper/BA-ERRAT.png]

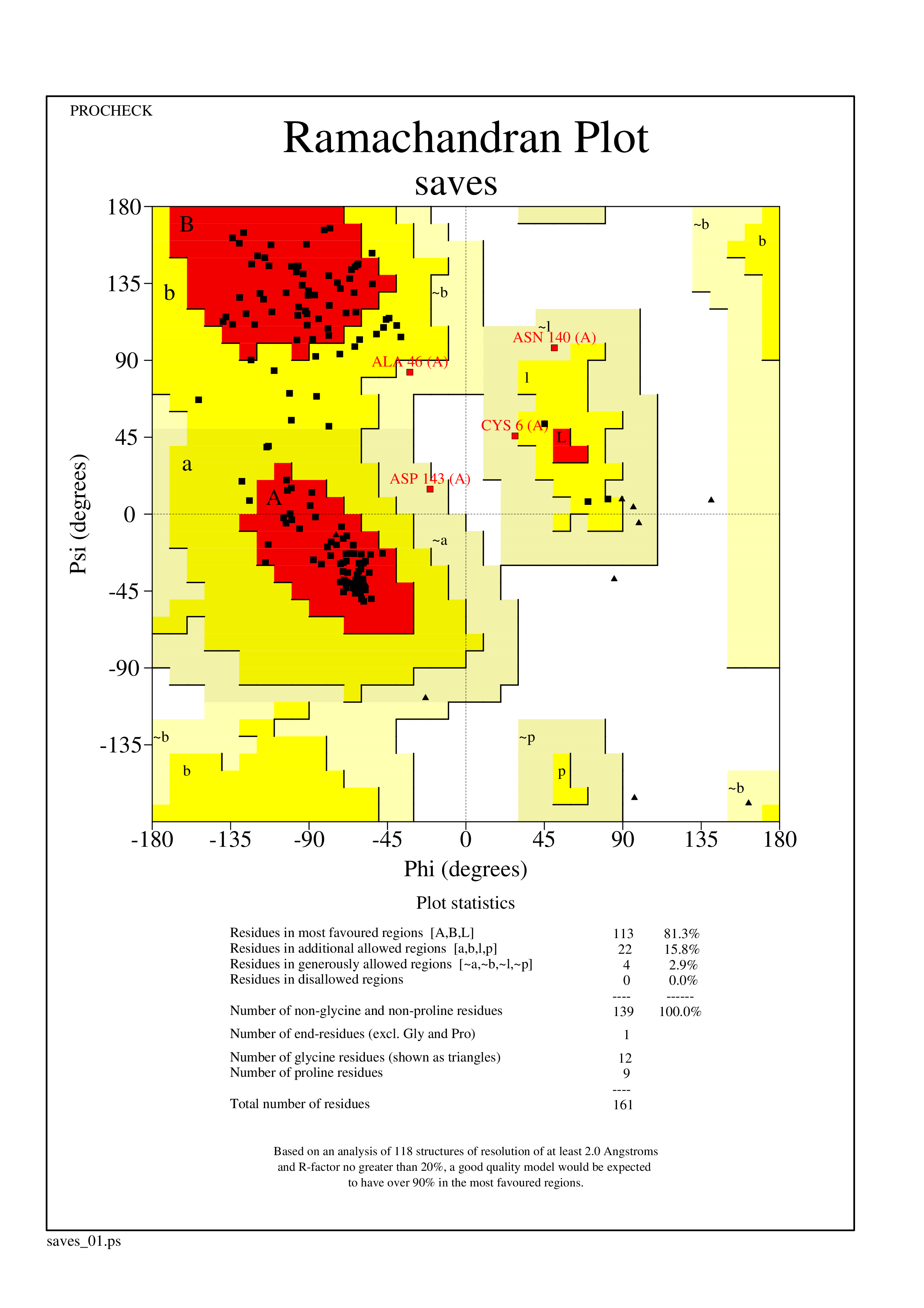

Supplement: Supplementary file 1 [file toxins-17-00262-s001.zip › Supplementary Material 2/File S8 Apolipophorin/vWFD/B.asper/BA-RAMACH.png]

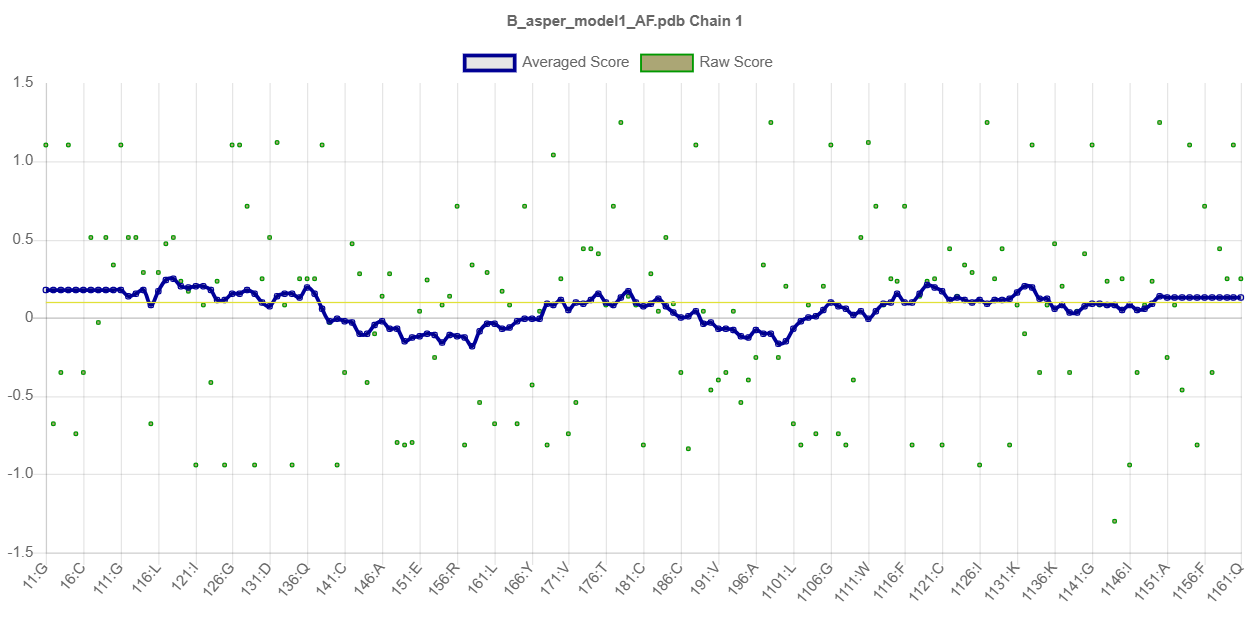

Supplement: Supplementary file 1 [file toxins-17-00262-s001.zip › Supplementary Material 2/File S8 Apolipophorin/vWFD/B.asper/BA-VF3D.png]

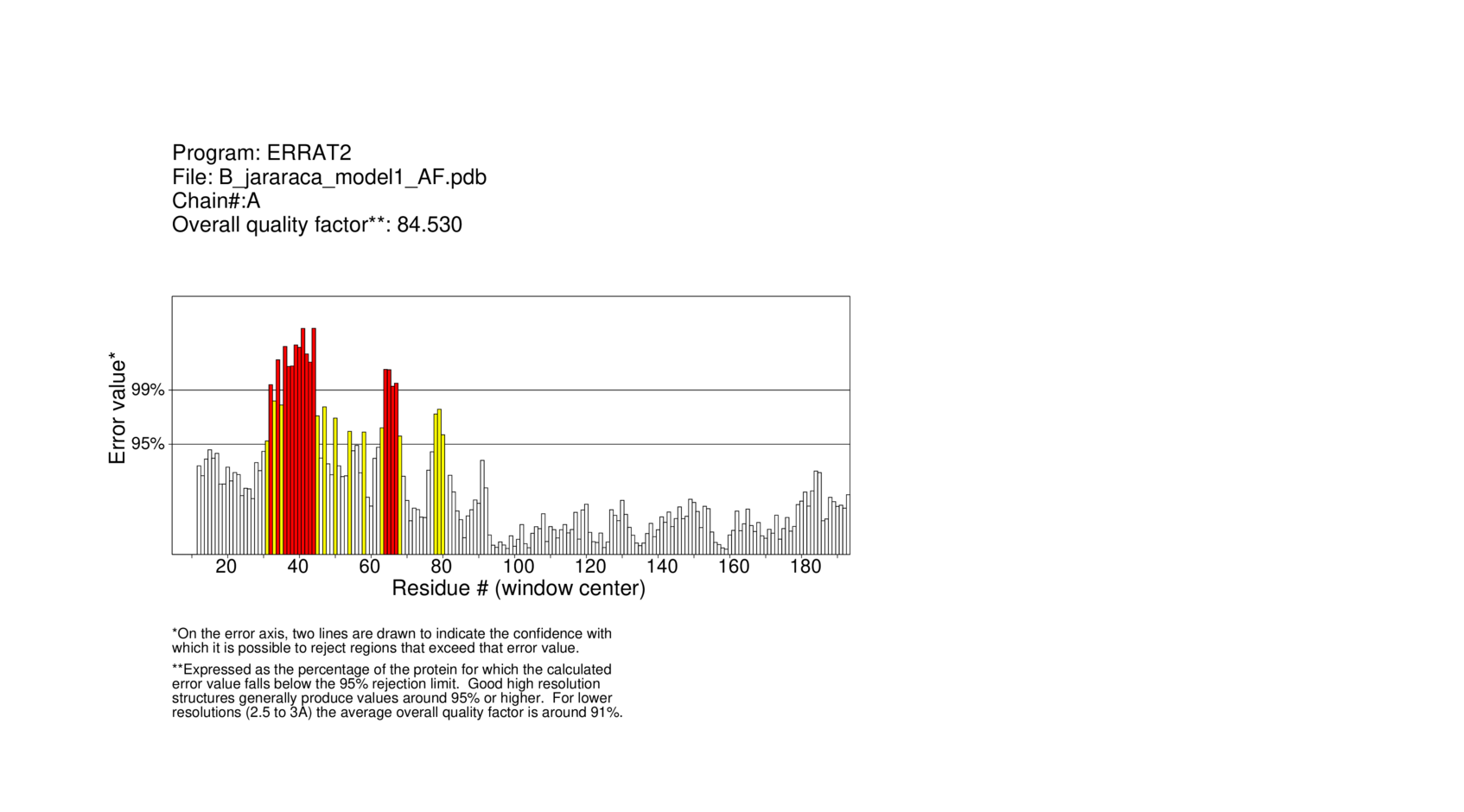

Supplement: Supplementary file 1 [file toxins-17-00262-s001.zip › Supplementary Material 2/File S8 Apolipophorin/vWFD/B.jararaca/BJ-ERRAT.png]

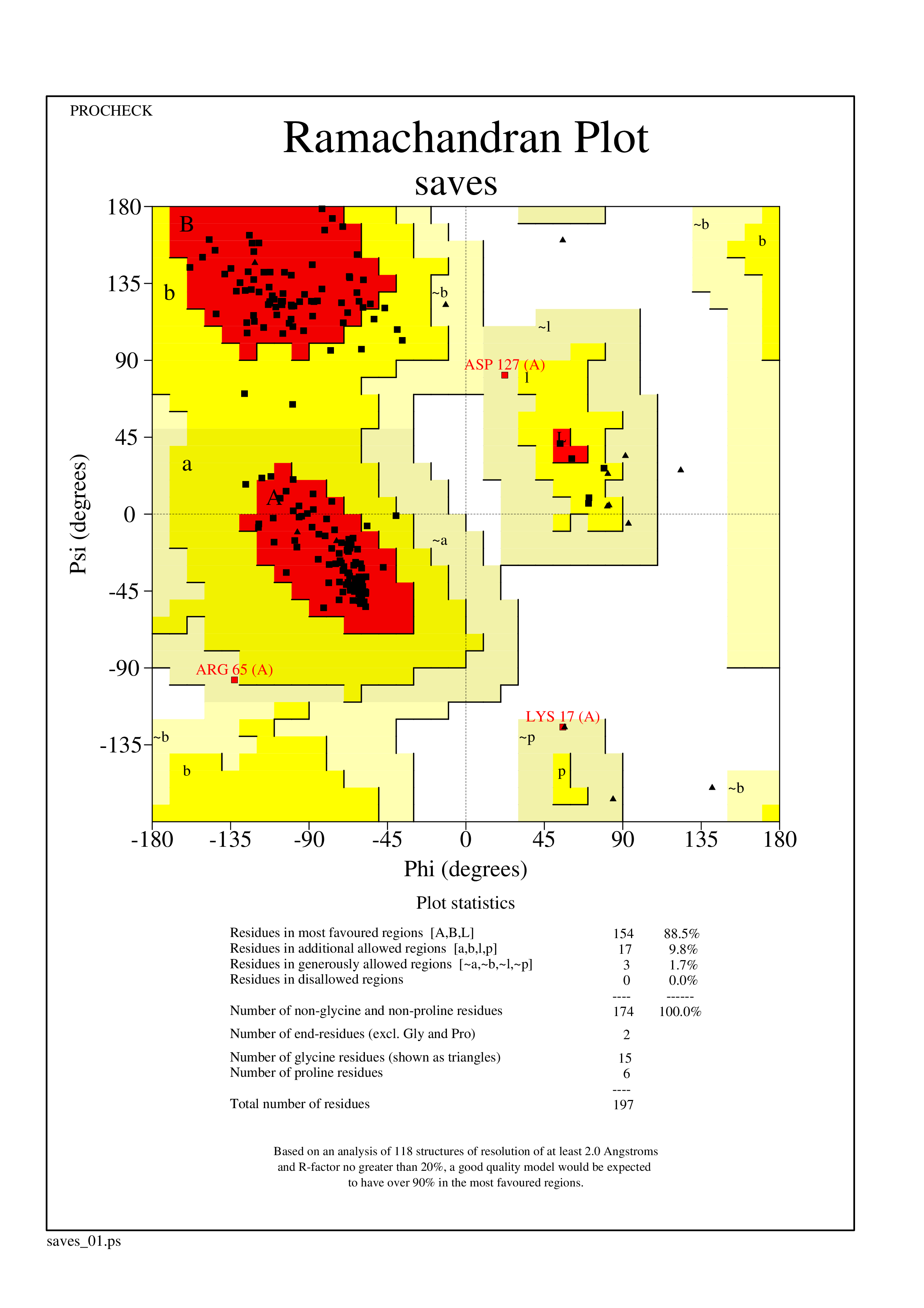

Supplement: Supplementary file 1 [file toxins-17-00262-s001.zip › Supplementary Material 2/File S8 Apolipophorin/vWFD/B.jararaca/BJ-RAMACH.png]

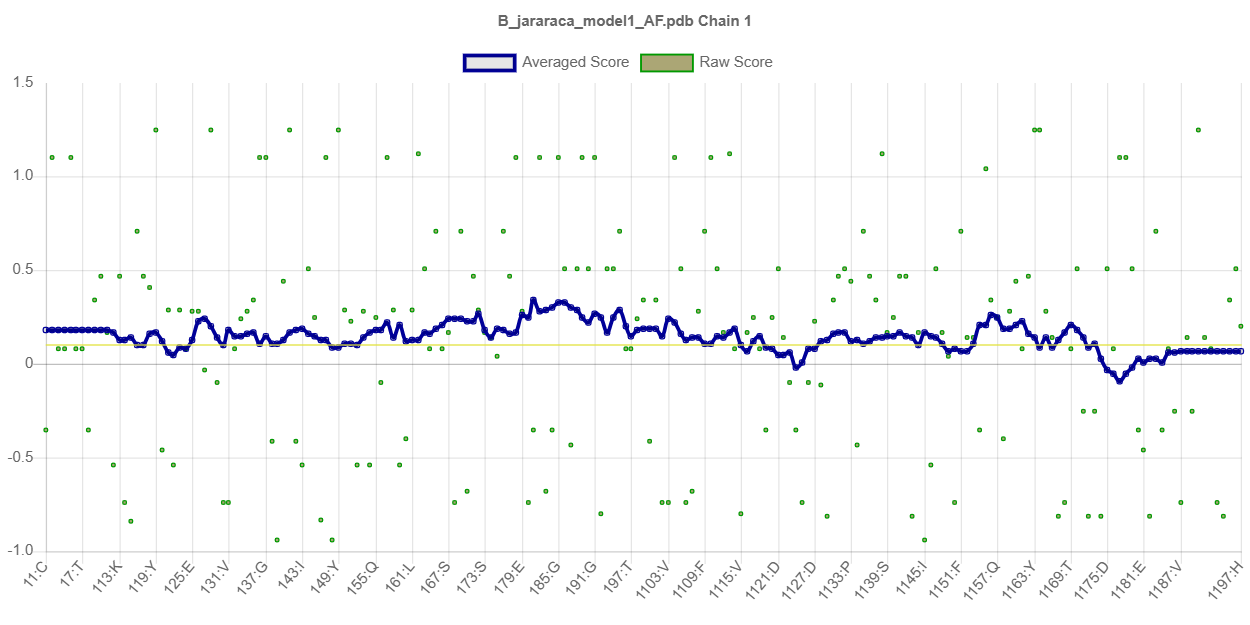

Supplement: Supplementary file 1 [file toxins-17-00262-s001.zip › Supplementary Material 2/File S8 Apolipophorin/vWFD/B.jararaca/BJ-VF3D.png]

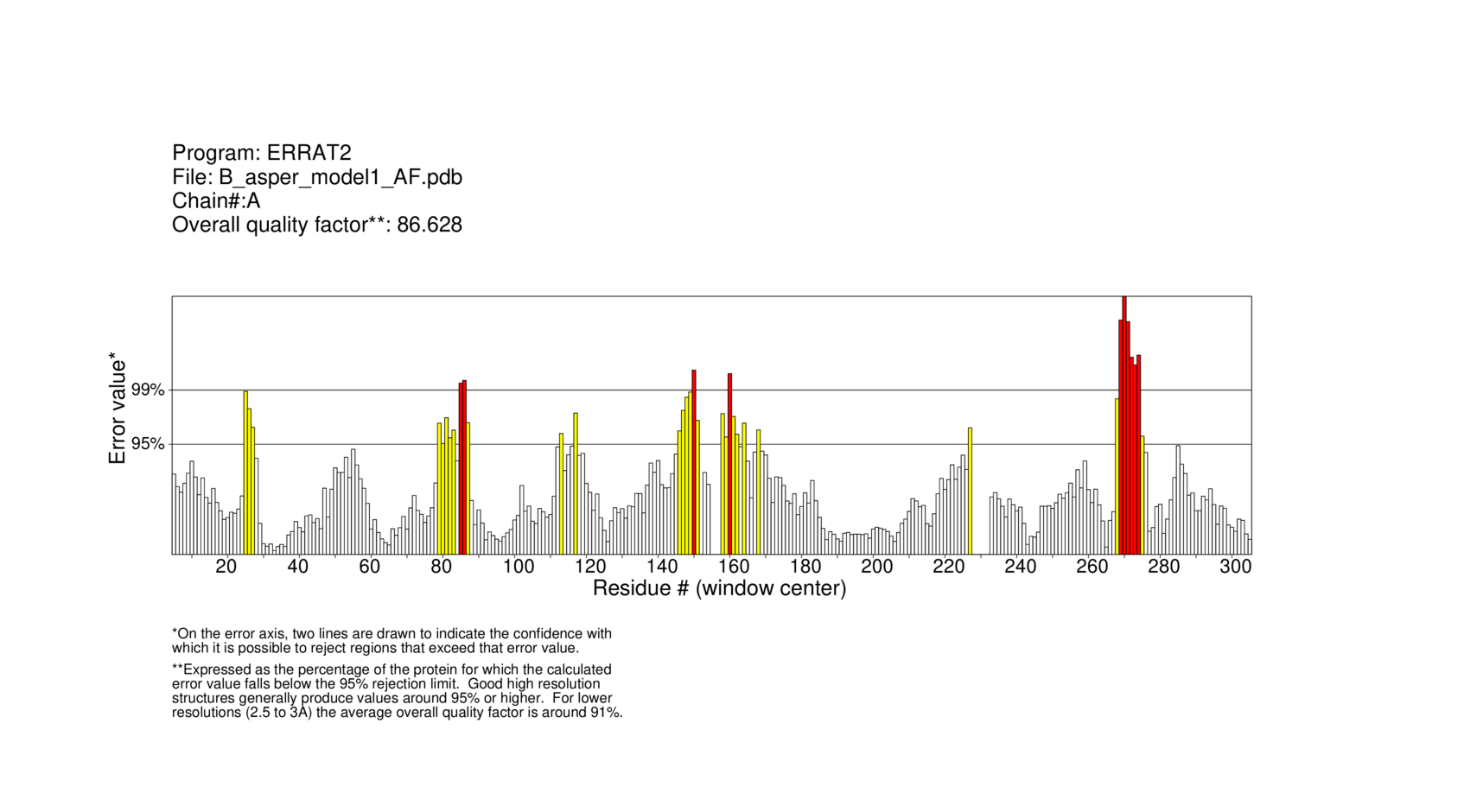

Supplement: Supplementary file 1 [file toxins-17-00262-s001.zip › Supplementary Material 2/File S9 ARSB/B.asper/BA-ERRAT.png]

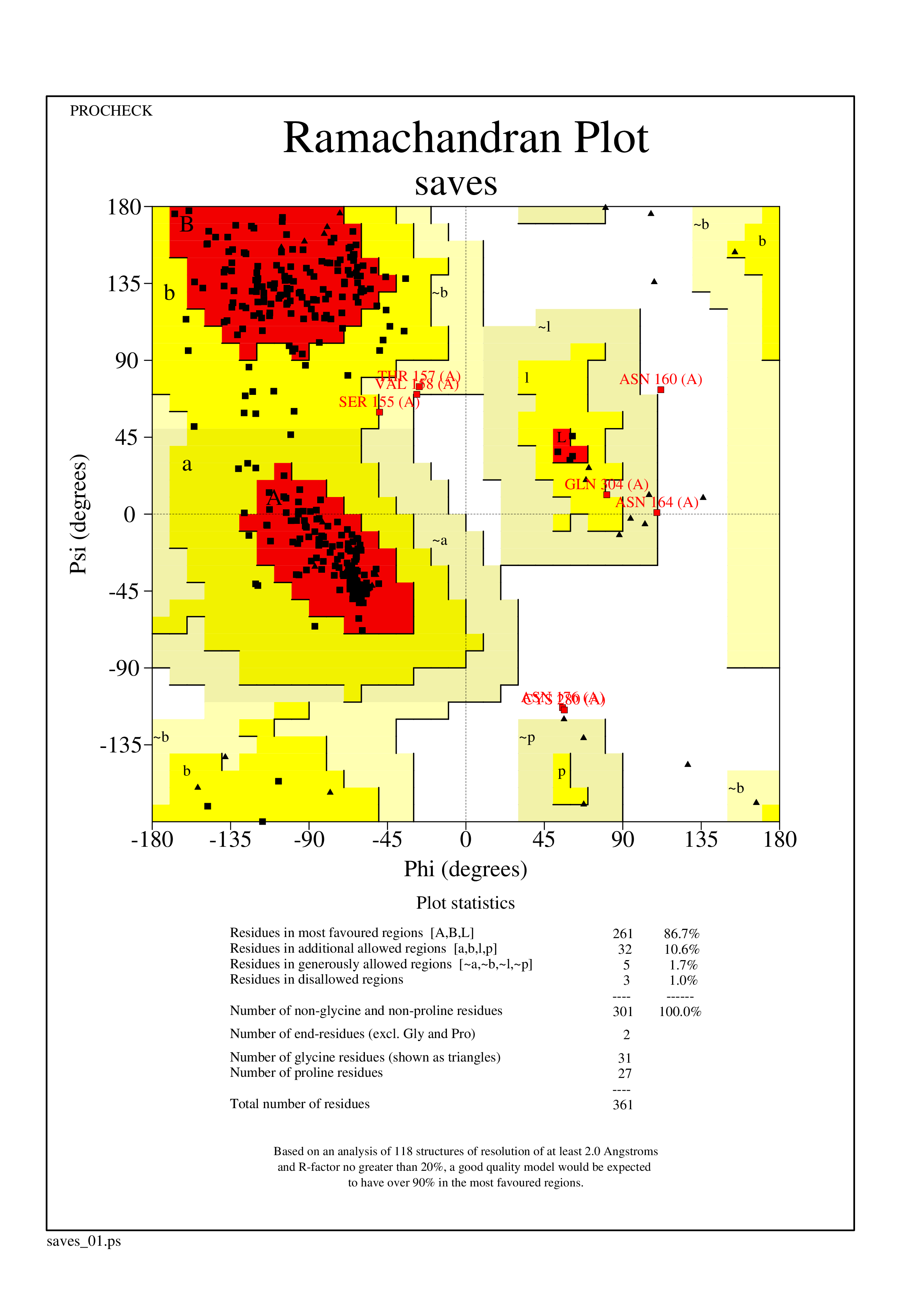

Supplement: Supplementary file 1 [file toxins-17-00262-s001.zip › Supplementary Material 2/File S9 ARSB/B.asper/BA-RAMACH.png]

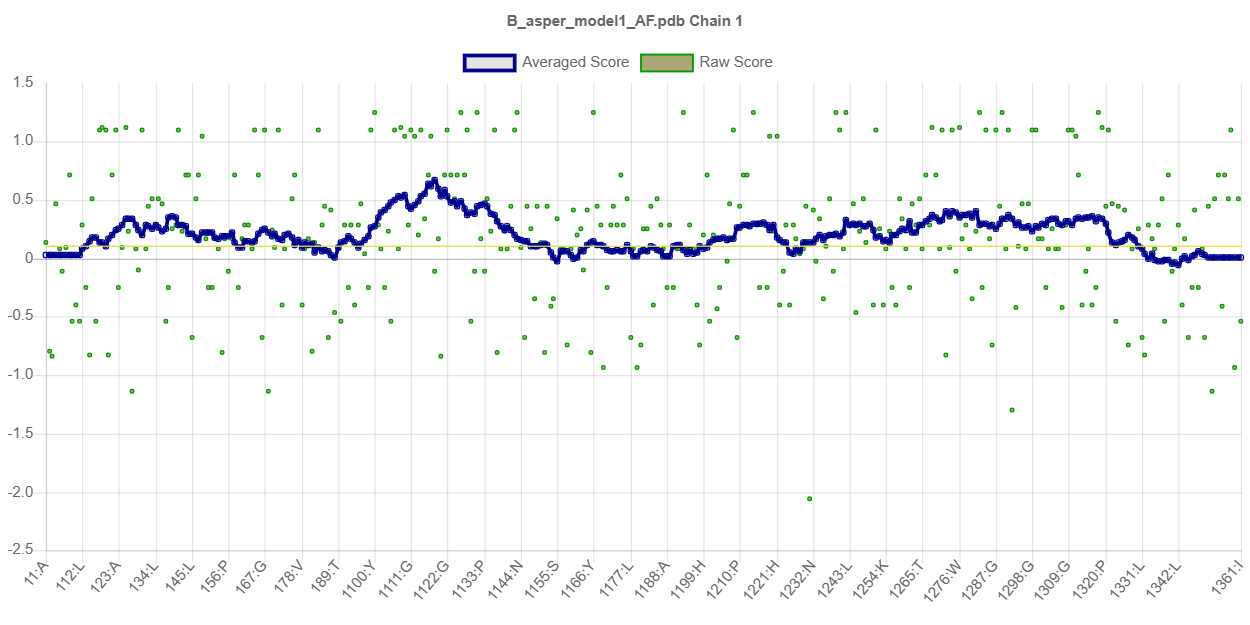

Supplement: Supplementary file 1 [file toxins-17-00262-s001.zip › Supplementary Material 2/File S9 ARSB/B.asper/BA-VF3D.png]

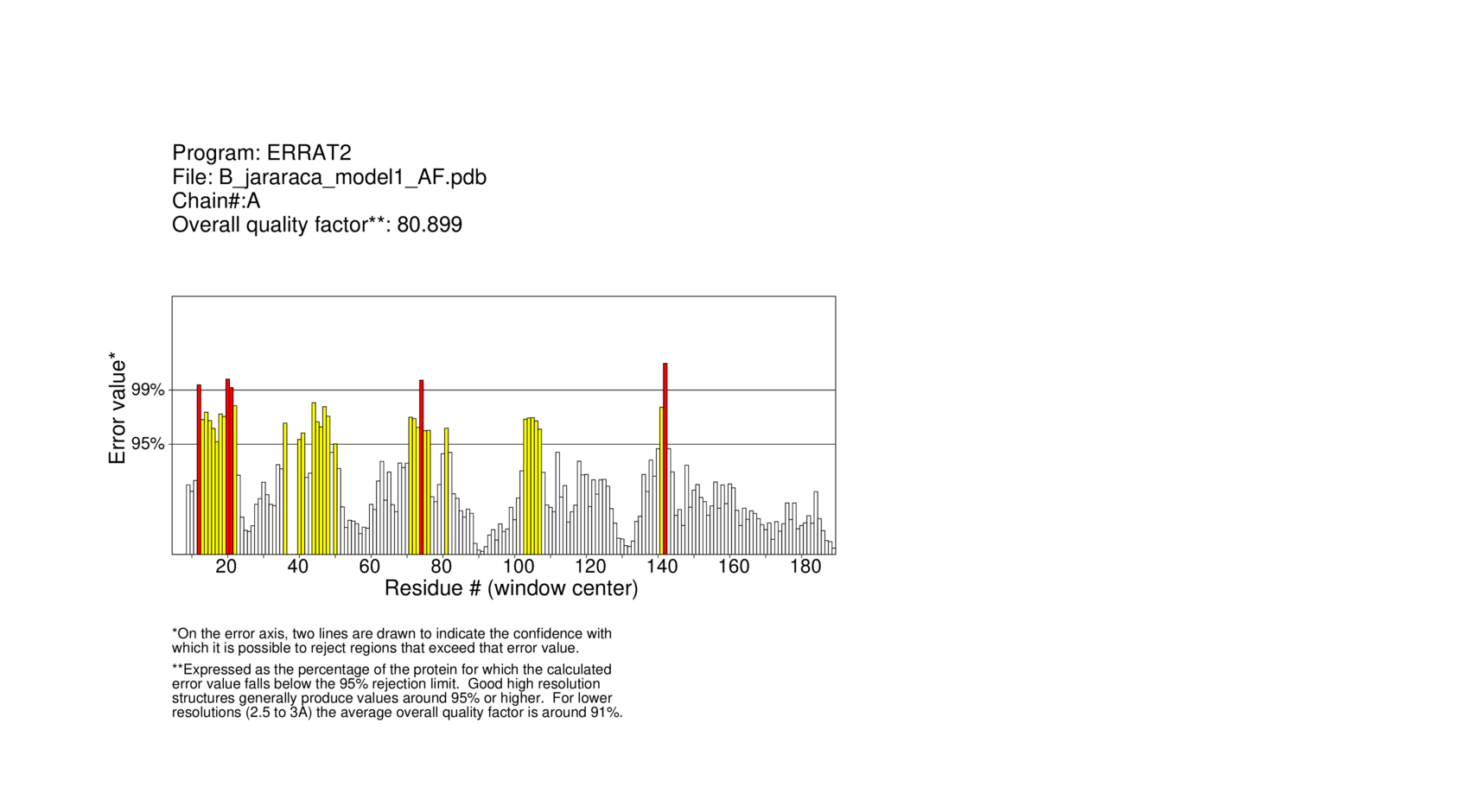

Supplement: Supplementary file 1 [file toxins-17-00262-s001.zip › Supplementary Material 2/File S9 ARSB/B.jararaca/BJ-ERRAT.png]

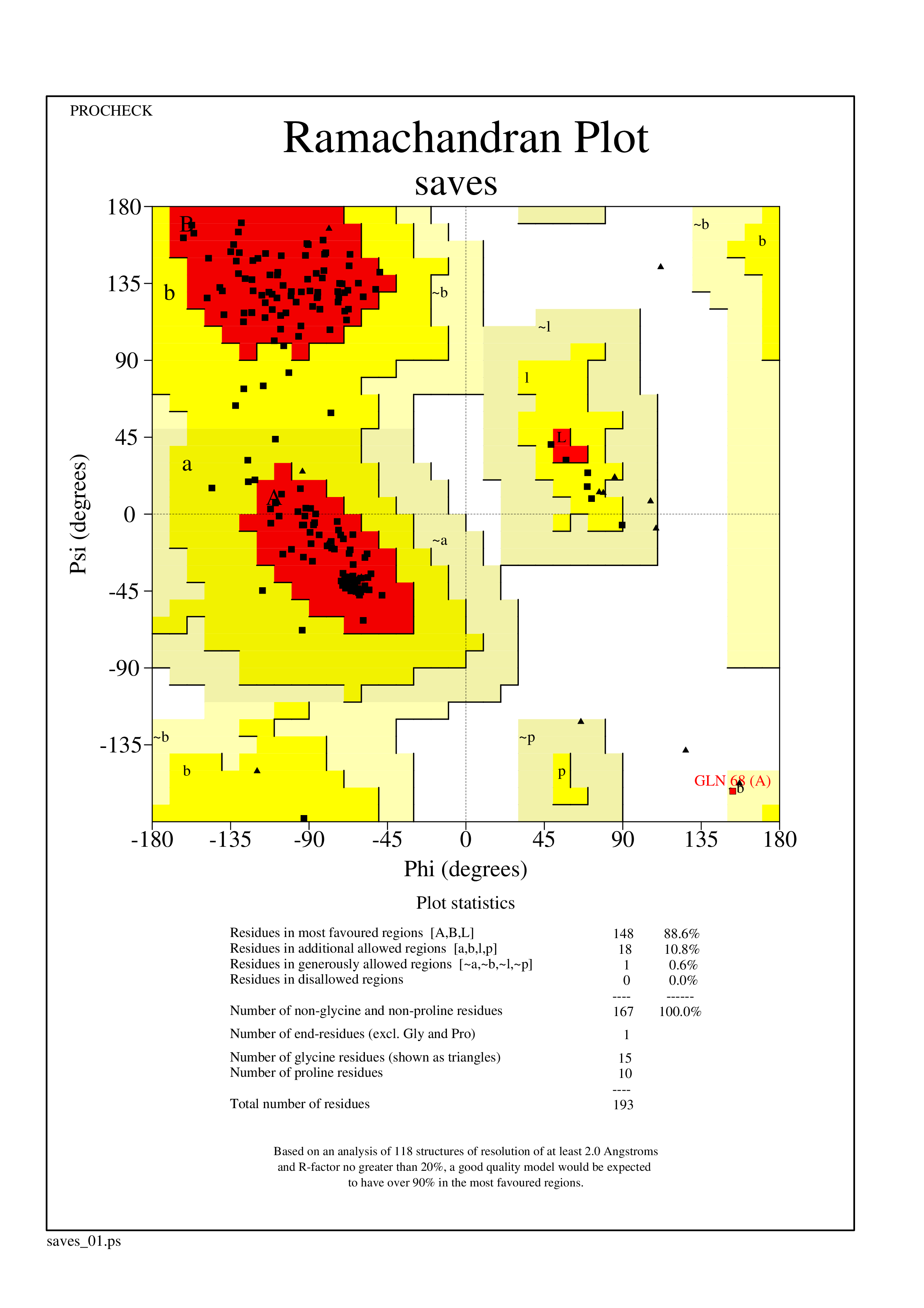

Supplement: Supplementary file 1 [file toxins-17-00262-s001.zip › Supplementary Material 2/File S9 ARSB/B.jararaca/BJ-PROCHE.png]

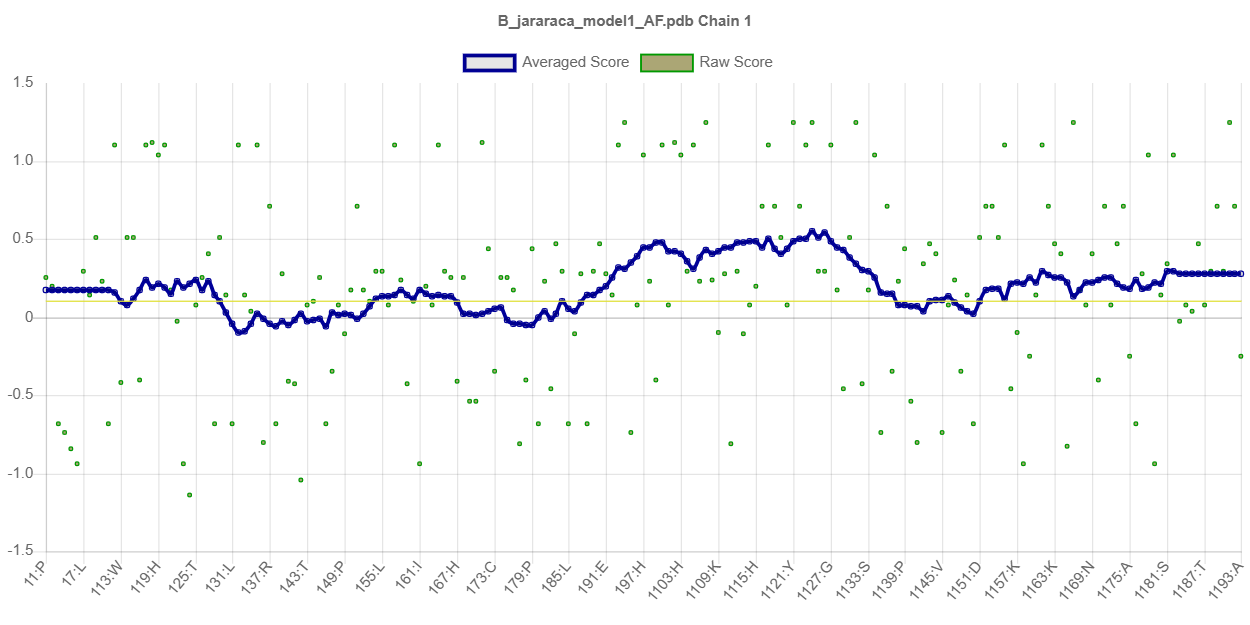

Supplement: Supplementary file 1 [file toxins-17-00262-s001.zip › Supplementary Material 2/File S9 ARSB/B.jararaca/BJ-VF3D.png]
